# Synthesis, Characterization, and Application of a Novel Polystyrene-Supported Brønsted-Acidic Ionic Liquid as an Efficient and Reusable Catalyst in Microwave-Assisted Groebke–Blackburn–Bienaymé Multicomponent Reaction

**DOI:** 10.1021/acsomega.6c03507

**Published:** 2026-06-20

**Authors:** Nicolas S. Anjos, Daniel P. Marques, Sandy J. Coutinho, Fabiana S. F. Borges, Ana Santos, Peter Licence, Luiz S. Longo

**Affiliations:** † Department of Pharmaceutical Sciences, 28105 Federal University of São Paulo—UNIFESP, Rua São Nicolau 210, Diadema, São Paulo 09913-030, Brazil; ‡ School of Chemistry, GSK Carbon Neutral Laboratory, The University of Nottingham-Jubilee Campus, Nottingham NG7 2GA, U.K.

## Abstract

Herein, we report a novel synthesis of the polymer-supported Brønsted-acidic ionic liquid—PS-[(SO_3_H)^4^C_4_Im]­[OTf]—as an efficient and recyclable heterogeneous catalyst for the Groebke–Blackburn–Bienaymé (GBB) multicomponent reaction. The catalyst was synthesized from Merrifield resin (polystyrene) and fully characterized by thermogravimetric analysis (TGA), Fourier transform infrared spectroscopy (FTIR), scanning electron microscopy (SEM), and X-ray photoelectron spectroscopy (XPS). Its catalytic activity was then evaluated in GBB model reactions, with the optimal conditions determined using 50 mg mmol^–1^ of PS-[(SO_3_H)^4^C_4_Im]­[OTf] in ethanol as solvent under microwave heating at 150 °C for 1 h. A diverse library of imidazo-fused heterocycles (e.g., imidazo­[1,2-*a*]­pyridines, imidazo­[2,1-*b*]­thiazoles, and benzo­[*d*]­imidazo­[2,1-*b*]­thiazoles) was synthesized using the optimized conditions and the corresponding products were obtained in moderate to excellent yields (34–91%), depending on the starting aminoazole. Furthermore, the heterogeneous catalyst could be easily recovered by filtration after each reaction cycle and reused for up to six consecutive cycles with no significant loss of integrity as well as catalytic activity (average yield 86 ± 3.5%). These results demonstrated the potential of this polymer-supported Brønsted-acidic ionic liquid as a sustainable catalyst for acid-catalyzed multicomponent reactions applied to the synthesis of nitrogen-based heterocycles of interest in Medicinal Chemistry.

## Graphical abstract

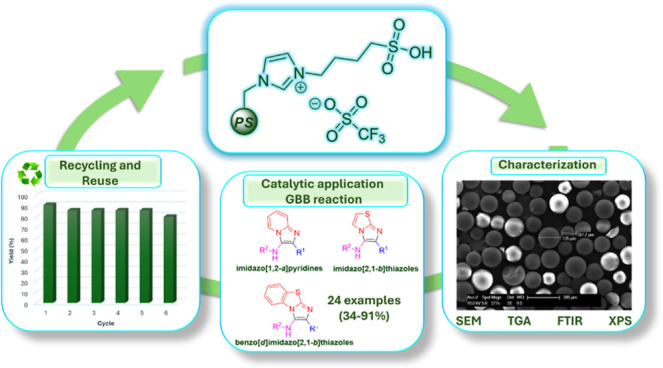

## Introduction

The development of sustainable processes is currently one of the primary goals in organic chemistry, essential for the greener synthesis of high-value compounds such as active pharmaceutical ingredients and polymers.^1^ While the renewable origin of starting materials is crucial, the design of the synthetic methodology itself presents a significant challenge for chemists aiming for more sustainable production standards. In this context, the 12 principles of green chemistry provide fundamental guidelines for modern organic synthesis, contributing to cleaner industrial production.^2^ For instance, modern protocols in the fine-chemical industry must now attempt to avoid stoichiometric reagents, use catalytic approaches, and replace volatile organic solvents with green solvents (or solvent-free reactions), thereby reducing the E-factor of chemical processes.^3^

Multicomponent reactions (MCRs) are powerful, atom-efficient transformations that combine three or more starting materials in a single flask, where multiple bonds are formed in a one-pot operation.^4^ While classical MCRs (such as Strecker, Biginelli, Passerini, and Ugi) date back to the 19th and 20th centuries,^5–8^ the field has experienced a renaissance in recent decades, emerging as a primary tool for the rapid assembly of complex scaffolds.^9,10^ Ideally aligned with green chemistry principles, MCRs promote waste prevention, high atom economy, mild conditions (energy efficiency), and simplified purification steps.^11^ More recently, the application of MCRs with enabling technologies, such as flow chemistry,^12^ photochemistry,^13^ sonochemistry,^14^ and microwave irradiation,^15^ has received significant attention. Furthermore, harnessing intrinsic features of MCRs in combination with green solvents and recyclable catalyst systems can boost the green credentials of the synthetic process, contributing to sustainable chemical manufacturing.^16^

Specifically, the Groebke–Blackburn–Bienaymé (GBB) reaction is an isocyanide-based multicomponent reaction that combines 2-aminoazoles, isocyanides, and aldehydes under acidic catalysis to yield imidazo-fused heterocycles such as imidazo­[1,2-*a*]­pyridines, imidazo­[1,2-*a*]­pyrimidines, imidazo­[2,1-*b*]­thiazoles, benzo­[*d*]­imidazo­[2,1-*b*]­thiazoles, among others.^17–21^ Specially, imidazo­[1,2-*a*]­pyridines, obtained when 2-aminopyridines are used as a 2-aminoazole component, have been recognized as privileged scaffolds in Medicinal Chemistry.^22–26^ Traditionally, GBB reactions are efficiently catalyzed either by Lewis acids, such as Sc­(OTf)_3_,^27–34^ ZrCl_4_,^35,36^ Gd­(OTf)_3_,^37^ BiCl_3_,^38,39^ among others, or Brønsted acids such as HClO_4_,^40–44^ PTSA,^45–48^ AcOH,^49–51^ and HCl.^52–54^ The novel use of Brønsted-acidic ionic liquids based on the 1-(butyl-4-sulfonic acid)-3-methylimidazolium cation as catalysts in the GBB reaction was previously described by our group (Scheme 1).^55^ While the Brønsted acid ionic liquid-catalyzed GBB multicomponent reaction could be efficiently carried out in the presence of catalyst [(SO_3_H)^4^C_4_C_1_Im]­[OTf] (**I**), the procedure for its recovery and recycling proved troublesome: despite maintaining good catalytic activity for four reaction cycles, a gradual decrease in yield was noted. More importantly, the handling of viscous ionic liquids presents significant operational challenges, requiring several solvent extractions and long drying process in vacuo to ensure complete water removal from the catalyst.

**Scheme 1:**
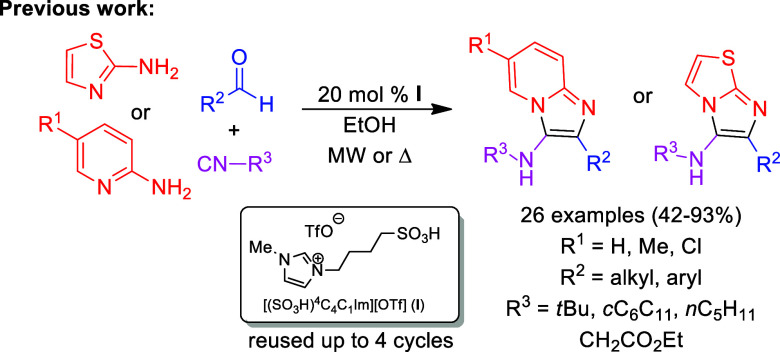
GBB Reaction Catalyzed by Reusable Brønsted-Acidic Ionic Liquids

Alternatively, a viable strategy to overcome the limitations associated with workup procedures involving ionic liquid phases is the direct linkage of the catalyst onto a solid support, so-called supported ionic liquid films (SILFs).^56,57^ The heterogenization of the catalyst/ionic liquid offers distinct advantages for a wide range of organic transformations, most notably the simplified separation of products from the reaction mixture and the straightforward recovery of the heterogeneous catalyst by simple filtration or decantation techniques.^58^ The covalent immobilization of an ionic liquid can be achieved by anchoring it to inorganic supports (e.g., mesoporous silica, magnetic nanoparticles, zeolites) or polymeric matrices (e.g., polystyrene).^59^ Ionic liquids immobilized on polymer resins are commonly classified as polymer-supported ionic liquids (PSILs). Among the polymers suitable for PSIL synthesis, Merrifield resin, comprising styrene and 4-vinylbenzyl chloride monomers, is particularly noteworthy. This material is widely employed for IL immobilization because of its low cost, commercial availability, and well-established physicochemical properties. Recently, Aggarwal and Kumar Chopra^60^ reviewed the versatility of Merrifield resin as a support for the synthesis of structurally diverse PSILs applied in catalysis for various organic reactions.

In alignment with our interest in the development of more sustainable multicomponent synthesis of biological active compounds, as well as to overcome the tedious recovery procedure of homogeneous [(SO_3_H)^4^C_4_C_1_Im]­[OTf] (**I**) previously used by us as a catalyst for the GBB multicomponent reaction,^55^ we describe herein the synthesis and characterization of a novel polystyrene-supported Brønsted-acidic ionic liquid (PS-BAIL) as an efficient and reusable heterogeneous catalyst for the GBB reaction applied to the synthesis of diverse imidazo-fused heterocycles.

## Results and Discussion

### Synthesis and Characterization of PS-[(SO_3_H)^4^C_4_Im]­[OTf]

Our initial efforts were directed toward the immobilization of [(SO_3_H)^4^C_4_C_1_Im]­[OTf] (**I**) in the polystyrene support. The polymer-supported ionic liquid was synthesized according to procedures described in the literature, with adaptations whenever necessary (Scheme 2).^61–64^ First, the alkylation of Merrifield resin (PS-Cl) with imidazole was carried out in refluxing toluene for 24 h, followed by the addition of 1,4-butane sultone under the same conditions. The resulting zwitterionic intermediate PS-[(SO_3_)^4^C_4_Im] (**II**) was then treated with equimolar amounts of HOTf in refluxing dichloromethane for 24 h, affording PS-[(SO_3_H)^4^C_4_Im]­[OTf] (**III**) in quantitative yield. Next, the PS-Cl, the zwitterion **II**, and catalyst **III** were fully characterized by thermogravimetric analysis (TGA), Fourier transform infrared spectroscopy (FTIR), scanning electron microscopy (SEM), and X-ray photoelectron spectroscopy (XPS).

**Scheme 2:**
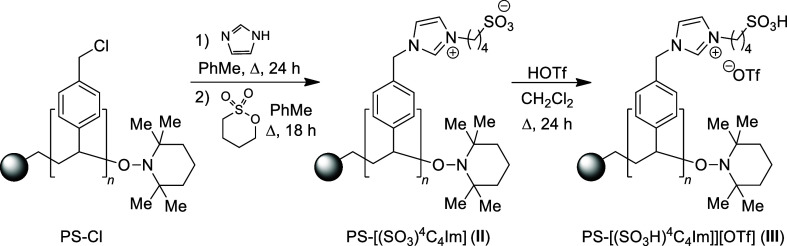
Synthesis of PS-[(SO_3_H)^4^C_4_Im]­[OTf] (**III**)

The TG curves of the three analyzed materials are depicted in Figure 1 and the TG parameters are summarized in Table 1. Two distinct mass loss stages were observed in all curves. For the Merrifield resin (PS-Cl), the first stage resulted in a mass loss of 21.9% at a *T*_onset_ of 291 °C and a *T*_peak_ of 321 °C, possibly attributed to the pyrolysis of the chloromethylene group present in the polymer structure.^65^ The second stage occurred at a *T*_onset_ of 372 °C with a Δ*w* of 32.7%, which may be due to the pyrolysis of the polystyrene carbon backbone.^66^ In the thermograms of **II** and **III**, the first stage occurred at a similar *T*_onset_ for both materials (329 and 316 °C, respectively), albeit with distinct mass loss percentages (49.4% and 32.1%, respectively). This degradation may correspond to the pyrolysis of the cationic moiety of the ionic liquid, which accounts for the difference in mass loss percentage. Polystyrene pyrolysis occurred at *T*_onset_ values of 442 and 477 °C for **II** and **III**, respectively. Accordingly, it was observed that PS-[(SO_3_H)^4^C_4_Im]­[OTf] (**III**) is a heterogeneous ionic liquid with good thermal stability, which is advantageous for catalytic activity in reactions performed at elevated temperatures.

**Figure 1:**
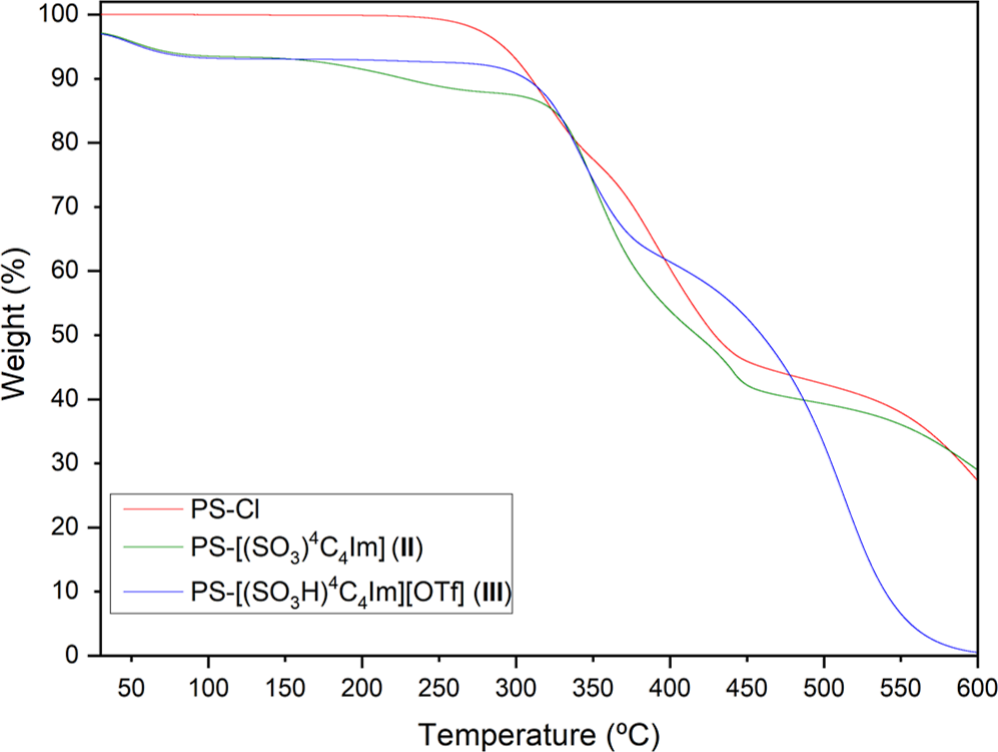
TG curves of PS-Cl, PS-[(SO_3_)^4^C_4_Im] (**II**), and PS-[(SO_3_H)^4^C_4_Im]­[OTf] (**III**).

**Table 1:** Thermogravimetric Analysis for PS-Cl, PS-[(SO_3_)^4^C_4_Im] (**II**), and PS-[(SO_3_H)^4^C_4_Im]­[OTf] (**III**)

	thermogravimetric analysis								
	stage 1				stage 2				residue 600 °C
	Δ*w* (%)	*T*_start_ (°C)	*T*_onset_ (°C)	*T*_peak_ (°C)	Δ*w* (%)	*T*_start_ (°C)	*T*_onset_ (°C)	*T*_peak_ (°C)	*w*
PS-Cl	21.9	203	291	321	32.7	350	372	395	27.4
PS-[(SO_3_)^4^C_4_Im] (**II**)	49.4	298	329	351	14.8	404	442	442	29.1
PS-[(SO_3_H)^4^C_4_Im][OTf] (**III**)	32.1	289	316	337	58.0	421	477	506	0.5

FTIR characterization enabled the identification of absorption bands characteristic of the functional groups present in the analyzed materials (Figure 2). As expected, the Merrifield resin (PS-Cl) spectrum displayed absorption bands attributed to the chloromethyl group (–CH_2_Cl): the C–H bending vibration of the methylene group at 1263 cm^–1^ and the C–Cl stretching vibration at 670 cm^–1^. The absence of these bands in the spectra of the zwitterion **II** and **III** suggests the successful alkylation of the imidazole moiety by the chloromethyl group of the Merrifield resin. Furthermore, both spectra exhibited intense absorption bands in the 1028–1154 cm^–1^ region, corresponding to the symmetric and asymmetric stretching vibrations of the S=O bonds in the –SO_3_H group. Additionally, the spectrum of PS-[(SO_3_H)^4^C_4_Im]­[OTf] (**III**) showed an absorption band at 1255 cm^–1^ attributed to the C–F stretching vibration of the triflate anion.

**Figure 2:**
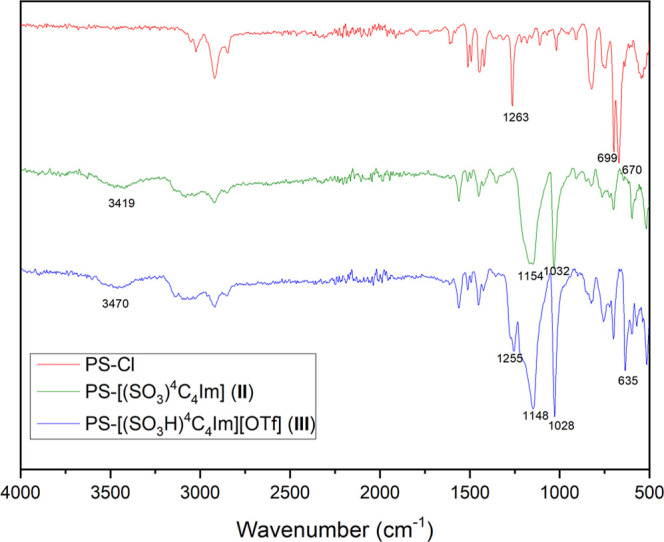
FTIR spectra of PS-Cl, PS-[(SO_3_)^4^C_4_Im] (**II**), and PS-[(SO_3_H)^4^C_4_Im]­[OTf] (**III**).

The microscopic surface morphology of the synthesized materials was analyzed using SEM (Figure 3). Merrifield resin (PS-Cl) is commercially available as spherical beads with a particle size ranging from 75 to 150 μm (100–200 mesh) (Figure 3a). It was observed that this morphology was preserved throughout the resin functionalization steps, as the micrographs of **II** and **III** also displayed uniform microspheres with particle sizes consistent with those observed for the commercial polymer (Figure 3b,c, respectively).

**Figure 3:**
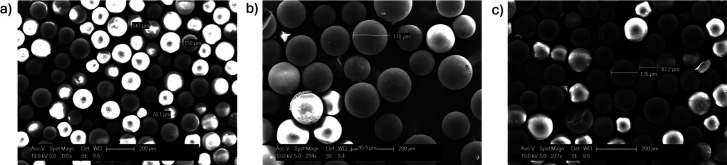
SEM images of (a) PS-Cl, (b) PS-[(SO_3_)^4^C_4_Im] (**II**), and (c) PS-[(SO_3_H)^4^C_4_Im]­[OTf] (**III**).

X-ray Photoelectron Spectroscopy (XPS) was used to study surface composition and chemical states of all elements present within the synthesized materials. By comparing the superimposed survey spectra of PS-Cl, PS-[(SO_3_)^4^C_4_Im] (**II**), and PS-[(SO_3_H)^4^C_4_Im]­[OTf] (**III**), the progression of the catalyst synthesis could be studied (Figure 4). Three main photoemission lines were observed in the survey scan of Merrifield resin (PS-Cl), i.e., peaks for O 1s, C 1s, and Cl 2p, along with a secondary photoemission peak for Cl 2s. Next, successful alkylation of PS-Cl was confirmed by the absence of the Cl 2s and Cl 2p peaks in the survey scan of PS-[(SO_3_)^4^C_4_Im] (**II**). In addition, new photoemission lines for S 2s and S 2p were observed, indicating that the incorporation of the –(CH_2_)_4_SO_3_^–^ side chain was successful. The neutralization of **II** with triflic acid furnished the catalyst PS-[(SO_3_H)^4^C_4_Im]­[OTf] (**III**) and a new photoemission line for F 1s was observed, confirming the incorporation of a triflate anion into the polymer-supported ionic liquid.

**Figure 4:**
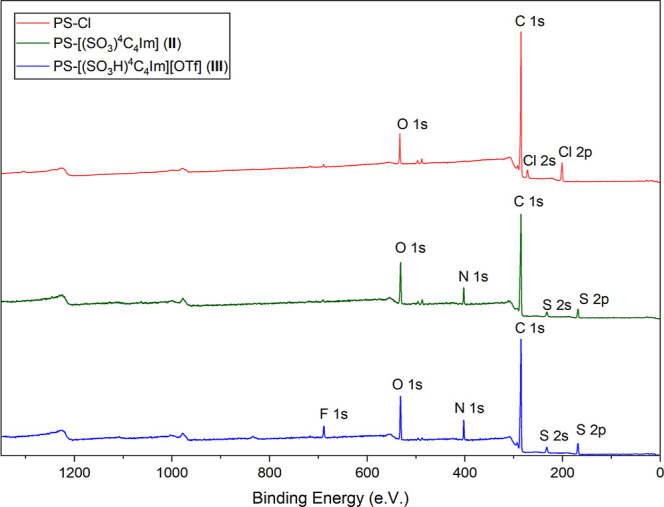
XPS survey of PS-Cl, PS-[(SO_3_)^4^C_4_Im] (**II**), and PS-[(SO_3_H)^4^C_4_Im]­[OTf] (**III**).

The high-resolution spectra for all elements present in the PS-[(SO_3_H)^4^C_4_Im]­[OTf] (**III**) catalyst were further recorded, and spectra are shown in Figure 5. The binding energies for all elements within the sample were charge-corrected by setting the C_PS_ component (polystyrene backbone carbon atoms) in the C 1s high-resolution spectrum to 284.8 eV.^67,68^ Using this charge correction model, the binding energy (BE) values for all photoemission peaks were reproducible within an experimental error of ±0.1 eV. Given the presence of different carbon environments in the cationic heads, a fitting model for the C 1s region was proposed. Initially, a four-component model [i.e., C_2_, (C_4_ + C_5_), (C_6_ + C_7_), and C_PS_] was considered, taking into account a previous fitting model for 1-alkyl-3-methylimidazolium ionic liquids proposed by us.^69^ However, this model failed to yield binding energies that correlated well with those observed for similar [C_*n*_C_1_Im]­[OTf] ionic liquids (where *n* = 2, 4, 8, 12). This discrepancy is likely due to the nature of the –(CH_2_)_4_SO_3_H side chain in **III**, since the C8, C9, and C10 atoms cannot be considered purely aliphatic in this ionic liquid. Consequently, a fifth component was assigned to (C_8_ + C_9_ + C_10_) (Figure 5a). With this modification, a good correlation of binding energies across all C 1s components was achieved for both [C_4_C_1_Im]­[OTf][Bibr ref69] and PS-[(SO_3_H)^4^C_4_Im]­[OTf] (**III**) ionic liquids (Table 2). Ultimately, the new (C_8_ + C_9_ + C_10_) 1s component was observed at 285.4 eV, which is very close to the binding energy previously reported by Villar-Garcia et al.^69^ for the butyl side chain in the [C_4_C_1_Im]­[OTf] ionic liquid (i.e., 285.2 eV).

**Figure 5:**
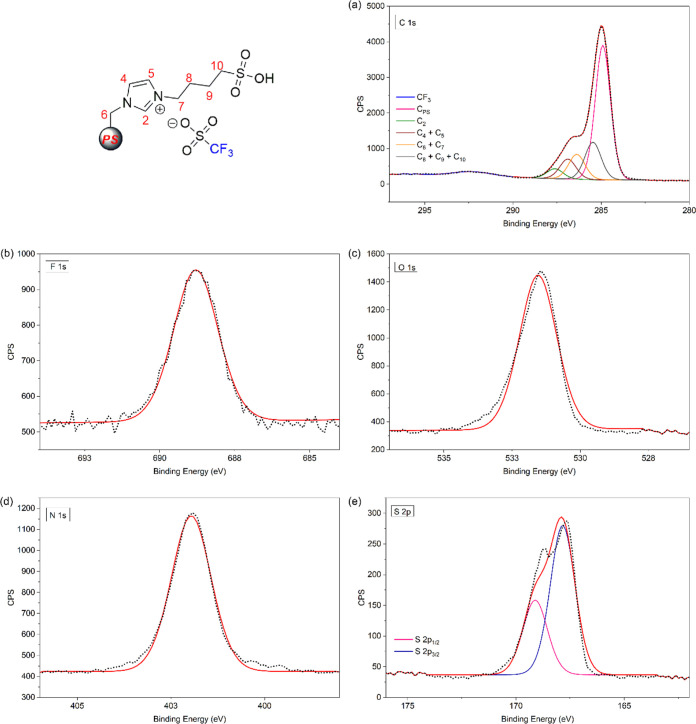
High-resolution XP spectra of PS-[(SO_3_H)^4^C_4_Im]­[OTf] (**III**) for: (a) C 1s, (b) F 1s, (c) O 1s, (d) N 1s, (e) S 2p. The binding energies for all elements were charge corrected by setting the C_PS_ component to 284.8 eV. The associated experimental error is ±0.1 eV.

**Table 2:** Experimental Binding Energies (eV) Obtained for PS-[(SO_3_H)^4^C_4_Im]­[OTf] (**III**)­^a^

	binding energy (eV)^b^	
	PS-[(SO_3_H)^4^C_4_Im][OTf] (**III**)	[C_4_C_1_Im][OTf][Bibr ref69]
F 1s	688.6	688.5
O 1s	531.4	532.0
N 1s	401.9	402.0
CF_3_ 1s	292.2	292.5
C_2_ 1s	287.5	287.6
(C_4_ + C_5_) 1s	286.8	286.9
(C_6_ + C_7_) 1s	286.3	286.5
(C_8_ + C_9_ + C10) 1s	285.4	-
C_PS_ 1s	284.8	-
C_aliph_ 1s	-	285.2
S 2p_3/2_	167.7	168.4

a The binding energies for all elements were charge corrected by setting the C_PS_ component to 284.8 eV.

b The associated experimental error is ±0.1 eV.

The F 1s peak for the triflate anion in the catalyst **III** was observed at 688.6 eV (Figure 5b), a value in good agreement with those previously reported for the triflate anion in the series of [C_*n*_C_1_Im]­[OTf] ionic liquids (i.e., 688.5 eV).^69^ The analysis of the O 1s region proved challenging due to the distinct electronic environments of the oxygen atoms in the –SO_3_H side chain and TfO^–^ anion. Unfortunately, we were unable to develop a reliable deconvolution model to separate these two oxygen electronic components. Instead, a single-component model was applied, yielding a mean unresolved binding energy of 531.4 eV (Figure 5c). Notably, the O 1s peak for the triflate anion in [C_*n*_C_1_Im]­[OTf] ionic liquids has been reported at 532.0 eV.^69^ Cao et al.^70^ studied the XPS of 3-methyl-1-(4-sulfonic acid)­butyl imidazolium bisulfate –[(SO_3_H)^4^C_4_Im]­[HSO_4_]– and recorded an O 1s peak at 531.7 eV; this unresolved peak was attributed to the oxygen atoms present in –SO_3_H and SO_4_^2–^ groups.

The N 1s peak in PS-[(SO_3_H)^4^C_4_Im]­[OTf] (**III**) was observed at 401.9 eV (Figure 5d), also matching the N 1s value obtained previously for [C_*n*_C_1_Im]­[OTf] ionic liquids (i.e., 402.0 eV). Zhang et al.^71^ prepared Brønsted acidic ionic liquid-functionalized ethyl-bridged organosilica hollow nanospheres (HNSs) as acidic catalysts for esterification reactions. Analysis of [(SO_3_H)^3^C_3_Im]­[OTf]-Si­(Et)Si HNSs using XPS revealed that the N 1s peak of imidazolium cationic heads appeared at 401.8 eV. This confirms that the alkyl chain could have a negligible impact on the binding energy of the cationic N 1s core level, when the anion remains constant across a series of 1,3-dialkylimidazolium ionic liquids.^69,72^ Finally, analysis of the S 2p region revealed an overlap as previously observed in the O 1s spectrum. Unfortunately, we could not resolve the distinct electronic environments of the sulfur atoms present in the –SO_3_H side chain and TfO^–^ anion. The simplest deconvolution model accounted for the S 2p_3/2_ and 2p_1/2_ spin–orbit splitting of the observed doublet, with the main S 2p_3/2_ photoemission peak appearing at 167.7 eV (Figure 5e). The observation of a single doublet with an S 2p_3/2_/2p_1/2_ spin–orbit splitting of 1.3 ± 0.1 eV further illustrates the unresolved nature of these two sulfur species. Previous study reported by Zhang et al.^71^ also attributed an unresolved peak centered at 168.3 eV for S 2p_3/2_ from the propyl SO_3_H group and TfO^–^ anion from [(SO_3_H)^3^C_3_Im]­[OTf]-Si­(Et)­Si hollow nanospheres.

### Catalytic Activity Evaluation of PS-[(SO_3_H)^4^C_4_Im]­[OTf]

The catalytic activity of the heterogeneous Brønsted-acidic ionic liquid PS-[(SO_3_H)^4^C_4_Im]­[OTf] (**III**) was evaluated using a GBB model reaction between 2-aminopyridine, benzaldehyde, and *tert*-butyl isocyanide to afford the imidazo­[1,2-*a*]­pyridine **1a** (Table 3). Initially, the uncatalyzed reaction was carried out using ethanol as solvent at room temperature for 24 h, affording the product **1a** in only 16% yield (entry 1). However, **1a** was obtained in 52% yield when the reaction was conducted under the same conditions but in the presence of 100 mg mmol^–1^ of catalyst **III** (entry 2). Aiming to optimize this methodology, subsequent experiments were conducted under microwave irradiation to reduce reaction times and improve yields. Next, a screening of solvents commonly employed in GBB reactions was performed using 100 mg mmol^–1^ of **III** at 100 °C for 4 h (entries 3–6). Under these conditions, ethanol was identified as the best solvent, leading to the formation of the desired imidazopyridine in 79% yield. Thus, it was observed that product **1a** could be isolated up to 90% yield when the reaction was carried out in ethanol at higher temperatures (entry 8). Finally, the influence of the catalyst loading on the model reaction was investigated. It was observed that reducing the loading to 50 mg mmol^–1^ of **III** enhanced catalytic activity, and **1a** was obtained in 91% yield (entry 10). In contrast, a further reduction to 25 mg mmol^–1^ of **III** proved inefficient, leading to a decrease in yield to ca. 49% (entry 12). For comparison, the use of commercially available Amberlyst 15 as a heterogeneous Brønsted acidic catalyst (50 mg mmol^–1^) for this model reaction led to **1a** in only 52% yield (entry 13).

**Table 3:**
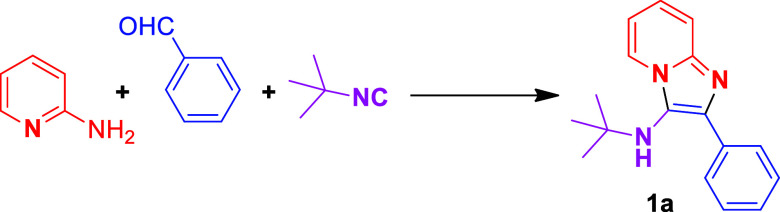
Catalytic Activity Evaluation of PS-[(SO_3_H)^4^C_4_Im]­[OTf] (**III**) in the GBB Model Reaction^a^

entry	catalyst loading (mg mmol^–1^)	solvent	temperature (°C)	time (h)	yield (%)^b^
1	-	EtOH	R.T.	24	16
2	(**III**) (100)	EtOH	R.T.	24	52
3	(**III**) (100)	EtOH	100 (MW)	4	79
4	(**III**) (100)	2-Me-THF	100 (MW)	4	31
5	(**III**) (100)	MeCN	100 (MW)	4	72
6	(**III**) (100)	PhMe	100 (MW)	4	65
7	(**III**) (100)	EtOH	120 (MW)	4	82
8	(**III**) (100)	EtOH	150 (MW)	4	90
9	(**III**) (100)	EtOH	150 (MW)	1	70
10	(**III**) (50)	EtOH	150 (MW)	1	91
11	(**III**) (50)	EtOH	150 (MW)	0.5	75
12	(**III**) (25)	EtOH	150 (MW)	1	49
13	Amberlyst 15 (50)	EtOH	150 (MW)	1	52

a Reagents and conditions: 2-aminopyridine (1.0 mmol), benzaldehyde (1.0 mmol), *tert*-butyl isocyanide (1.0 mmol), catalyst (25–100 mg.mmol^–1^), solvent (3.0 mL), MW = microwave (CEM Discover, sealed tube), R.T. = room temperature.

b Isolated yields.

Thus, the optimized reaction conditions for this GBB model reaction under microwave irradiation were established as those shown in entry 10 (Table 3) and further used to investigate substrate scope for GBB MCR applied to the synthesis of a diverse library of imidazo-fused heterocycles. Initially, the influence of electron-donating groups (EDG) and -withdrawing groups (EWG) on the reactivity of *p*-substituted benzaldehydes was investigated for the preparation of imidazo­[1,2-*a*]­pyridines bearing different substituents at the C2 position (Figure 6). It was observed that the reactions carried out using both EDG or EWG substituents afforded the corresponding imidazo­[1,2-*a*]­pyridines **1b**–**1d** in high yields (85–87%). Similarly, the reaction between 2-aminopyridine, *tert*-butyl isocyanide, and furfural was carried out under the optimized conditions, furnishing **1e** in 85% yield. Likewise, this protocol proved to be efficient for the synthesis of **1f** (88% yield) when aliphatic butyraldehyde was employed.

**Figure 6:**
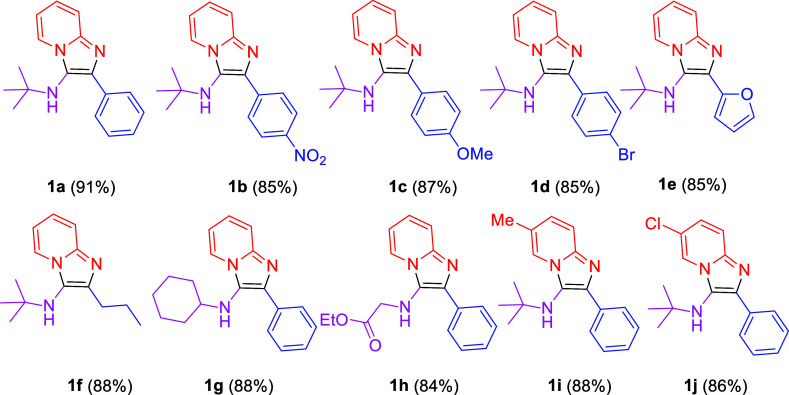
Synthesis of imidazo­[1,2-*a*]­pyridines **1a**–**j** via the GBB reaction catalyzed by PS-[(SO_3_H)^4^C_4_Im]­[OTf] (**III**). Reagents and conditions: 2-aminoazole (1.0 mmol), aldehyde (1.0 mmol), isocyanide (1.0 mmol), ethanol (3.0 mL), PS-[(SO_3_H)^4^C_4_Im]­[OTf] (**III**) (50 mg.mmol^–1^), microwave (CEM Discover or Anton Paar Monowave 300, sealed tube), 150 °C, 1 h.

The use of different isocyanides was then further explored. It was observed that the replacement of *tert*-butyl isocyanide with cyclohexyl isocyanide or ethyl isocyanoacetate did not significantly impact the reaction efficiency, and the corresponding imidazo­[1,2-*a*]­pyridines were obtained in high yields (**1g**, 88% and **1h**, 84%). At this point, the synthesis of imidazo­[1,2-*a*]­pyridines substituted at the C6 position, varying 2-aminoazole components, was also investigated. In this case, the reactions employing 6-amino-3-picoline (2-amino-5-methylpyridine), 2-amino-5-chloropyridine, *tert*-butyl isocyanide, and benzaldehyde under the optimized conditions led to the formation of products **1i** and **1j** in 88% and 86% yields, respectively.

The study of the substrate scope was further expanded toward the synthesis of less exploited imidazo-fused heterocycles via the GBB reaction, such as imidazo­[2,1-*b*]­thiazoles and benzo­[*d*]­imidazo­[2,1-*b*]­thiazoles. When 2-aminopyridine was replaced by 2-aminothiazole as 2-aminoazole component, it was observed that the reactions proceeded more slowly (compared to those yielding imidazo­[1,2-*a*]­pyridines), requiring at least 3 h of microwave heating at 150 °C to achieve full consumption of the starting materials. For instance, reactions employing 2-aminothiazole, benzaldehyde or isovaleraldehyde, and various isocyanides furnished the corresponding imidazo­[2,1-*b*]­thiazoles **2a–d** in moderate to good yields (61–74%) (Figure 7). The reactivity of substituted 2-aminothiazoles in the GBB reaction was also explored. In this context, the use of 5-methyl-2-aminothiazole showed similar reactivity compared to the unsubstituted 2-aminothiazole, yielding products **2e** and **2f** in 75% and 56% yields, respectively. However, lower reactivity was observed when 4-methyl-2-aminothiazole was employed, and the products **2g** and **2h** were isolated in only 34% and 55% yields, respectively. Notably, regarding the synthesis of **2g**, the intermediate imine formed from the condensation of 4-methyl-2-aminothiazole and *p*-anisaldehyde was isolated in 22% yield, highlighting the reduced reactivity of this intermediate toward the nucleophilic attack of the isocyanide. This finding corroborates previous reports by Bienaymé,^19^ wherein the low reactivity of electron-poor 2-aminoazoles (such as 2-aminothiazoles) favors the accumulation of the Schiff base intermediate, ultimately decreasing the overall yield for the imidazo heterocycle.

**Figure 7:**
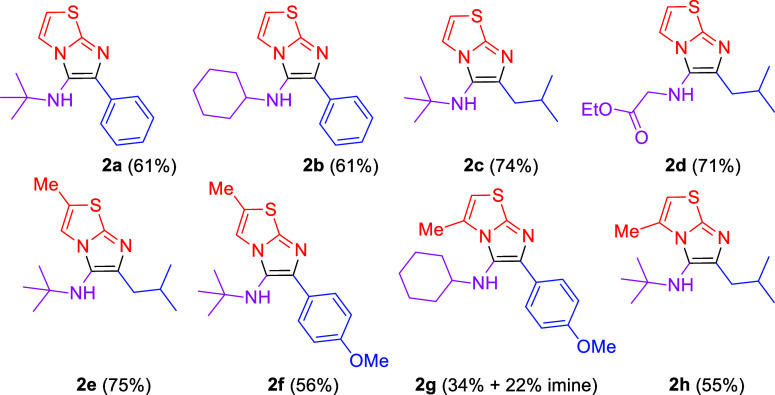
Synthesis of imidazo­[2,1-*b*]­thiazoles **2a**–**h** via the GBB reaction catalyzed by PS-[(SO_3_H)^4^C_4_Im]­[OTf] (**III**). Reagents and conditions: 2-aminoazole (1.0 mmol), aldehyde (1.0 mmol), isocyanide (1.0 mmol), ethanol (3.0 mL), PS-[(SO_3_H)^4^C_4_Im]­[OTf] (**III**) (50 mg.mmol^–1^), microwave (CEM Discover or Anton Paar Monowave 300, sealed tube), 150 °C, 3 h.

Next, this methodology was applied to the synthesis of a library of benzo­[*d*]­imidazo­[2,1-*b*]­thiazoles by employing 2-aminobenzothiazole as the 2-aminoazole component in the GBB reaction (Figure 8). Like the 2-aminothiazole series, reactions utilizing 2-aminobenzothiazole proceeded slowly. In this context, the reactions between 2-aminobenzothiazole, benzaldehyde or isovaleraldehyde, and *tert*-butyl isocyanide were performed under microwave irradiation in the presence of 50 mg of PS-[(SO_3_H)^4^C_4_Im]­[OTf] (**III**) in ethanol at 150 °C for 3 h, affording products **3a–b** in good yields (74–83%). However, transformations involving 2-aminobenzothiazoles substituted at the C6 position (–Me or –Cl) were less efficient compared to those using unsubstituted 2-aminobenzothiazole. In these cases, reactions employing 2-amino-6-methylbenzothiazole with aromatic and aliphatic aldehydes and *tert*-butyl isocyanide and cyclohexyl isocyanide yielded products **3c** and **3d** in 61% and 66% yield, respectively, whereas the use of 2-amino-6-chlorobenzothiazole led to the formation of benzo­[*d*]­imidazo­[2,1-*b*]­thiazoles **3e** and **3f** in slightly higher yields (73% and 68%, respectively).

**Figure 8:**
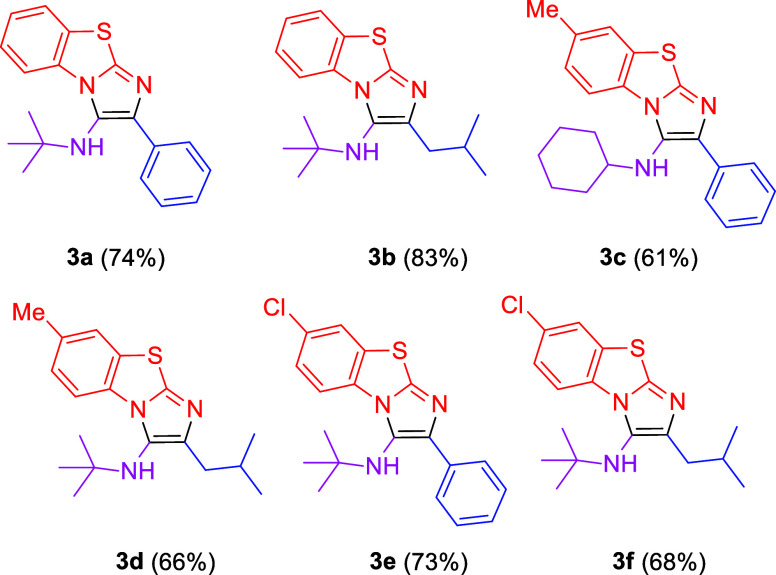
Synthesis of benzo­[*d*]­imidazo­[2,1-*b*]­thiazoles **3a**–**f** via the GBB reaction catalyzed by PS-[(SO_3_H)^4^C_4_Im]­[OTf] (**III**). Reagents and conditions: 2-aminoazole (1.0 mmol), aldehyde (1.0 mmol), isocyanide (1.0 mmol), ethanol (3.0 mL), PS-[(SO_3_H)^4^C_4_Im]­[OTf] (**III**) (50 mg.mmol^–1^), microwave (CEM Discover or Anton Paar Monowave 300, sealed tube), 150 °C, 3 h.

A plausible reaction mechanism for the Groebke–Blackburn–Bienaymé MCR catalyzed by the heterogeneous ionic liquid PS-[(SO_3_H)^4^C_4_Im]­[OTf] (**III**), exemplified for imidazo­[1,2-*a*yridine**1a**, is depicted in Scheme 3. Initially, an intermediate iminium cation is formed after the condensation of the aldehyde and 2-aminopyridine, a step catalyzed by the Brønsted acidic ionic liquid. Subsequently, the iminium cation undergoes nucleophilic attack by the isocyanide, leading to intermediate **IV**. This species undergoes cyclization via the attack of the aminoazole ring nitrogen on the electrophilic carbon of the isocyanide moiety yielding intermediate **V**, further regenerating the acidic catalyst. Formally, this step corresponds to a nonconcerted [4 + 1] dipolar cycloaddition. Finally, the intermediate **V** undergoes a 1,3-H shift to furnish the desired imidazo­[1,2-*a*]­pyridine **1a**.^17,20,73^

**Scheme 3:**
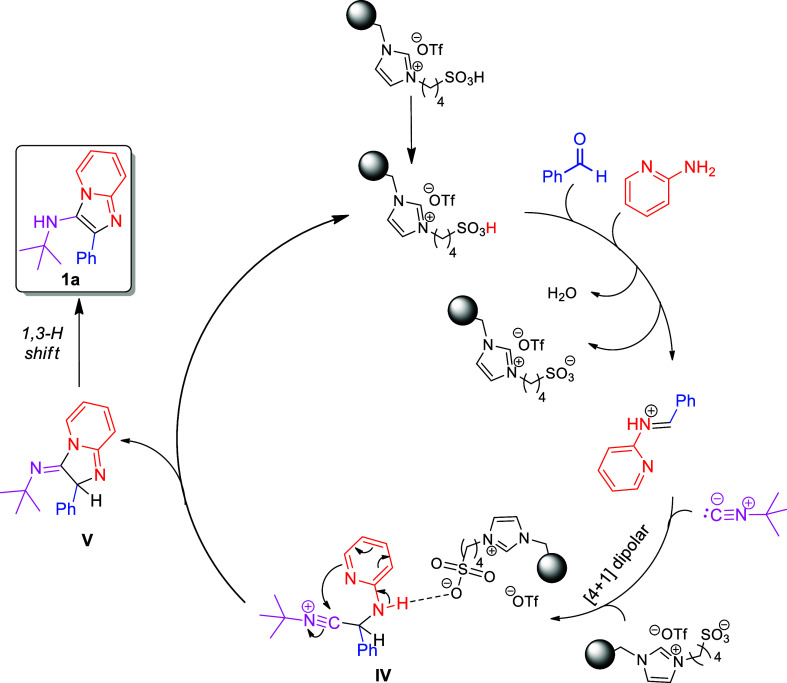
Proposed Mechanism for GBB MCR Catalyzed by PS-[(SO_3_H)^4^C_4_Im]­[OTf] (**III**) (Exemplified for **1a**)

### Recycling and Reuse of PS-[(SO_3_H)^4^C_4_Im]­[OTf]

Being able to establish the best reaction conditions and scope for the GBB MCR catalyzed by PS-[(SO_3_H)^4^C_4_Im]­[OTf] (**III**), we next evaluated its recovery and reuse (Table 4). Initially, the model reaction between 2-aminopyridine, *tert*-butyl isocyanide, and benzaldehyde was carried out under optimized conditions (entry 10, Table 3). Upon reaction completion, the heterogeneous catalyst was isolated from the reaction mixture by vacuum filtration, washed with methanol, and dried in an oven at 60 °C for 1 h. The filtrate was concentrated under reduced pressure, and the crude product was purified by flash column chromatography affording **1a** in 91% yield. After weighing the recovered catalyst, it was subjected to a subsequent GBB reaction run. Using this recovery protocol, PS-[(SO_3_H)^4^C_4_Im]­[OTf] (**III**) could be reused for at least for six consecutive cycles without significant loss of catalytic activity, providing imidazo­[1,2-*a*]­pyridine **1a** in high yields (91%, 86%, 86%, 86%, 86%, and 80%, respectively). It is noteworthy that, despite a minor loss in the recovered mass of **III** over the runs (Table 4), mainly due to handling during filtration and drying procedures, its catalytic efficiency was maintained and **1a** could be isolated in an average yield of 86 ± 3.5%.

**Table 4:** Recyclability and Reuse Study for the Catalyst PS-[(SO_3_H)^4^C_4_Im]­[OTf] (**III**) in the GBB Reaction for the Synthesis of Imidazo­[1,2-*a*]­pyridine **1a** ^a^

entry	cycle	catalyst recovered (mg)^b^	yield of **1a** (%)^c^
1	first	100	91
2	second	99	86
3	third	98	86
4	fourth	96	86
5	fifth	95	86
6	sixth	93	80

a Reagents and conditions: 2-aminopyridine (2.0 mmol), benzaldehyde (2.0 mmol), *tert*-butyl isocyanide (2.0 mmol), ethanol (6.0 mL), catalyst PS-[(SO_3_H)^4^C_4_Im]­[OTf] (**III**) (50 mg.mmol^–1^), microwave (CEM Discover, sealed tube), 150 °C, 1 h.

b The catalyst mass for the next reaction was adjusted to 100 mg.

c Isolated yields.

After the recyclability tests, the catalyst PS-[(SO_3_H)^4^C_4_Im]­[OTf] (**III**) was characterized to evaluate the impact of the reaction conditions on the integrity of the polymer-supported ionic liquid phases (Figure 9). The FTIR analysis revealed the preservation of the absorption bands found in the spectrum of the fresh catalyst, including the C–F stretching vibration (1257 cm^–1^) and the asymmetric and symmetric S=O stretching vibrations (1148 cm^–1^ and 1031 cm^–1^, respectively) (Figure 9a), indicating that the triflate anion is still present. The XPS survey spectrum of the recovered **III** displayed the expected photoemission lines for all elements present within the sample (i.e., F 1s, O 1s, N 1s, C 1s, S 2s, and S 2p). Additionally, new photoemission lines were observed, being attributed to silicon-based contaminants (Si 2s and Si 2p), possibly originating from silicone grease used during the extensive handling of laboratory glassware (Figure 9b). However, considering the consistent yields obtained across the reuse cycles, it is clear to us that this slight contamination did not compromise its catalytic efficiency. Finally, SEM micrographs confirmed the preservation of the characteristic spheroidal morphology and micrometric particle size of the catalyst **III** (75–150 μm), suggesting that the established reaction conditions (150 °C, MW) and recovery/reuse protocols did not significantly change the physical structure of the material (Figure 9c).

**Figure 9:**
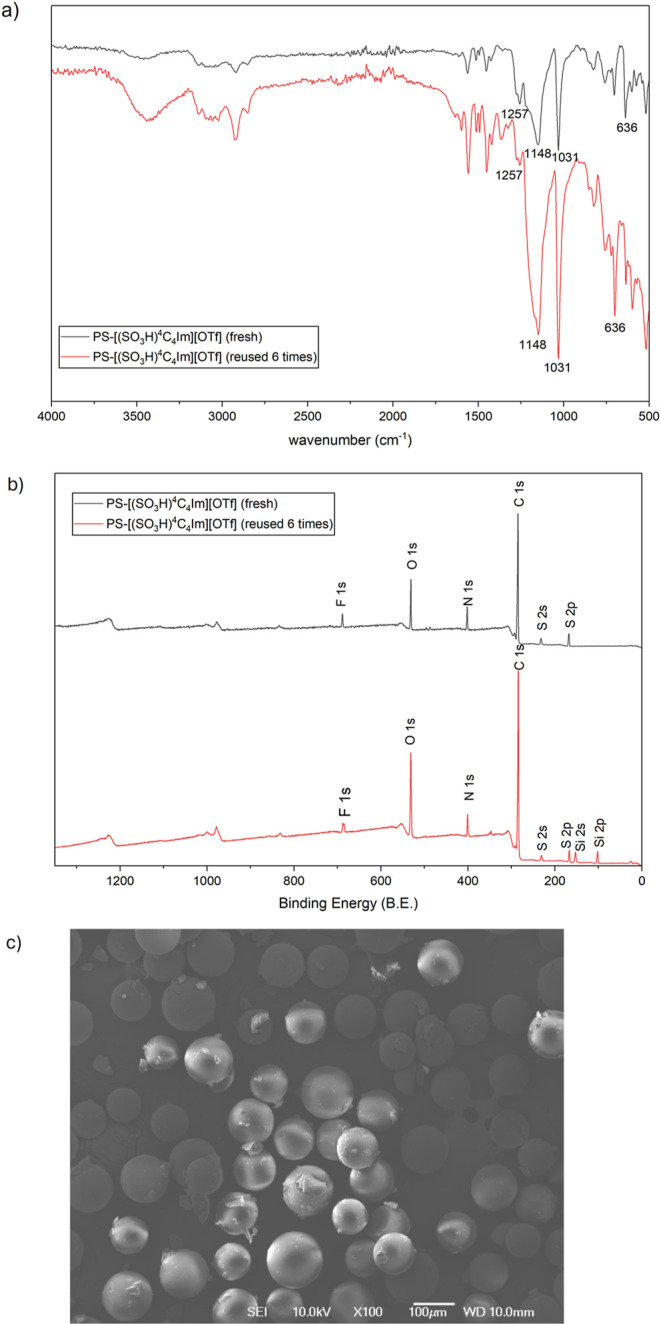
Characterization of PS-[(SO_3_H)^4^C_4_Im]­[OTf] (**III**) after recyclability tests. (a) FTIR spectra; (b) XPS survey spectra; (c) SEM image.

Finally, we scaled up this GBB multicomponent reaction catalyzed by PS-[(SO_3_H)^4^C_4_Im]­[OTf] (**III**) to a gram scale (10-fold scale). Then, the reaction between 2-aminopyridine, *tert*-butyl isocyanide, and benzaldehyde in ethanol with 50 mg mmol^–1^ of **III** at 150 °C under microwave irradiation efficiently furnished pure imidazo­[1,2-*a*]­pyridine **1a** in 94% isolated yield, after recrystallization of the crude product using dichloromethane and petroleum ether (Scheme 4).

**Scheme 4:**
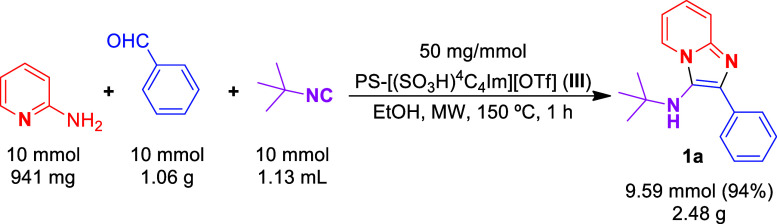
Gram-Scale GBB Reaction Catalyzed by PS-[(SO_3_H)^4^C_4_Im]­[OTf] (**III**)

Homogeneous and heterogeneous Brønsted acids have been explored as catalysts for the GBB reaction applied to the synthesis of imidazo­[1,2-*a*]­pyridines (Table 5). Several research groups described the use of conventional organic and inorganic Brønsted acids such as HClO_4_, HCl, AcOH, and PTSA as efficient catalysts under room temperature or under microwave heating (entries 1–6 and 10). However, notable drawbacks include the explosive nature of HClO_4_ and the use of dioxane as a nongreen solvent when the reaction is catalyzed by HCl. Furthermore, stoichiometric amounts or high catalyst loadings of PTSA, AcOH, and HCl are often required to achieve full conversion of starting materials. The GBB reaction can also be catalyzed by heterogeneous catalytic systems, such as acidic cellulose or CuFe_2_O_4_@SiO_2_–OSO_3_H and Fe_2_O_3_@SiO_2_–OSO_3_H nanoparticles. In these cases, imidazo­[1,2-*a*]­pyridines were obtained in good yields using the optimized conditions (entries 11–13). Additionally, these methodologies demonstrated catalyst recyclability for up to six cycles without significant loss of activity.

**Table 5:** GBB Reaction Catalyzed by Different Homogeneous and Heterogeneous Brønsted Acids for the Synthesis of Imidazo­[1,2-*a*]­pyridines

entry	catalyst	conditions	examples (yield range)	references
1	5 mol % HClO_4_	MeOH, r.t., 0.5–24 h	13 examples (30–80%)	^40^
2	5 mol % HClO_4_	MeOH, r.t., 4 h	22 examples (22–70%)	^41^
3	52 mol % PTSA	MeOH, r.t., 2 h	10 examples (88–97%)	^45^
4	20 mol % PTSA	MeOH, r.t., 18 h	7 examples (65–93%)	^46^
5	1–2 equiv AcOH	MeOH, r.t., 18 h	17 examples (24–80%)	^50^
6	4 N HCl	dioxane, MW, 100 °C, 20 min	9 examples (yields not given)	^52^
7	20 mol % TFA	EtOH, 60 °C, 2 h	14 examples (8–87%)	^74^
8	1 equiv NH_4_Cl	MeOH, r.t., 3 h	10 examples (60–96%)	^75^
9	25 mol % ClCH_2_CO_2_H	MeOH, MW, 100 °C, 1 h	16 examples (61–98%)	^76^
10	HClO_4_	eucalyptol, r.t., 1–12 h	21 examples (39–99%)	^43^
11	2 mol % H_3_PW_12_O_40_ (HPW)	EtOH, MW, 120 °C, 30 min	68 examples (23–99%)	^77^
12	20 mol % [(SO_3_H)^4^C_4_C_1_Im][OTf] (**I**), reuse: 4 cycles	EtOH, MW, 150 °C 1–4 h	22 examples (42–93%)	^55^
13	50 mg mmol^–1^ acid cellulose reuse: 5 cycles	MeOH, r.t., 2 h	10 examples (87–98%)	^78^
14	28 mg mmol^–1^ CuFe_2_O_4_@SiO_2_–OSO_3_H, reuse: 6 cycles	EtOH, reflux, 10 min	17 examples (90–97%)	^79^
15	50 mg mmol^–1^ Fe_2_O_3_@SiO_2_–OSO_3_H, reuse: 5 cycles	solvent-free, 35 °C, 45–70 min	12 examples (85–94%)	^80^
16	50 mg mmol^–1^ PS-[(SO_3_H)^4^C_4_Im][OTf] (**III**), reuse: 6 cycles	EtOH, MW, 150 °C, 1 h	10 examples (84–91%)	this work

Previously, we developed a methodology for the synthesis and application of reusable homogeneous Brønsted-acidic ionic liquids—[(SO_3_H)^4^C_4_Im]­[OTf] (**I**)—as catalysts for the microwave-assisted GBB MCR (entry 12). The novelty of the present work lies in the operational simplicity of recovering and reusing the catalyst by immobilizing the ionic liquid onto a polystyrene support. Indeed, PS-[(SO_3_H)^4^C_4_Im]­[OTf] (**III**) was able to efficiently catalyze the GBB reaction in ethanol under microwave heating yielding a diverse library of different imidazo-fused heterocycles (entry 16). In addition, the catalyst was easily recovered by vacuum filtration and reused for up to six cycles with sustained catalytic activity and physicochemical integrity, confirmed by standard postreaction characterization procedures.

## Conclusions

In summary, we have described an unprecedented methodology for the microwave-assisted Groebke–Blackburn–Bienaymé multicomponent reaction catalyzed by a heterogeneous and reusable polymer-supported Brønsted-acidic ionic liquid. The novel catalyst PS-[(SO_3_H)^4^C_4_Im]­[OTf] (**III**) was synthesized starting from unexpensive Merrifield resin and fully characterized by TGA, FTIR, SEM, and XPS. The catalytic activity of **III** was evaluated in a GBB MCR and the optimized reaction condition was applied for the synthesis of diverse imidazo-fused heterocycles, including imidazo­[1,2-*a*]­pyridines, imidazo­[2,1-*b*]­thiazoles, and benzo­[*d*]­imidazo­[2,1-*b*]­thiazoles, which were obtained in moderate to excellent yields (34–91%), depending on the reactivity of the 2-aminoazole component. Many of the synthesized compounds are described herein for the first time and represent privileged scaffolds in Medicinal Chemistry; further investigation of antitumor properties of such compounds is in due course. In addition, PS-[(SO_3_H)^4^C_4_Im]­[OTf] (**III**) could be recycled for up to six reaction cycles with sustained catalytic activity, without loss of integrity. The robustness and operational simplicity of recovering catalyst by filtration, coupled with high catalytic activity and reusability, highlight this heterogeneous catalytic system as a valuable tool for sustainable acid-catalyzed multicomponent reactions.

## Experimental Section

### General Methods

The solvents used in this work were commercially obtained from various suppliers (Merck, Acros, Aldrich, Fluka, Synth, and Vetec) and were used as received, unless otherwise noted in the experimental procedures. All other reagents were purchased from Sigma-Aldrich and used without further purification. Caution: isocyanides possess a pungent odor and are toxic; therefore, they must be handled with care inside a fume hood. Reactions were performed in an Anton Paar Monowave 300 or CEM Discover 1.0 microwave reactors, using sealed tubes with temperature, power, and pressure control. Thin layer chromatography (TLC) analyses were carried out using silica gel plates 60 F254 from Merck and UV-light, vanillin, or *p*-anisaldehyde solutions for visualization. 1H and 13C nuclear magnetic resonance (NMR) spectra were recorded at room temperature on Bruker DPX300, DPX400, or AV400 spectrometers, using DMSO-*d*_6_ or CDCl_3_ as solvents. Chemical shifts (δ) are expressed in ppm and referenced to the residual solvent peak; coupling constants are expressed in hertz (Hz). High-resolution mass spectrometry (HRMS) analyses were carried out using a Bruker MicroTOF 61 spectrometer [electrospray ionization method, ESI­(+)]. Melting point ranges were determined using a Buchi 545 MP melting point apparatus. Infrared spectra were recorded on a PerkinElmer 1600 FT or Shimadzu (IR Prestige-21) spectrometer.

### Synthesis and Characterization of PS-[(SO_3_H)_4_C_4_Im]­[OTf] (**III**)

Merrifield resin (100–200 mesh, 3.5–4.5 mmol Cl^–^·g^–1^) (10 g), imidazole (15.3 g, 225 mmol, 5 equiv; considering 4.5 mmol of Cl^–^·g^–1^ initial loading), and toluene (250 mL) were added to a 500 mL round-bottom flask. The suspension was maintained under reflux for 24 h, followed by the slow addition of 1,4-butane sultone (28.8 mL, 38.6 g, 283.5 mmol, 6.3 equiv). The reaction mixture was further stirred under reflux for additional 18 h. Upon completion and cooling, the suspension was filtered under vacuum, and the resulting solid PS-[(SO_3_)_4_C_4_Im] (**II**) was washed with successive portions of EtOH/H_2_O (1:1, 100 mL), MeOH (100 mL), and diethyl ether (100 mL), followed by drying under high vacuum (10^–3^ mbar) for 18 h.

Subsequently, zwitterion PS-[(SO_3_)^4^C_4_Im] (**II**) (10 g) was suspended in dichloromethane (60 mL) in a 250 mL round-bottom flask. In a separate beaker containing dichloromethane (40 mL), triflic acid (HOTf) (4.0 mL, 6.75 g, 45 mmol, 1 equiv) was added slowly. The HOTf solution was then slowly poured into the zwitterion suspension, and the reaction mixture was maintained at reflux for 18 h. Upon completion and cooling, the mixture was filtered under vacuum, and the obtained solid was washed with successive portions of EtOH/H_2_O (1:1, 100 mL), MeOH (100 mL), and diethyl ether (100 mL), followed by drying under high vacuum (10^–3^ mbar) for 18 h. The ionic liquid PS-[(SO_3_H)^4^C_4_Im]­[OTf] (**III**) was obtained as a pale-yellow solid (ca. 10 g).

### X-ray Photoelectron Spectroscopy

XPS spectra were recorded on a Kratos Axis Ultra spectrometer with a monochromated Al Kα source (*hv* = 1486.6 eV), a hybrid optical assembly (magnetic/electrostatic), a concentric hemispherical analyzer, a multichannel plate, and a delay line detector, with an X-ray beam incidence angle of 30° and a collection angle of 0° both relative to the surface normal. The X-ray source was operated at 10 mA emission current and 12 kV anode potential. All spectra were recorded using an entrance aperture of 300 μm × 700 μm with a pass energy of 80 eV for survey scans and 20 eV for high-resolution scans. The instrument sensitivity was 7.5 × 105 counts s^–1^ when measuring the Ag 3d_5/2_ photoemission peak for a clean Ag sample recorded at a pass energy of 20 eV and 450 W emission power. Ag 3d_5/2_ full width at half-maximum (fwhm) was 0.55 eV for the same instrument settings. Binding energy (BE) calibration was made using Au 4f_7/2_ (83.96 eV), Ag 3d_5/2_ (368.21 eV), and Cu 2p_3/2_ (932.62 eV). The absolute error in the acquisition of binding energies was ±0.1 eV, as quoted by the instrument manufacturer (Kratos). Therefore, any binding energies within 0.2 eV can be assumed as equivalent. Charge neutralization, when used, was applied employing a standard Kratos charge neutralizer consisting of a filament, coaxial with the electrostatic and magnetic transfer lenses, and a balance plate which creates a potential gradient between the neutralizer and sample. Charge neutralization was carried out at 1.9 A filament current and 3.3 V balance plate voltage. Sample stubs were earthed via the instrument stage using a standard BNC connector. The preparation method for each sample was dependent upon the nature of the material to be analyzed. Material samples were prepared by placing a small amount (ca. 10 mg) of the ionic liquid onto a stainless-steel multisampling bar; solid samples were fixed to the bar using a small piece of double-sided adhesive tape. Long exposure experiments were carried out by placing a small amount of the solid, or liquid sample, in a stainless steel or gold-coated stub. All samples were prepumped overnight to pressures lower than 1 × 10^–6^ mbar before being transferred into the main analytical chamber, where pressure of <1 × 10^–8^ mbar was maintained throughout the analysis. XP survey and high-resolution scans, with all expected photoelectron and Auger lines for each element, were recorded to demonstrate elemental composition and purity of the ILs. Samples were generally run at ambient temperatures (≈300 K) without charge neutralization and approximately 310 K when charge neutralization was required. Data was analyzed using the CASAXPS (Version 2.3.17 dev 6.6s) software.

### XPS Data Processing

Data was analyzed using the CASAXPS software (Version 2.3.17 dev 6.6s). Relative sensitivity factors (RSFs) were taken from the Kratos Library (RSF for F 1s = 1.0) and used to determine relative atomic percentages from high-resolution scans of the most intense photoelectron emission peak for each element. The fitting model for the 1,3-dialkylimidazolium cation was carried out using a previously described study,^69^ with adaptations due to the polymeric nature of the PS-BAIL. For PS-[(SO_3_H)^4^C_4_Im]­[OTf] (**III**); charge-referencing for all elements was achieved by setting the value of 284.8 eV for the polystyrene component (C_PS_).^67,68^ For model simplification, all the carbons of the polystyrene backbone (i.e., aliphatic and aromatic carbon of styrene units) were labeled as “C_PS_”. Peak areas were measured after performing a two-point linear or Tougaard background subtraction. GL(30) line shape (70% Gaussian/30% Lorentzian) was used for all photoemission peaks in high-resolution spectra. Area constraints were applied in the C 1s fitting model to account for an approximate 20% area loss in the C 1s components of the imidazolium cation due to shakeup and shake-off phenomena.^81,82^ Consequently, the relative peak area ratios for the cationic C 1s components were fixed at 0.8:1.6:2.0:3.0 for the C_2_, (C_4_ + C_5_), (C_6_ + C_7_), and (C_8_ + C_9_ + C_10_) peaks, respectively. Additionally, the fwhm values were set to be equal for the C_2_, (C_4_ + C_5_), (C_6_ + C_7_), and (C_8_ + C_9_ + C_10_) C 1s components, being constrained to a narrow range close to unity (0.8 ≤ fwhm ≤ 1.2 eV).^69^

### Thermogravimetric Analysis

TGA was performed on a TA Instruments Discovery SDT650—Simultaneous DSC–TGA Thermal Analyzer, using a 110 μL platinum plate, a sample mass of approximately 8 mg, a heating ramp of 10 °C min^–1^ from room temperature to 600 °C, and N_2_ atmosphere with a flow rate of 100 mL min^–1^. The TG curves were processed and analyzed using TRIOS (version 5.1) and OriginLab (version 8.5) software.

### Scanning Electron Microscopy

SEM analyses were performed on a Philips XL-30 FEG (Field Emission Gun) electron microscope, operating in high-vacuum mode with an accelerating voltage between 0.2 and 30 kV and a tungsten thermionic filament as the electron source. Samples were placed on a metallic stub using conductive carbon tape and subsequently coated with a thin layer of gold (≈10 nm) via sputtering to prevent surface charging during analysis.

### General Procedure for the GBB Reaction Catalyzed by PS-[(SO_3_H)^4^C_4_Im]­[OTf] (**III**)

In an appropriate microwave vial (Anton Paar or CEM manufacturer designs), a mixture of the 2-aminoazole (1.00 mmol), the aldehyde (1.00 mmol), and the isocyanide (1.00 mmol) was dissolved in absolute EtOH or MeOH (3 mL) in the presence of the heterogeneous catalyst PS-[(SO_3_H)^4^C_4_Im]­[OTf] (**III**) (50 mg mmol^–1^). The tube was sealed with a Teflon septum, and the reaction mixture was stirred (600 rpm) at 150 °C under microwave heating (variable power) for the time specified in Table 3 and Figures 6–8. Upon completion of the reaction, indicated by TLC analysis of the crude mixture (eluent: hexane/EtOAc 1:1, v/v), the suspension was filtered under vacuum, followed by washing the catalyst with MeOH (10 mL). The filtrate was concentrated on a rotary evaporator to yield the crude product, which was purified by column chromatography (eluent: 0–50% v/v EtOAc in hexane; gradient elution), affording the imidazo-heterocycles **1a–j**, **2a–h**, and **3a–f** in the yields described in Figures 6–8. The imidazo­[2,1-*b*]­thiazoles **2b**–**h** and the benzo­[*d*]­imidazo­[2,1-*b*]­thiazoles **3b**–**f** are described herein for the first time. Characterization data of all synthesized compounds are described in the Supporting Information.

### Reusability Test of the Catalyst PS-[(SO_3_H)^4^C_4_Im]­[OTf] (**III**)

In a G10 tube (Anton Paar model), a mixture of 2-aminopyridine (2.00 mmol, 0.188 g), benzaldehyde (2.00 mmol, 0.212 g, 204 μL), *tert*-butyl isocyanide (2.00 mmol, 0.166 g, 226 μL), and the catalyst (**III**) (100 mg) was dissolved in MeOH (6 mL). The tube was sealed with a Teflon septum, and the reaction mixture was stirred (600 rpm) at 150 °C under microwave heating (variable power) for 1 h. Upon completion of the reaction (indicated by TLC analysis), the heterogeneous catalyst was separated from the reaction mixture by vacuum filtration, followed by washing with MeOH and drying in a conventional oven under air at 60 °C for 1 h. The filtrate was concentrated under reduced pressure, and the crude product was purified by flash column chromatography (silica gel, eluent:hexane/EtOAc 1:1), affording **1a** in 91% yield. After weighing the dried catalyst, it was subjected to a subsequent GBB reaction run. Using this same recovery procedure, PS-[(SO_3_H)^4^C_4_Im]­[OTf] (**III**) could be reused for at least six consecutive cycles without significant loss of catalytic activity, leading to the formation of imidazo­[1,2-*a*]­pyridine **1a** in high yields (91%, 86%, 86%, 86%, 86%, and 80% for the six reaction cycles, respectively).

## Supplementary Material



## Notes

Characterization data of compounds **1a**–**j**, **2a**–**h**, and **3a**–**f** and 1H and 13C NMR spectra of compounds **1a**–**j**, **2a**–**h**, and **3a**–**f** (PDF)
