# Supplementary material for: Synthesis, Characterization, and Application of a Novel Polystyrene-Supported Brønsted-Acidic Ionic Liquid as an Efficient and Reusable Catalyst in Microwave-Assisted Groebke–Blackburn–Bienaymé Multicomponent Reaction
Source: ACS Omega. 2026 Jun 20;11(26):39204–17. doi: 10.1021/acsomega.6c03507 (PMC13347344; doi:10.1021/acsomega.6c03507)

Synthesis, Characterization and Application of a Novel Polystyrene-Supported Brønsted-Acidic Ionic Liquid as an Efficient and Reusable Catalyst in Microwave-Assisted Groebke-Blackburn-Bienaymé Multicomponent Reaction

Nicolas S. Anjos;[a] Daniel P. Marques;[a] Sandy J. Coutinho;[a] Fabiana S. F. Borges;[a] Ana Santos;[b] Peter Licence[b] and Luiz S. Longo Jr. [a]*

a) Nicolas S. Anjos; Fabiana S. F. Borges; Daniel P. Marques; Sandy J. Coutinho and Prof. Luiz S. Longo Jr.
Department of Pharmaceutical Sciences
Federal University of São Paulo - UNIFESP
Rua São Nicolau 210, 09913-030 Diadema, SP, Brazil
E-mail: luiz.longo@unifesp.br

b) Dr. Ana R. Santos; Prof. Peter Licence
School of Chemistry, GSK Carbon Neutral Laboratory
The University of Nottingham – Jubilee Campus
Nottingham, NG7 2GA, United Kingdom

SUPPORTING INFORMATION

1. Characterization data of 1a–j, 2a–h, and 3a–f

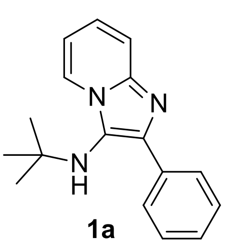

1a

***N*-(*tert*-butyl)-2-phenylimidazo[1,2-*a*]pyridin-3-amine** (**1a**); [CAS 214531-11-6].[1–3] A mixture of 2-aminopyridine (1.00 mmol; 0.094 g), benzaldehyde (1.00 mmol; 0.106 g; 102 μL), *tert*-butyl isocyanide (1.00 mmol; 0.083 g; 113 μL) and catalyst (**III**) (50 mg) in EtOH (3 or 6 mL) was conducted according to the methodology described in the Materials and Methods section. Product **1a** (0.910 mmol; 0.240 g; 91%) was obtained as a white solid; Mp = 157-159 °C (lit: 160-162 °C).[2] R_f = 0.32 (eluent: hexane/ethyl acetate 1:1 v/v). ^{1}H NMR (400 MHz, $CDCl_3$) δ 1.03 (s, 9H), 3.17 (br s, 1H), 6.76 (td, J = 6.7 and 0.9 Hz, 1H), 7.09-7.15 (m, 1H), 7.28-7.33 (m, 1H), 7.40-7.45 (m, 2H), 7.53-7.56 (m, 1H), 7.88-7.91 (m, 2H), 8.21-8.23 (m, 1H); ^{13}C NMR (100 MHz, $CDCl_3$) δ 30.4, 56.6, 111.4, 117.4, 123.6, 124.2, 127.5, 128.3, 128.4, 135.4, 139.6, 142.1.

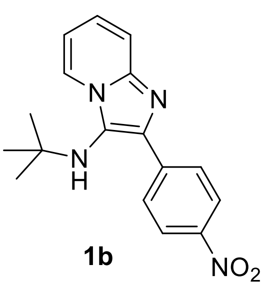

1b

***N*-(*tert*-butyl)-2-(4-nitrophenyl)imidazo[1,2-*a*]pyridin-3-amine** (**1b**); [CAS 2118265-54-4].[4] A mixture of 2-aminopyridine (1.00 mmol; 0.094 g), 4-nitrobenzaldehyde (1.00 mmol; 0.151 g), *tert*-butyl isocyanide (1.00 mmol; 0.083 g; 113 μL) and catalyst (**III**) (50 mg) in EtOH (6 mL) was conducted according to the methodology described in the Materials and Methods section. Product **1b** (0.850 mmol; 0.263 g; 85%) was obtained as an orange solid; Mp = 198-200 °C (lit: 198-200 °C).[4] R_f = 0.30 (eluent: hexane/ethyl acetate 1:1 v/v). ^{1}H NMR (400 MHz, $CDCl_3$) δ 1.09 (s, 9H), 3.02 (br s, 1H), 6.82 (td, J = 6.8 and 1.0 Hz, 1H), 7.17-7.21 (m, 1H), 7.54-7.56 (m, 1H), 8.17-8.29 (m, 5H); ^{13}C NMR (100 MHz, $CDCl_3$) δ 30.6, 57.0, 112.1, 117.9, 117.9, 123.7, 124.9, 125.0, 128.5, 137.3, 142.2, 142.7, 146.8.

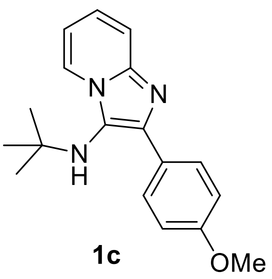

1c

***N*-(*tert*-butyl)-2-(4-methoxyphenyl)imidazo[1,2-*a*]pyridin-3-amine (1c);** [CAS 518015-55-9].[4] A mixture of 2-aminopyridine (1.00 mmol; 0.094 g), *p*-anisaldehyde (1.00 mmol; 0.136 g; 122 μL), *tert*-butyl isocyanide (1.00 mmol; 0.083 g; 113 μL) and catalyst (**III**) (50 mg) in EtOH (3 or 6 mL) was conducted according to the methodology described in the Materials and Methods section. Product **1c** (0.870 mmol; 0.257 g; 87%) was obtained as a yellowish solid; Mp = 130-132 °C (lit: 138-142 °C).[4] R_f = 0.20 (eluent: hexane/ethyl acetate 1:1 v/v) ^{1}H NMR (400 MHz, $CDCl_3$) δ 1.04 (s, 9H), 3.05 (br s,

1H), 3.85 (s, 3H), 6.75 (td, J = 6.8 and 0.8 Hz, 1H), 6.95-6.98 (m, 2H), 7.09-7.13 (m, 1H), 7.50-7.52 (m, 1H), 7.84-7.86 (m, 2H), 8.20-8.21 (m, 1H); ^{13}C NMR (100 MHz, $CDCl_3$) δ 30.5, 55.4, 56.5, 111.3, 113.8, 117.3, 123.0, 123.5, 123.9, 128.1, 129.5, 139.6, 142.1, 159.1.

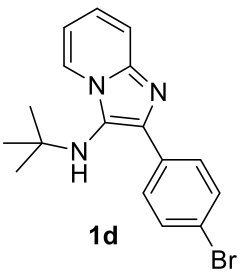

1d

2-(4-bromophenyl)-*N*-(*tert*-butyl)imidazo[1,2-*a*]pyridin-3-amine (1d); [CAS 1370642-49-1].[2,5] A mixture of 2-aminopyridine (1.00 mmol; 0.094 g), 4-bromobenzaldehyde (1.00 mmol; 0.175 g), *tert*-butyl isocyanide (1.00 mmol; 0.083 g; 113 μL) and catalyst (**III**) (50 mg) in EtOH (6 mL) was conducted according to the methodology described in the Materials and Methods section. Product **1d** (0.850 mmol; 0.293 g; 85%) was obtained as a white solid; Mp = 145-147 °C (lit: 146-147 °C).[2] R_f = 0.24 (eluent: hexane/ethyl acetate 1:1 v/v) ^{1}H NMR (300 MHz, $CDCl_3$) δ 1.03 (s, 9H), 2.99 (br s, 1H), 6.75 (t, J = 6.8 Hz, 1H), 7.09-7.14 (m, 1H), 7.50-7.54 (m, 3H), 7.83-7.86 (m, 2H), 8.16 (d, J = 6.8 Hz, 1H); ^{13}C NMR (75 MHz, $CDCl_3$) δ 30.5, 56.6, 111.6, 117.4, 121.4, 123.4, 123.6, 124.4, 129.7, 131.5, 134.4, 138.5, 142.2.

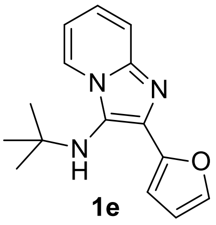

1e

***N*-(*tert*-butyl)-2-(furan-2-yl)imidazo[1,2-*a*]pyridin-3-amine (1e)** [CAS 943633-38-3] [4] A mixture of 2-aminopyridine (1.00 mmol; 0.094 g), furfural (1.00 mmol; 94 mg), *tert*-butyl isocyanide (1.00 mmol; 0.083 g; 113 μL) and catalyst (**III**) (50 mg) in EtOH (3 mL) was conducted according to the methodology described in the Materials and Methods section. Product **1e** (0.850 mmol; 0.217 g; 85%) was obtained as a white solid; Mp = 97-99 °C (lit: 98-101 °C).[4] R_f = 0.50 (eluent: hexane/ethyl acetate 1:1 v/v). ^{1}H NMR (300 MHz, $CDCl_3$) δ 1.13 (s, 9H), 3.51 (br s, 1H), 6.49-6.51 (m, 1H), 6.72 (td, J = 6.8 and 1.0 Hz, 1H), 6.87-6.88 (m, 1H), 7.06-7.12 (m, 1H), 7.45-7.48 (m, 2H), 8.20-8.23 (m, 1H); ^{13}C NMR (100 MHz, $CDCl_3$) δ 30.0, 56.4, 107.3, 111.3, 111.7, 117.2, 123.8, 124.3, 124.4, 130.8, 141.3, 142.5, 150.6.

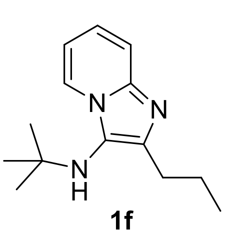

1f

***N*-(*tert*-butyl)-2-propylimidazo[1,2-*a*]pyridin-3-amine (1f);** [CAS 601468-08-0].[2,6] A mixture of 2-aminopyridine (1.00 mmol; 0.094 g), butyraldehyde (1.00 mmol; 0.072 g; 90 μL), *tert*-butyl isocyanide (1.00 mmol; 0.083 g; 113 μL) and catalyst (**III**) (50 mg) in EtOH (3 mL) was

conducted according to the methodology described in the Materials and Methods section. Product **1f** (0.880 mmol; 0.204 g; 88%) was obtained as a white solid; Mp = 132-133 °C (lit: 129-130 °C).[6] R_f = 0.16 (eluent: hexane/ethyl acetate 1:1 v/v). ^{1}H NMR (300 MHz, $CDCl_3$) δ 0.97 (t, J = 7.3 Hz, 3H), 1.19 (s, 9H), 1.73-1.86 (m, 2H), 2.70-2.75 (m, 2H), 6.71-6.81 (m, 1H), 7.14-7.20 (m, 2H), 7.68 (d, J = 9.0 Hz, 1H), 8.17-8.19 (m, 1H); ^{13}C NMR (75 MHz, $CDCl_3$) δ 14.4, 22.8, 30.0, 30.4, 55.6, 110.8, 116.8, 123.3, 123.9, 141.4, 142.0.

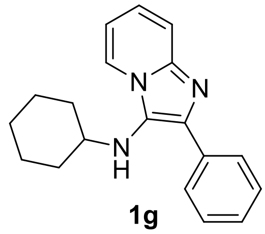

1g

***N*-cyclohexyl-2-phenylimidazo[1,2-*a*]pyridin-3-amin*e* (1g).**[4,7] [CAS 214531-48-3] A mixture of 2-aminopyridine (1.00 mmol; 0.094 g), benzaldehyde (1.00 mmol; 0.106 g; 102 μL), cyclohexyl isocyanide (1.00 mmol; 0.109 g; 124 μL) and catalyst (**III**) (50 mg) in EtOH (3.0 mL) was conducted according to the methodology described in the Materials and Methods section. Product **1g** (0.880 mmol; 0.255 g; 88%) was obtained as a white solid; Mp = 178-180 °C (lit: 177-179 °C).[7] R_f= 0.38 (eluent: hexane/ethyl acetate 1:1 v/v). ^{1}H NMR (400 MHz, $CDCl_3$) δ 1.13-1.25 (m, 5H), 1.68-184 (m, 5H), 2.94-3.12 (m, 2H), 6.78 (t, J = 6.7 Hz, 1H), 7.10-7.15 (m, 1H), 7.29-7.34 (m, 1H), 7.43-7.48 (m, 2H), 7.54 (d, J = 9.0 Hz, 1H), 8.02-8.05 (m, 2H), 8.11 (d, J = 6.7 Hz, 1H); ^{13}C NMR (100 MHz, $CDCl_3$) δ 25.0, 25.9, 34.3, 57.1, 111.7, 117.6, 122.9, 124.0, 125.1, 127.1, 127.4, 128.7, 134.7, 136.7, 141.7.

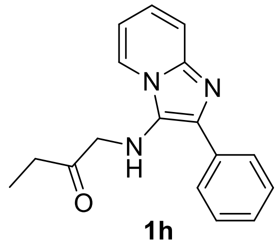

1h

1-((2-phenylimidazo[1,2-*a*]pyridin-3-yl)amino)butan-2-one (1h).[4] [CAS 497829-55-7] A mixture of 2-aminopyridine (1.00 mmol; 0.094 g), benzaldehyde (1.00 mmol; 0.106 g; 102 μL), ethyl isocyanoacetate (1.00 mmol; 0.113 g; 109 μL) and catalyst (**III**) (50 mg) in EtOH (3.0 mL) was conducted according to the methodology described in the Materials and Methods section. Product **1h** (0.840 mmol; 0.246 g; 84%) was obtained as a yellow oil; R_f= 0.44 (eluent: hexane/ethyl acetate 7:3 v/v). ^{1}H NMR (300 MHz, $CDCl_3$) δ 1.20 (t, J = 7.1 Hz, 3H), 3.77 (s, 3H), 4.15 (q, J = 7.1 Hz, 2H), 6.75 (t, J = 6.7 Hz, 1H), 7.07-7.13 (m, 1H), 7.26-7.53 (m, 4H), 8.01 (d, J = 7.8 Hz, 2H), 8.20 (d, J = 6.8 Hz, 1H); ^{13}C NMR (75 MHz, $CDCl_3$) δ 14.1, 49.4, 61.3, 111.8, 117.3, 122.8, 124.2, 124.8, 126.9, 127.5, 128.6, 134.0, 135.8, 141.6, 171.8.

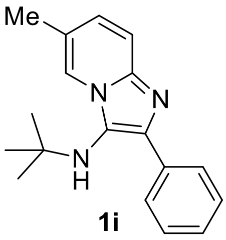

N-(*tert*-butyl)-6-methyl-2-phenylimidazo[1,2-*a*]pyridin-3-amine (1i); [CAS 334905-16-7].[4] A mixture of 2-amino-5-methylpyridine (1.00 mmol; 0.108 g), benzaldehyde (1.00 mmol; 0.106 g; 102 μL), *tert*-butyl isocyanide (1.00 mmol; 0.083 g; 113 μL) and catalyst (**III**) (50 mg) in EtOH (3 mL) was conducted according to the methodology described in the Materials and Methods section. Product **1i** (0.880 mmol; 0.245 g; 88%) was obtained as a white solid; Mp = 215-217 °C (lit: 216-219 °C).[4] R_f = 0.30 (eluent: hexane/ethyl acetate 1:1 v/v). ^{1}H NMR (300 MHz, $CDCl_3$) δ 1.04 (s, 9H), 2.34 (s, 3H), 6.98 (dd, J = 9.2 Hz and 1.5 Hz, 1H), 7.27-7.32 (m, 1H), 7.39-7.47 (m, 3H), 7.89-7.90 (m, 2H), 8.00 (s, 1H); ^{13}C NMR (75 MHz, $CDCl_3$) δ 18.6, 30.4, 56.5, 116.8, 121.0, 121.2, 123.3, 127.4, 128.2, 128.4, 135.5, 139.4, 141.2.

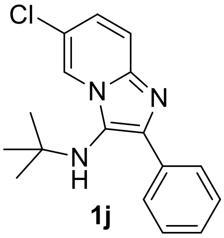

N-(*tert*-butyl)-6-chloro-2-phenylimidazo[1,2-*a*]pyridin-3-amine (1j); [CAS 214531-36-9][4] A mixture of 2-amino-5-chloropyridine (1.00 mmol; 0.129 g), benzaldehyde (1.00 mmol; 0.106 g; 102 μL), *tert*-butyl isocyanide (1.00 mmol; 0.083 g; 113 μL) and catalyst (**III**) (50 mg) in EtOH (3 mL) was conducted according to the methodology described in the Materials and Methods section. Product **1j** (0.740 mmol; 0.258 g; 86%) was obtained as a white solid. Mp = 208-211 °C (lit: 207-209 °C).[4] R_f = 0.52 (eluent: hexane/ethyl acetate 1:1 v/v). ^{1}H NMR (300 MHz, $CDCl_3$) δ 0.94 (s, 9H), 3.05 (br s, 1H), 6.99 (dd, J = 9.3 and 2.0 Hz, 1H), 7.19-7.40 (m, 4H), 7.78-7.79 (m, 2H), 8.14-8.15 (m, 1H); ^{13}C NMR (75 MHz, $CDCl_3$) δ 30.4, 56.6, 117.9, 119.9, 121.5, 124.1, 125.4, 127.8, 128.2, 128.4, 135.0, 140.4, 140.9.

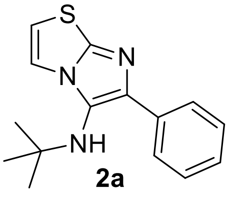

N-(*tert*-butyl)-6-phenylimidazo[2,1-*b*]thiazol-5-amine (2a); [CAS 214531-41-6].[4] A mixture of 2-aminothiazole (1.00 mmol; 0.100 g), benzaldehyde (1.00 mmol; 0.106 g; 102 μL), *tert*-butyl isocyanide (1.00 mmol; 0.083 g; 113 μL) and catalyst (**III**) (50 mg in EtOH (3 mL) was conducted according to the methodology described in the Materials and Methods section. Product **2a** (0.610 mmol; 0.166 g; 61%) was obtained as a yellowish solid; Mp = 150-152 °C (lit: 150-152 °C).[4] R_f = 0.24 (eluent: hexane/ethyl acetate 7:3 v/v). ^{1}H NMR (400 MHz, $CDCl_3$) δ 1.09 (s, 9H), 3.08 (br s, 1H), 6.75 (d, J = 4.5 Hz, 1H), 7.26-7.30 (m, 1H), 7.26-7.30 (m, 3H), 7.90-7.92 (m, 2H); ^{13}C NMR (100 MHz, $CDCl_3$) δ 30.3, 55.9, 111.5, 117.9, 125.7, 126.9, 127.3, 128.3, 135.4, 140.2, 145.6.

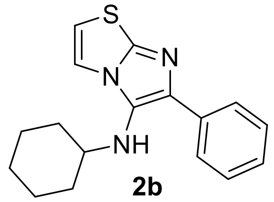
2b

***N*-cyclohexyl-6-phenylimidazo[2,1-*b*]thiazol-5-amine (2b).** [CAS 956350-74-6] [8] A mixture of 2-aminothiazole (1.00 mmol; 0.100 g), benzaldehyde (1.00 mmol; 0.106 g; 102 µL), cyclohexyl isocyanide (1.00 mmol; 0.109 g; 124 µL) and catalyst (**III**) (50 mg) in EtOH (3.0 mL) was conducted according to the methodology described in the Materials and Methods section. Product **2b** (0.610 mmol; 0.181 g; 61%) was obtained as a yellow solid; Mp = 129-131 °C (lit: 132-133 °C).[8] R_f = 0.24 (eluent: hexane/ethyl acetate 7:3 v/v). ^{1}H NMR (400 MHz, $CDCl_3$) δ 1.17-1.27 (m, 5H), 1.58-189 (m, 5H), 2.97-3.04 (m, 2H), 6.73 (d, J = 4.6 Hz, 1H), 7.23-7.27 (m, 1H), 7.32 (d, J = 4.8 Hz, 1H), 7.38-7.42 (m, 2H), 7.92-7.94 (m, 2H); ^{13}C NMR (100 MHz, $CDCl_3$) δ 24.9, 25.8, 34.4, 57.6, 111.8, 117.2, 126.2, 126.7, 127.4, 128.6, 134.9, 137.3, 145.0.

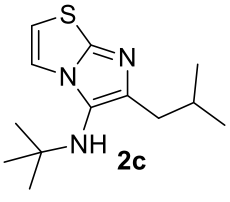
2c

***N*-(*tert*-butyl)-6-isobutylimidazo[2,1-*b*]thiazol-5-amine (2c).** A mixture of 2-aminothiazole (1.00 mmol; 0.100 g), isovaleraldehyde (1.00 mmol; 0.086 g; 108 µL), *tert*-butyl isocyanide (1.00 mmol; 0.083 g; 113 µL) and catalyst (**III**) (50 mg) in EtOH (3.0 mL) was conducted according to the methodology described in the Materials and Methods section. Product **2c** (0.750 mmol; 0.188 g; 75%) was obtained as a yellowish solid; Mp = 129-131 °C; R_f = 0.20 (eluent: hexane/ethyl acetate 7:3 v/v). ^{1}H NMR (400 MHz, $CDCl_3$) δ 0.92 (d, J = 6.6 Hz, 6H), 1.16 (s, 9H), 2.13 (nonet, J = 6.7 Hz, 1H), 2.44 (d, J = 6.7 Hz, 1H), 2.68 (br s, 1H), 6.64 (d, J = 4.5 Hz, 1H), 7.29 (d, J = 4.5 Hz, 1H); ^{13}C NMR (100 MHz, $CDCl_3$) δ 22.8, 28.8, 30.4, 37.1, 55.0, 110.3, 117.9, 126.0, 141.0, 144.8. HRMS ESI(+) *m/z*, calcd. for $C_{13}H_{22}N_3S^+$ $[M+H]^+$: 252.1526, found: 252.1529.

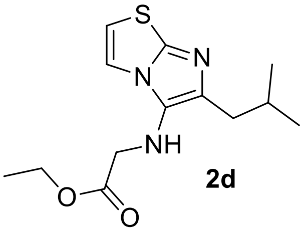
2d

Ethyl 2-((6-isobutylimidazo[2,1-*b*]thiazol-5-yl)amino)acetate (2d). A mixture of 2-aminothiazole (1.00 mmol; 0.100 g), isovaleraldehyde (1.00 mmol; 0.086 g; 108 µL), ethyl isocyanoacetate (1.00 mmol; 0.113 g; 109 µL) and catalyst (**III**) (50 mg) in EtOH (3.0 mL) was conducted according to the methodology described in the Materials and Methods section. Product **2d** (0.710 mmol; 0.200 g; 71%) was obtained as a yellowish oil; R_f = 0.28 (eluent: hexane/ethyl acetate 7:3 v/v). ^{1}H NMR (400 MHz, $CDCl_3$) δ 0.92 (d, J = 6.6 Hz, 6H), 1.25 (t, J = 7.1 Hz, 3H), 2.04 (nonet, J = 6.6 Hz, 1H), 2.43 (d, J = 6.7 Hz, 1H), 3.35 (br s, 1H), 3.70 (d, J = 5.6 Hz, 2H), 4.18 (q, J = 5.6 Hz,

1H), 6.66 (d, J = 4.5 Hz, 1H), 7.41 (d, J = 4.5 Hz, 1H); ^{13}C NMR (100 MHz, $CDCl_3$) δ 14.3, 22.7, 28.8, 37.1, 51.2, 61.4, 110.8, 117.4, 127.7, 139.4, 144.6, 172.4; HRMS ESI(+) *m/z*, calcd. for $C_{13}H_{20}N_3O_2S^+$ $[M+H]^+$: 282.1271 found: 282.1276.

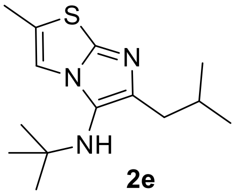
2e

***N*-(*tert*-butyl)-6-isobutyl-2-methylimidazo[2,1-*b*]thiazol-5-amine (2e).** A mixture of 2-amino-5-methylthiazole (1.00 mmol; 0.114 g), isovaleraldehyde (1.00 mmol; 0.086 g; 108 μL), *tert*-butyl isocyanide (1.00 mmol; 0.083 g; 113 μL) and catalyst (**III**) (50 mg) in EtOH (3.0 mL) was conducted according to the methodology described in the Materials and Methods section. Product **2e** (0.750 mmol; 0.199 g; 75%) was obtained as a yellow solid; Mp = 95-97 °C; R_f= 0.28 (eluent: hexane/ethyl acetate 7:3 v/v). ^{1}H NMR (400 MHz, $CDCl_3$) δ 0.91 (d, J = 6.6 Hz, 6H), 1.15 (s, 9H), 2.08-2.18 (m, 1H), 2.35 (d, J = 1.4 Hz, 3H), 2.40-2.42 (m, 2H), 2.62 (br s, 1H), 6.99 (d, J = 1.4 Hz, 1H); ^{13}C NMR (100 MHz, $CDCl_3$) δ 14.3, 22.8, 28.8, 30.4, 37.1, 54.9, 114.6, 124.1, 125.7, 139.5, 144.2; HRMS ESI(+) *m/z*, calcd. for $C_{14}H_{24}N_3S^+$ $[M+H]^+$: 266.1685, found: 266.1681.

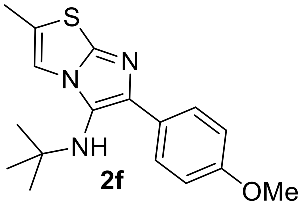
2f

***N*-(*tert*-butyl)-6-(4-methoxyphenyl)-2-methylimidazo[2,1-*b*]thiazol-5-amine (2f).** A mixture of 2-amino-5-methylthiazole (1.00 mmol; 0.114 g), *p*-anisaldehyde (1.00 mmol; 0.136 g; 122 μL), *tert*-butyl isocyanide (1.00 mmol; 0.083 g; 113 μL) and catalyst (**III**) (50 mg) in EtOH (3.0 mL) was conducted according to the methodology described in the Materials and Methods section. Product **2f** (0.560 mmol; 0.177 g; 56%) was obtained as a yellow solid; Mp = 100-102 °C; R_f= 0.28 (eluent: hexane/ethyl acetate 7:3 v/v). ^{1}H NMR (400 MHz, $CDCl_3$) δ 1.05 (s, 9H), 2.38 (d, J = 1.2 Hz, 3H), 2.92 (br s, 1H), 3.82 (s, 3H), 6.90 (d, J = 8.8 Hz, 2H), 7.04 (d, J = 1.2 Hz, 1H), 7.80 (d, J = 8.8 Hz, 2H); ^{13}C NMR (100 MHz, $CDCl_3$) δ 14.3, 30.4, 55.3, 55.7, 113.7, 114.5, 124.6, 125.1, 128.3, 128.4, 138.8, 144.9, 158.4; HRMS ESI(+) *m/z*, calcd. for $C_{17}H_{22}N_3OS^+$ $[M+H]^+$: 316.1478, found: 316.1483.

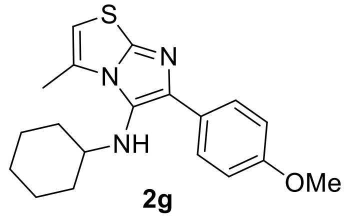
2g

***N*-cyclohexyl-6-(4-methoxyphenyl)-3-methylimidazo[2,1-*b*]thiazol-5-amine (2g).** A mixture of 2-amino-4-methylthiazole (1.00 mmol; 0.114 g), *p*-anisaldehyde (1.00 mmol; 0.136 g; 122

μL), cyclohexyl isocyanide (1.00 mmol; 0.109 g; 124 μL) and catalyst (**III**) (50 mg in EtOH (3.0 mL) was conducted according to the methodology described in the Materials and Methods section. Product **2g** (0.340 mmol; 0.116 g; 34%) was obtained as a yellow solid; Mp = 102-104 °C; Rf = 0.30 (eluent: hexane/ethyl acetate 7:3 v/v). ^{1}H NMR (400 MHz, $CDCl_3$) δ 1.09-1.11 (m, 5H), 1.55-1.78 (m, 5H), 2.55 (d, *J* = 1.0 Hz, 3H), 2.79-2.85 (m, 2H), 3.83 (s, 3H), 6.20 (d, *J* = 1.0 Hz, 1H), 6.92 (d, *J* = 8.8 Hz, 2H), 7.81 (d, *J* = 8.8 Hz, 2H); ^{13}C NMR (100 MHz, $CDCl_3$) δ 14.3, 25.1, 25.9, 33.7, 55.3, 58.6, 106.2, 113.8, 127.5, 128.0, 128.0, 129.6, 138.8, 146.2, 158.5; HRMS ESI(+) *m/z*, calcd. for $C_{19}H_{24}N_3O_1S^+$ $[M+H]^+$: 342.1635, found: 342.1630.

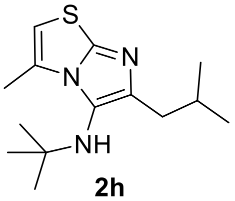

2h

***N*-(*tert*-butyl)-6-isobutyl-3-methylimidazo[2,1-*b*]thiazol-5-amine (2h).** A mixture of 2-amino-4-methylthiazole (1.00 mmol; 0.114 g), isovaleraldehyde (1.00 mmol; 0.086 g; 108 μL), *tert*-butyl isocyanide (1.00 mmol; 0.083 g; 113 μL) and catalyst (**III**) (50 mg) in EtOH (3.0 mL) was conducted according to the methodology described in the Materials and Methods section. Product **2h** (0.550 mmol; 0.146 g; 55%) was obtained as a yellow solid; Mp = 108-110 °C; R_f = 0.40 (eluent: hexane/ethyl acetate 7:3 v/v). ^{1}H NMR (400 MHz, $CDCl_3$) δ 0.92 (d, *J* = 6.6 Hz, 6H), 1.15 (s, 9H), 2.08-2.18 (m, 1H), 2.41 (br s, 1H), 2.48 (d, *J* = 7.2 Hz, 1H), 2.57 (d, *J* = 1.1 Hz, 3H), 6.17 (d, *J* = 1.1 Hz, 1H); ^{13}C NMR (100 MHz, $CDCl_3$) δ 14.8, 22.8, 28.8, 30.5, 37.6, 54.7, 105.5, 127.6, 130.0, 141.8, 146.1; HRMS ESI(+) *m/z*, calcd. for $C_{14}H_{24}N_3S^+$ $[M+H]^+$: 266.1685, found: 266.1680.

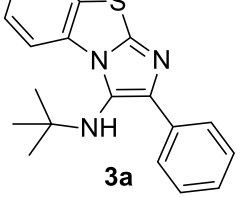

3a

***N*-(*tert*-butyl)-2-phenylbenzo[*d*]imidazo[2,1-*b*]thiazol-3-amine (3a).** [CAS 1126640-07-0][8]. A mixture of 2-aminobenzothiazole (1.00 mmol; 0.150 g), benzaldehyde (1.00 mmol; 0.106 g; 102 μL), *tert*-butyl isocyanide (1.00 mmol; 0.083 g; 113 μL) and catalyst (**III**) (50 mg) in EtOH (3.0 mL) was conducted according to the methodology described in the Materials and Methods section. Product **3a** (0.740 mmol; 0.238 g; 74%) was obtained as a white solid; Mp = 161-163 °C; R_f = 0.54 (eluent: hexane/ethyl acetate 7:3 v/v). ^{1}H NMR (400 MHz, $CDCl_3$) δ 1.04 (s, 9H), 3.21 (br s, 1H), 7.27-7.32 (m, 2H), 7.39-7.43 (m, 3H), 7.64-7.66 (m, 1H), 7.75-7.77 (m, 2H), 8.34-8.36 (m, 1H); ^{13}C NMR (100 MHz, $CDCl_3$) δ 30.4, 56.7, 114.7, 124.1, 124.3, 125.5, 127.2, 127.9, 128.4, 130.5, 133.9, 135.3, 141.3, 144.1.

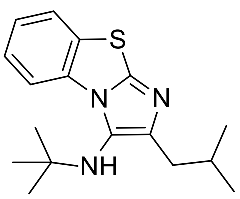

3b

***N*-(*tert*-butyl)-2-isobutylbenzo[*d*]imidazo[2,1-*b*]thiazol-3-amine (3b).** A mixture of 2-aminobenzothiazole (1.00 mmol; 0.150 g), isovaleraldehyde (1.00 mmol; 0.086 g; 108 µL), *tert*-butyl isocyanide (1.00 mmol; 0.083 g; 113 µL) and catalyst (**III**) (50 mg) in EtOH (3.0 mL) was conducted according to the methodology described in the Materials and Methods section. Product **3b** (0.830 mmol; 0.250 g; 83%) was obtained as a white solid; Mp = 95-97 °C; R_f = 0.52 (eluent: hexane/ethyl acetate 7:3 v/v). ^{1}H NMR (400 MHz, $CDCl_3$) δ 0.94 (d, J = 6.6 Hz, 6H), 1.22 (s, 9H), 2.15 (nonet, J = 6.6 Hz, 1H), 2.50-2.52 (m, 2H), 2.68 (br s, 1H), 7.22-7.26 (m, 1H), 7.34-7.38 (m, 1H), 7.60-7.62 (m, 1H), 8.25-8.27 (m, 1H); ^{13}C NMR (75 MHz, $CDCl_3$) δ 22.8, 28.8, 30.3, 37.3, 56.0, 114.4, 123.8, 124.0, 125.2, 128.8, 130.3, 133.9, 141.4, 143.3; HRMS ESI(+) *m/z*, calcd. for $C_{17}H_{24}N_3S^+$ [M+H]$^+$: 302.1685, found: 302.1687.

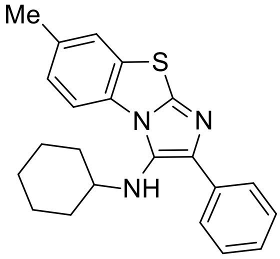

3c

***N*-cyclohexyl-7-methyl-2-phenylbenzo[*d*]imidazo[2,1-*b*]thiazol-3-amine (3c).** A mixture of 2-amino-6-methylbenzothiazole (1.00 mmol; 0.164 g), benzaldehyde (1.00 mmol; 0.106 g; 102 µL), cyclohexyl isocyanide (1.00 mmol; 0.109 g; 124 µL) and catalyst (**III**) (50 mg) in EtOH (3.0 mL) was conducted according to the methodology described in the Materials and Methods section. Product **3c** (0.610 mmol; 0.220 g; 61%) was obtained as a yellow solid; Mp = 122-125 °C; R_f= 0.46 (eluent: hexane/ethyl acetate 8:2 v/v). ^{1}H NMR (300 MHz, $CDCl_3$) δ 1.13-1.23 (m, 5H), 1.56-1.90 (m, 5H), 2.44 (s, 3H), 2.95 (br s, 1H), 3.18 (d, J = 3.8 Hz, 1H), 7.19-7.28 (m, 2H), 7.38-7.44 (m, 3H), 7.88-7.93 (m, 3H); ^{13}C NMR (75 MHz, $CDCl_3$) δ 21.4, 25.0, 26.0, 33.8, 57.7, 113.5, 124.2, 126.5, 126.7, 126.9, 128.6, 129.5, 130.5 131.3, 134.4, 134.8, 137.8, 143.3. HRMS ESI(+) *m/z*, calcd. for $C_{22}H_{24}N_3S^+$ [M+H]$^+$: 362.1665, found: 362.1693.

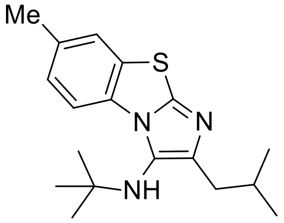

3d

***N*-(*tert*-butyl)-2-isobutyl-7-methylbenzo[*d*]imidazo[2,1-*b*]thiazol-3-amine (3d).** A mixture of 2-amino-6-methylbenzothiazole (1.00 mmol; 0.164 g), isovaleraldehyde (1.00 mmol; 0.086 g; 108 µL), *tert*-butyl isocyanide (1.00 mmol; 0.083 g; 113 µL) and catalyst (**III**) (50 mg) in EtOH (3.0 mL) was conducted according to the methodology described in the Materials and Methods section. Product **3d** (0.660 mmol; 0.208 g; 66%) was obtained as a yellow solid; Mp = 126-128 °C; R_f= 0.52 (eluent: hexane/ethyl acetate 7:3 v/v). ^{1}H NMR (400 MHz, $CDCl_3$) δ 0.94 (d, J = 6.6 Hz, 6H), 1.21 (s, 9H), 2.15 (nonet, J = 6.6 Hz,

1H), 2.43 (s, 3H), 2.49-2.51 (m, 2H), 2.70 (br s, 1H), 7.15-7.17 (m, 1H), 7.41 (s, 1H), 8.11 (m, 1H); ^{13}C NMR (100 MHz, $CDCl_3$) δ 21.4, 22.8, 28.8, 30.3, 37.3, 55.6, 114.0, 124.0, 126.2, 128.6, 130.4, 131.9, 133.7, 141.1, 143.1; HRMS ESI(+) *m/z*, calcd. for $C_{18}H_{26}N_3S^+$ $[M+H]^+$: 316.1842, found: 316.1850.

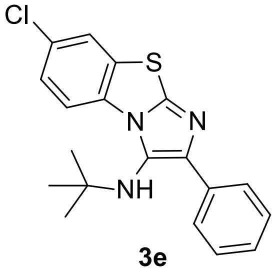

3e

***N*-(*tert*-butyl)-7-chloro-2-phenylbenzo[*d*]imidazo[2,1-*b*]thiazol-3-amine (3e).** A mixture of 2-amino-6-chlorobenzothiazole (1.00 mmol; 0.184 g), benzaldehyde (1.00 mmol; 0.106 g; 102 µL), *tert*-butyl isocyanide (1.00 mmol; 0.083 g; 113 µL) and catalyst (**III**) (50 mg) in EtOH (3.0 mL) was conducted according to the methodology described in the Materials and Methods section. Product **3e** (0.730 mmol; 0.260 g; 73%) was obtained as a yellow solid; Mp = 164-167 °C; R_f= 0.54 (eluent: hexane/ethyl acetate 7:3 v/v). ^{1}H NMR (300 MHz, $CDCl_3$) δ 1.02 (s, 9H), 3.20 (br s, 1H), 7.28-7.42 (m, 4H), 7.62 (s, 1H), 7.71-7.74 (m, 2H), 8.25-8.28 (m, 1H); ^{13}C NMR (75 MHz, $CDCl_3$) δ 30.0, 56.8, 115.3, 123.7, 125.9, 127.4, 127.9, 128.5, 129.8, 132.0, 132.4, 135.1, 141.6, 143.7; HRMS ESI(+) *m/z*, calcd. for $C_{19}H_{19}ClN_3S^+$ $[M+H]^+$: 356.0983, found: 356.0989.

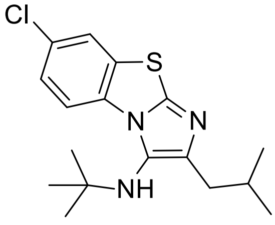

3f

***N*-(*tert*-butyl)-7-chloro-2-isobutylbenzo[*d*]imidazo[2,1-*b*]thiazol-3-amine (3f).** A mixture of 2-amino-6-chlorobenzothiazole (1.00 mmol; 0.184 g), isovaleraldehyde (1.00 mmol; 0.086 g; 108 µL), *tert*-butyl isocyanide (1.00 mmol; 0.083 g; 113 µL) and catalyst (**III**) (50 mg) in EtOH (3.0 mL) was conducted according to the methodology described in the Materials and Methods section. Product **3f** (0.680 mmol; 0.228 g; 68%) was obtained as a yellow solid; Mp = 135-137 °C; R_f= 0.50 (eluent: hexane/ethyl acetate 7:3 v/v). ^{1}H NMR (400 MHz, $CDCl_3$) δ 0.94 (d, J = 6.6 Hz, 6H), 1.21 (s, 9H), 2.14 (nonet, J = 6.7 Hz, 1H), 2.49-2.51 (m, 2H), 2.66 (br s, 1H), 7.33 (dd, J = 8.7 Hz and 2.0 Hz, 1H), 7.60 (d, J = 2.0 Hz, 1H), 8.19 (d, J = 8.7 Hz, 1H); ^{13}C NMR (100 MHz, $CDCl_3$) δ 22.8, 28.8, 30.3, 37.3, 55.7, 115.0, 123.6, 125.7, 128.9, 129.2, 131.8, 132.4, 141.8, 143.0; HRMS ESI(+) *m/z*, calcd. for $C_{17}H_{23}ClN_3S^+$ $[M+H]^+$: 336.1296, found: 336.1300.

2. References

(1) Baviskar, A. T.; Madaan, C.; Preet, R.; Mohapatra, P.; Jain, V.; Agarwal, A.; Guchhait, S. K.; Kundu, C. N.; Banerjee, U. C.; Bharatam, P. V. N-Fused Imidazoles As Novel Anticancer Agents That Inhibit Catalytic Activity of Topoisomerase II r and Induce Apoptosis in G1/S Phase. *J. Med. Chem.* **2011**, *54*, 5013–5030. https://doi.org/dx.doi.org/10.1021/jm200235u.

(2) Allahabadi, E.; Ebrahimi, S.; Soheilizad, M.; Khoshneviszadeh, M. Copper-Catalyzed Four-Component Synthesis of Imidazo[1,2-a]Pyridines via Sequential Reductive Amination , Condensation , and Cyclization. *Tetrahedron Lett.* **2017**, *58* (2), 121–124. https://doi.org/10.1016/j.tetlet.2016.11.081.

(3) Vidyacharan, S.; Shinde, A. H.; Satpathi, B.; Sharada, D. S. A Facile Protocol for the Synthesis of 3-Aminoimidazo-Fused Heterocycles via the Groebke–Blackburn–Bienayme Reaction under Catalyst-Free and Solvent-Free Conditions. *Green Chem.* **2014**, *16*, 1168–1175. https://doi.org/10.1039/c3gc42130a.

(4) Anjos, N. S.; Chapina, A. I.; Santos, A. R.; Licence, P.; Longo, L. S. Groebke-Blackburn-Bienaymé Multicomponent Reaction Catalysed by Reusable Brønsted-Acidic Ionic Liquids. *Eur. J. Org. Chem.* **2022**, No. 40, e202200615. https://doi.org/10.1002/ejoc.202200615.

(5) Khan, A. T.; Basha, R. S.; Lal, M. Bromodimethylsulfonium Bromide (BDMS) Catalyzed Synthesis of Imidazo [1,2- a] Pyridine Derivatives and Their Fluorescence Properties. *Tetrahedron Lett.* **2012**, *53* (17), 2211–2217. https://doi.org/10.1016/j.tetlet.2012.02.078.

(6) Guchhait, S. K.; Madaan, C. An Efficient, Regioselective, Versatile Synthesis of N-Fused 2- and 3-Aminoimidazoles via Ugi-Type Multicomponent Reaction Mediated by Zirconium(IV) Chloride in Polyethylene Glycol-400. *Synlett* **2009**, No. 4, 628–632. https://doi.org/10.1055/s-0028-1087915.

(7) Santos, G. F. D.; Anjos, N. S.; Gibeli, M. M.; Silva, G. A.; Fernandes, P. C. S.; Fiorentino, E. S. C.; Longo, L. S. A Comparative Study on the Groebke-Blackburn-Bienaymé Three-Component Reaction Catalyzed by Rare Earth Triflates under Microwave Heating. *J. Braz. Chem. Soc.* **2020**, *31* (7), 1434–1444. https://doi.org/10.21577/0103-5053.20200028.

(8) Mathavan, S.; B. R. D. Yamajala, R. Sustainable Synthetic Approaches for 3-Aminoimidazo-Fused Heterocycles via Groebke-Blackburn-Bienaymé Process. *ChemistrySelect* **2020**, *5* (34), 10637–10642. https://doi.org/10.1002/slct.202002894.

Figure S1. ^{1}H NMR spectra of **1a** (300 MHz, $CDCl_3$, δ).

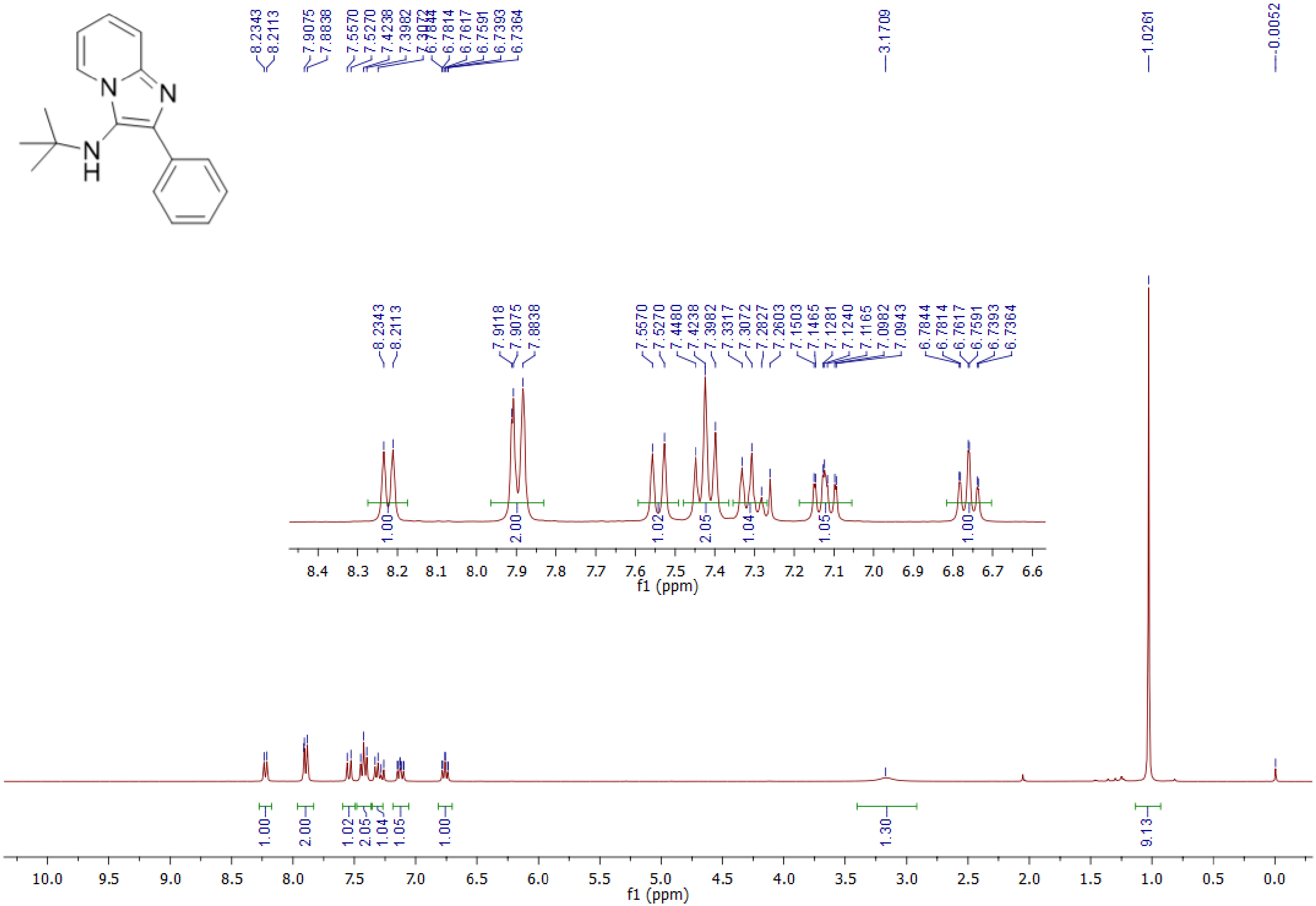

Figure S2. ^{13}C NMR spectra of **1a** (75 MHz, CDCl$_3$, δ).

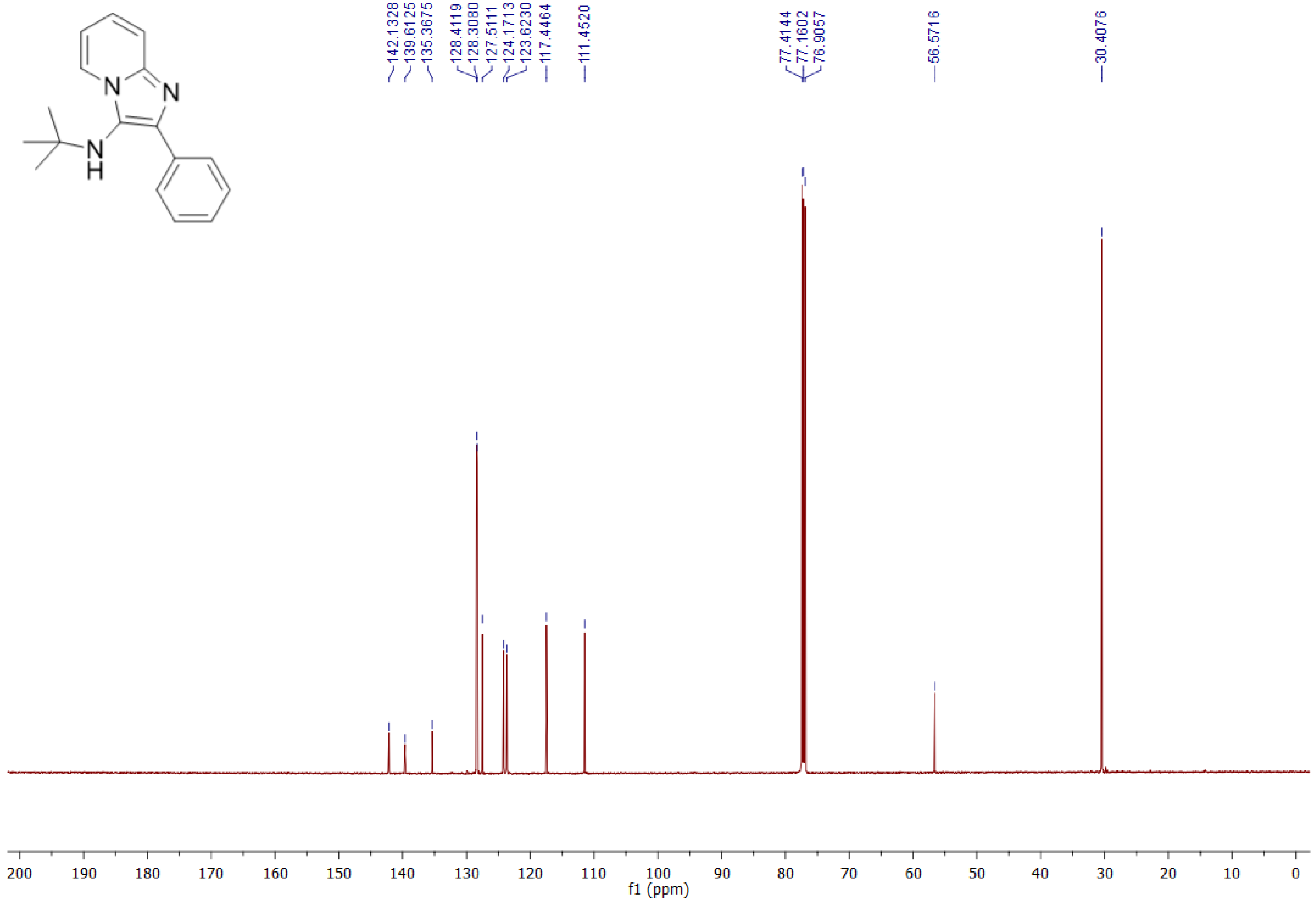

Figure S3. ^{1}H NMR spectra of **1b** (300 MHz, $CDCl_3$, δ).

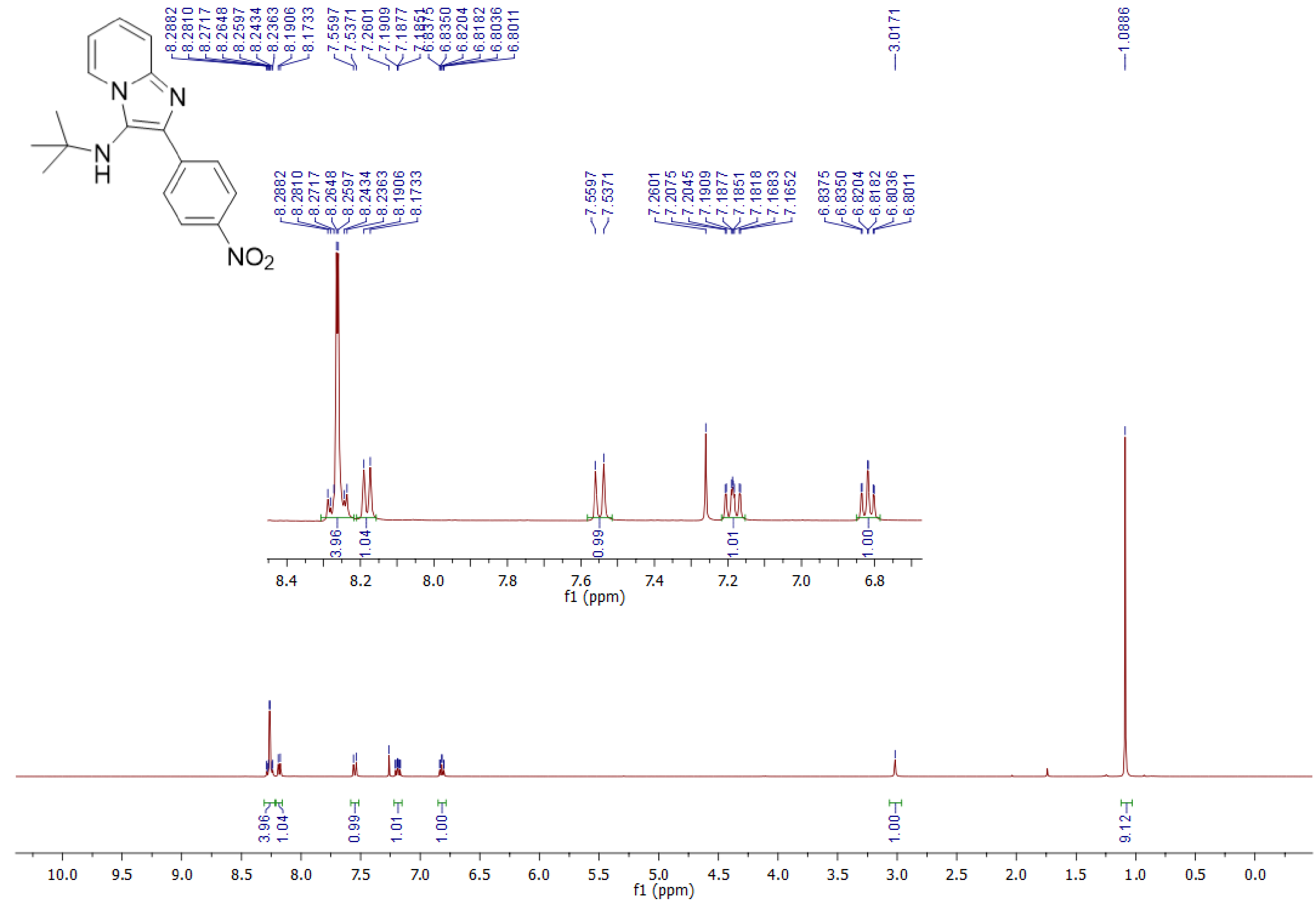

Figure S4. ^{13}C NMR spectra of **1b** (75 MHz, $CDCl_3$, δ).

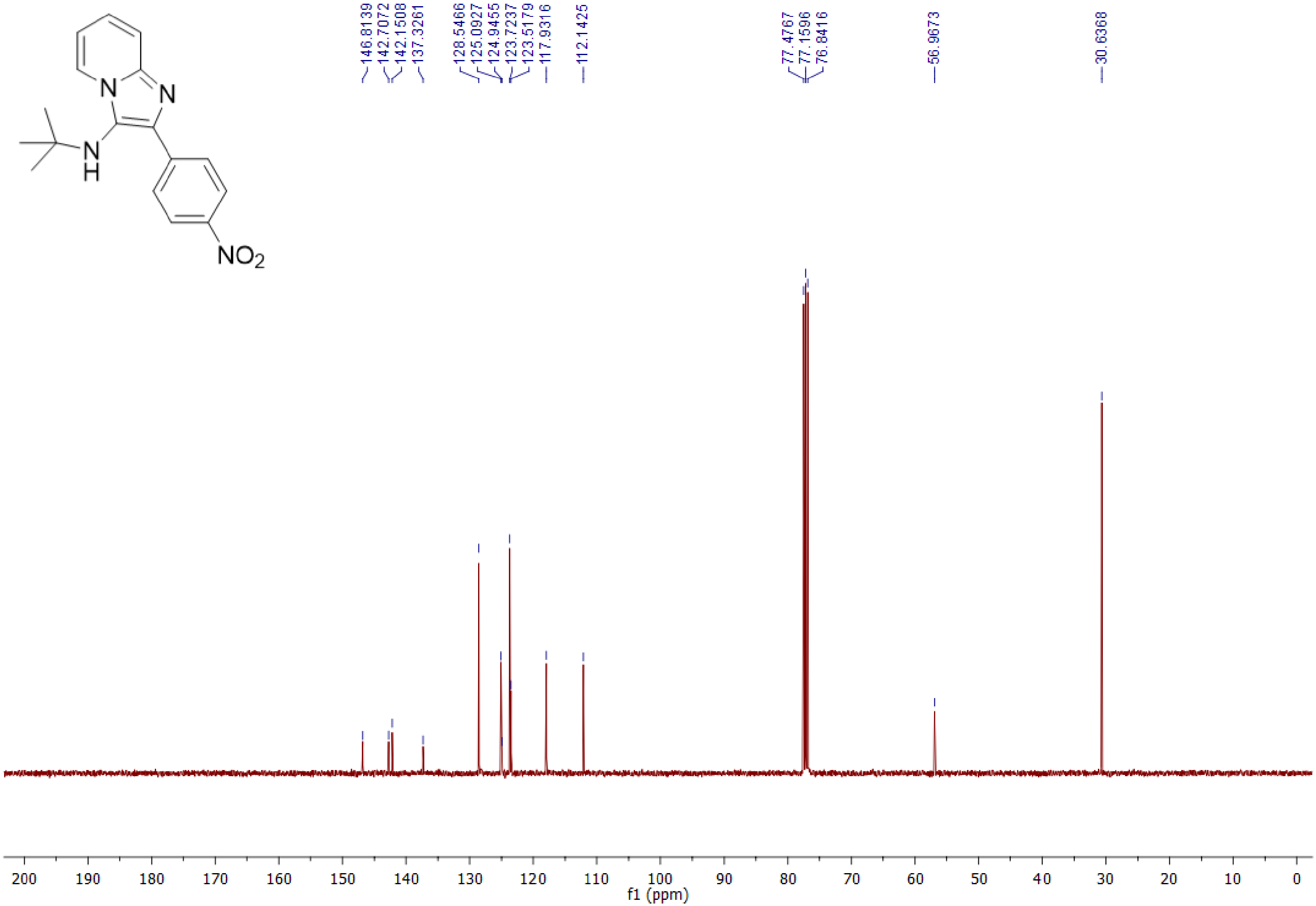

Figure S5. ^{1}H NMR spectra of **1c** (300 MHz, $CDCl_3$, δ).

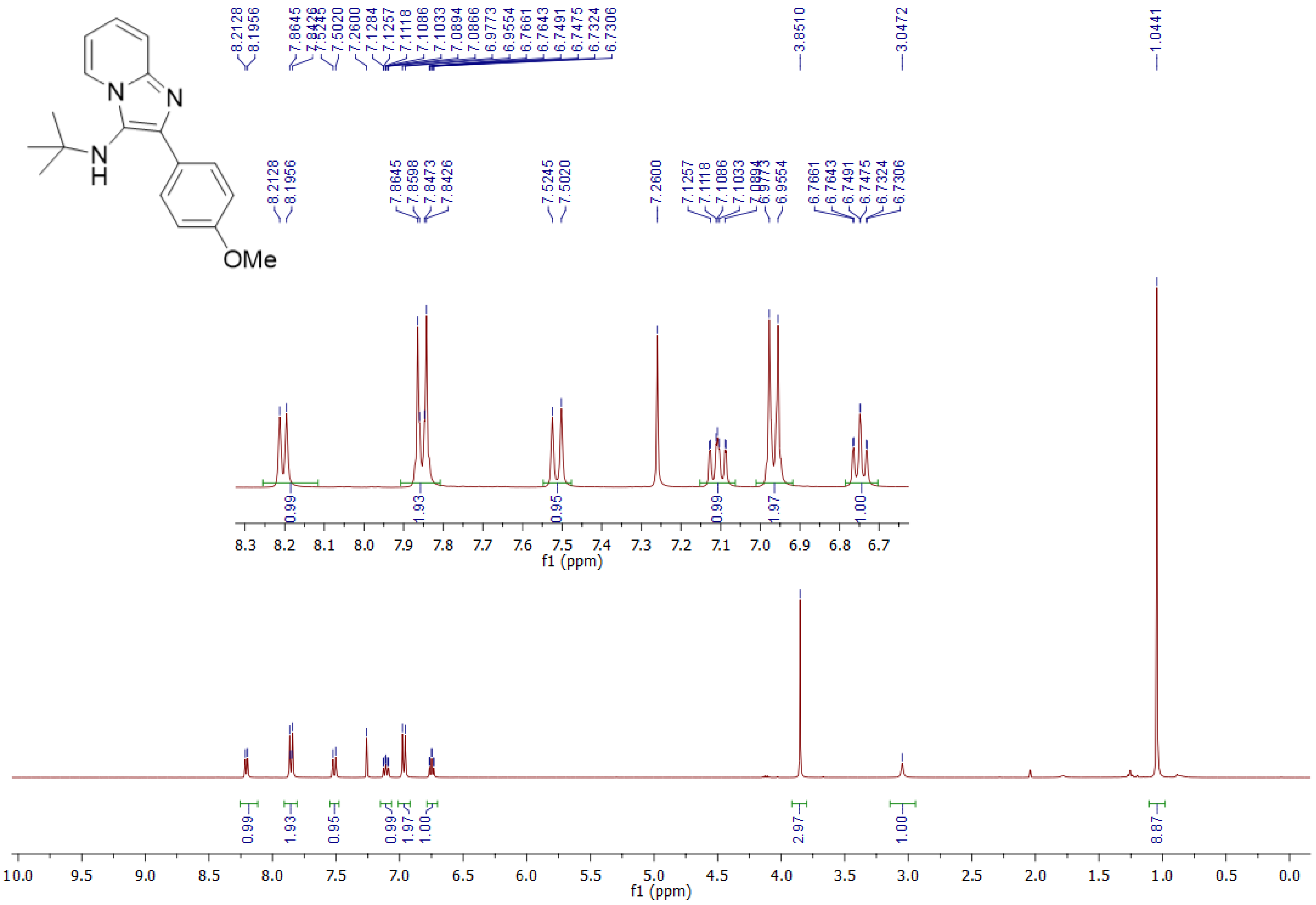

Figure S6. ^{13}C NMR spectra of **1c** (75 MHz, $CDCl_3$, δ).

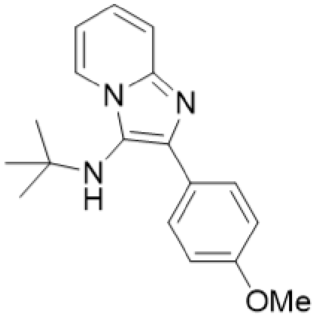

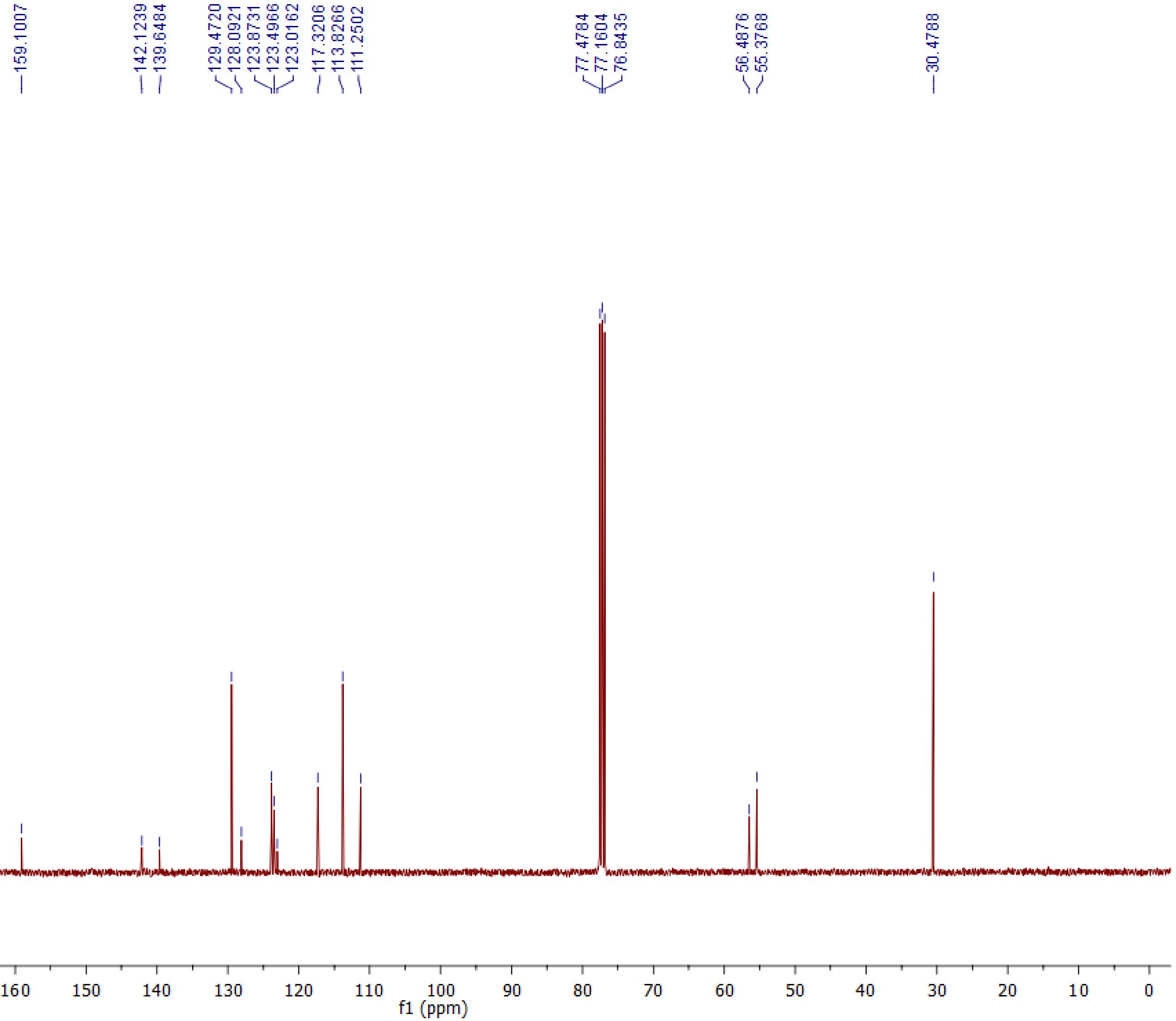

Figure S7. ^{1}H NMR spectra of **1d** (300 MHz, $CDCl_3$, δ).

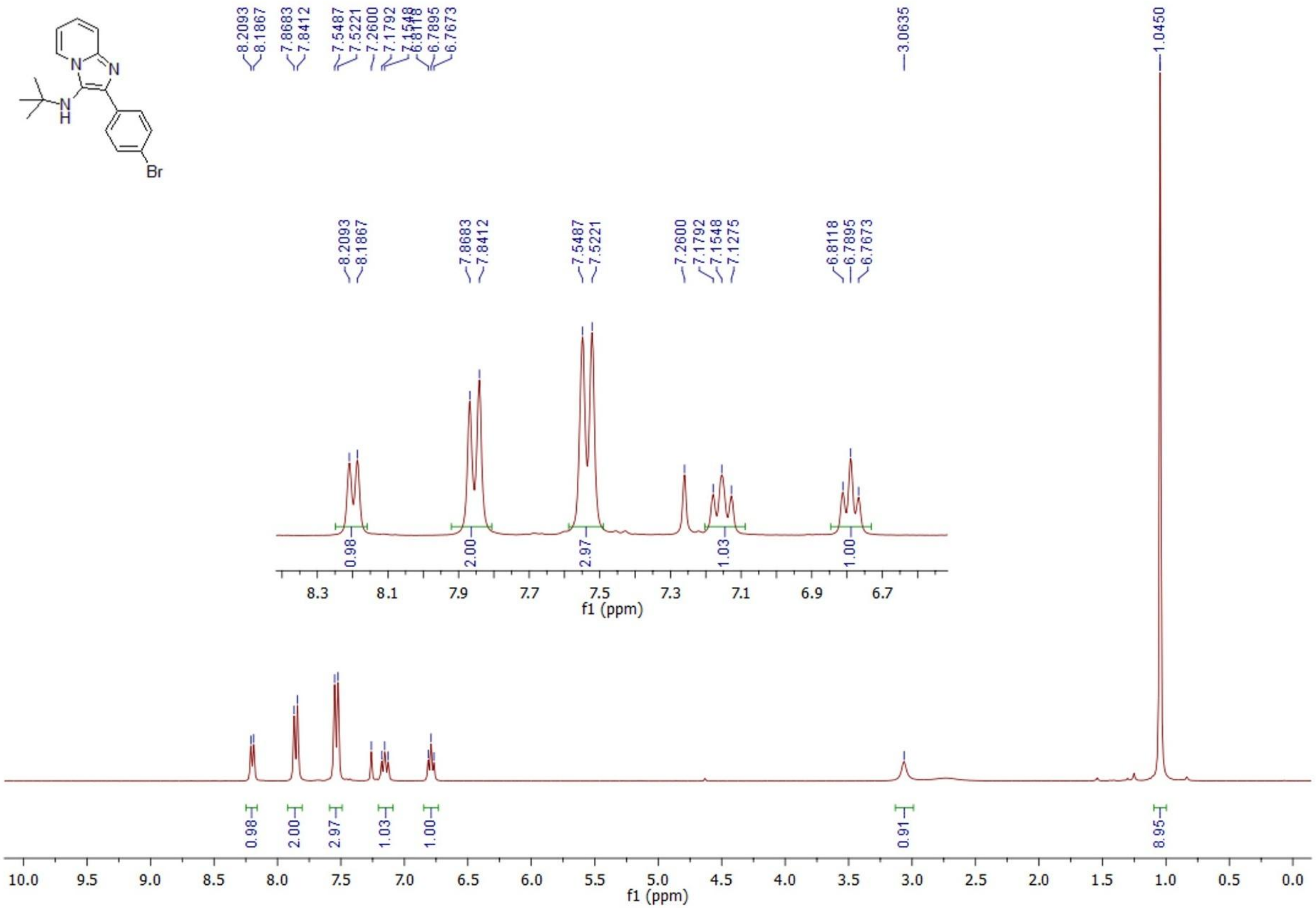

Figure S8. ^{13}C NMR spectra of **1d** (75 MHz, $CDCl_3$, δ).

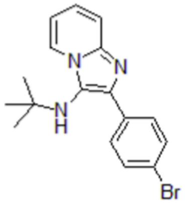

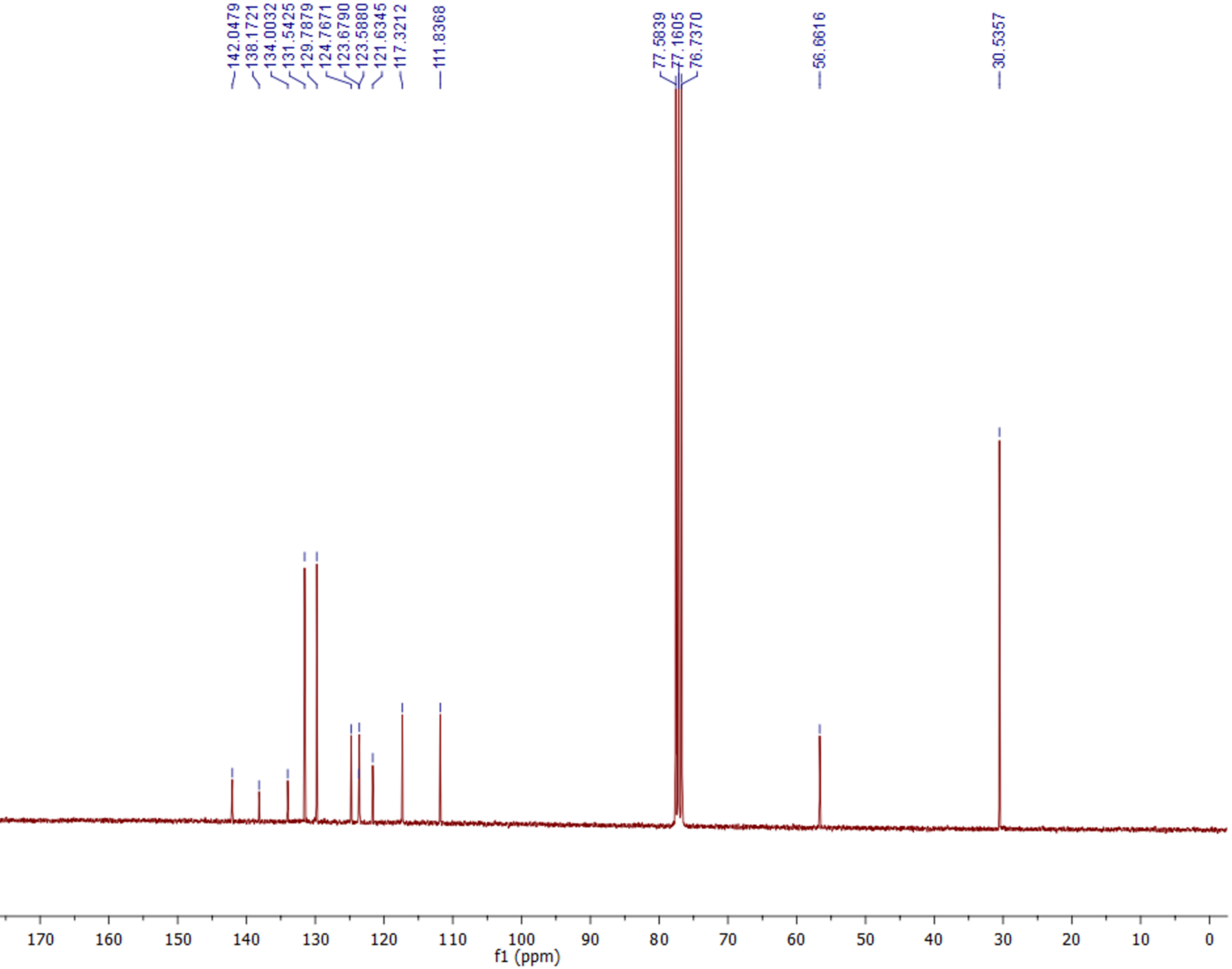

Figure S9. ^{1}H NMR spectra of **1e** (300 MHz, CDCl3, δ).

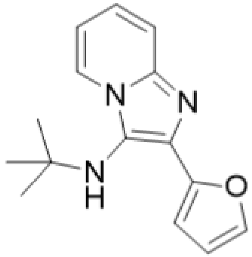

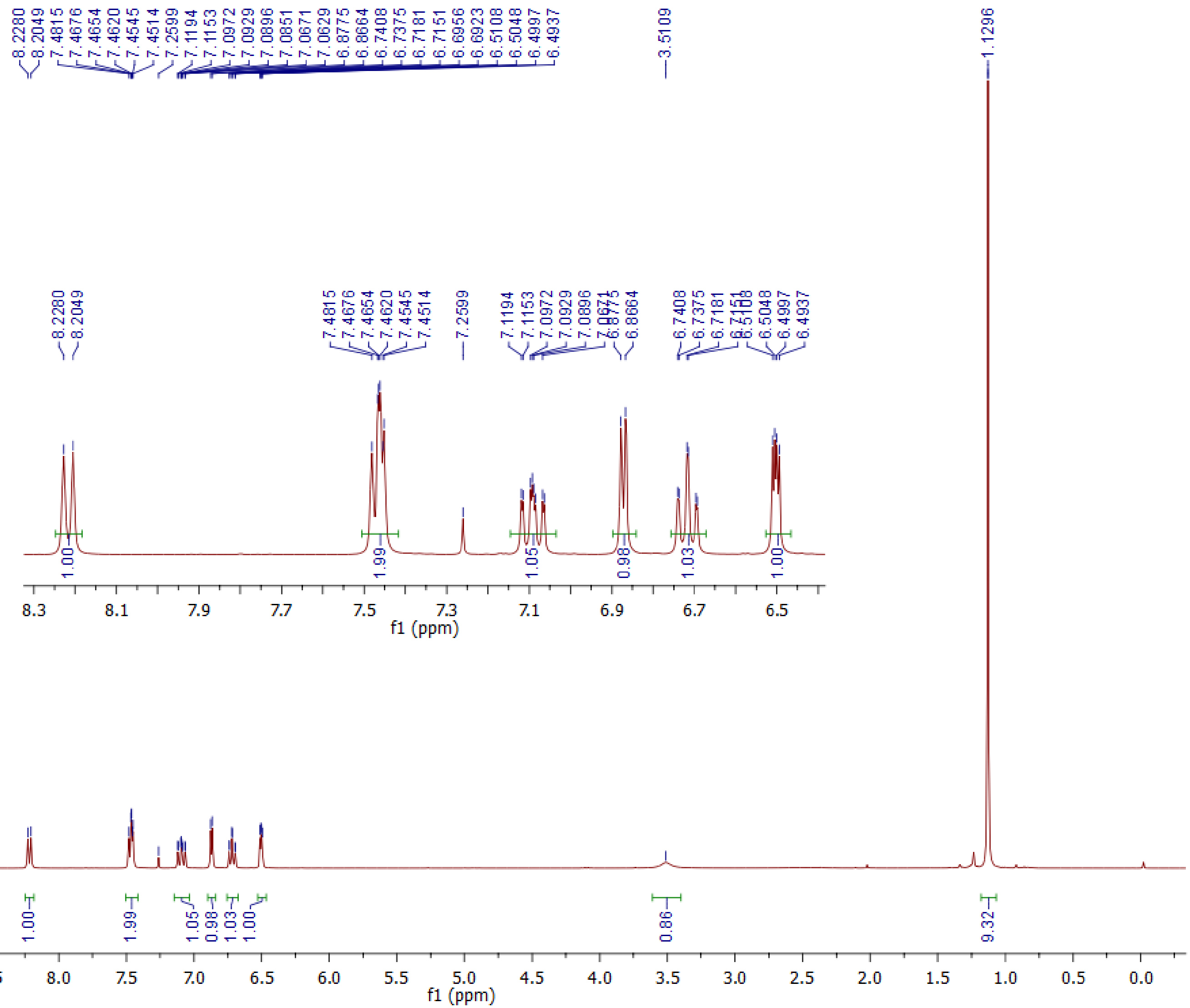

Figure S10. ^{13}C NMR spectra of **1e** (75 MHz, $CDCl_3$, δ).

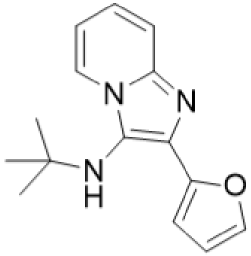

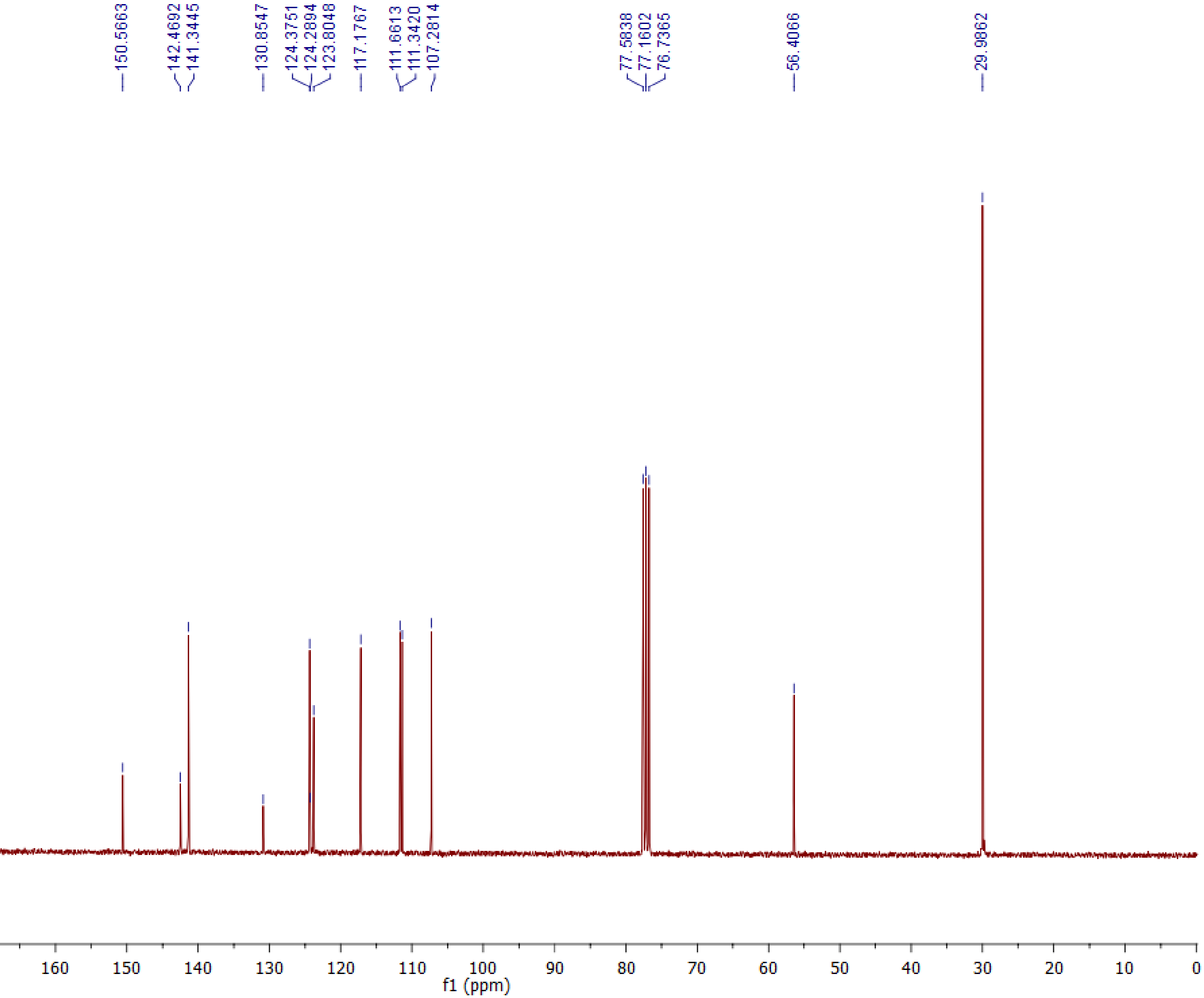

Figure S11. ^{1}H NMR spectra of **1f** (300 MHz, $CDCl_3$, δ).

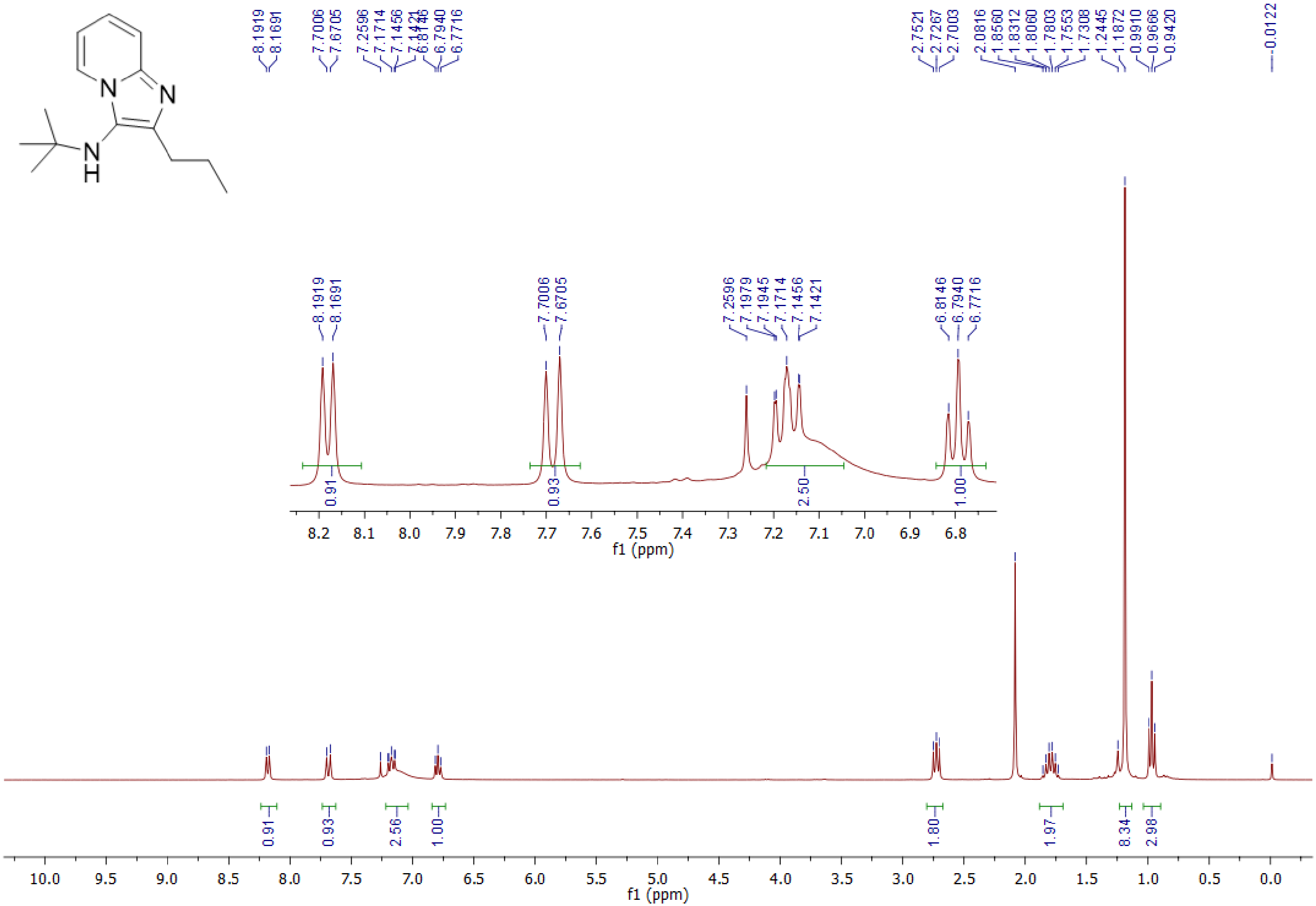

Figure S12. ^{13}C NMR spectra of **1f** (75 MHz, $CDCl_3$, δ).

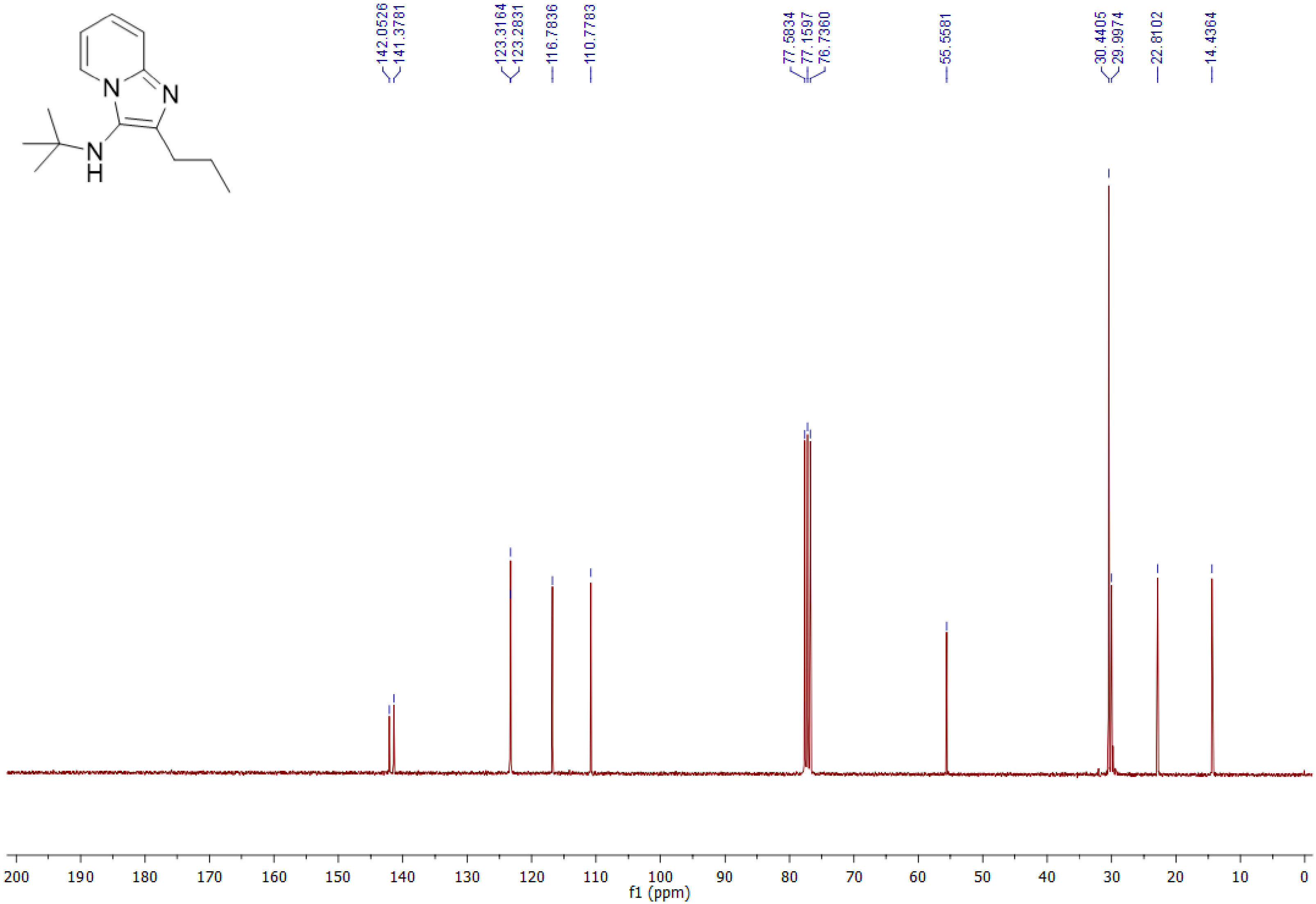

Figure S13. ^{1}H NMR spectra of **1g** (300 MHz, $CDCl_3$, δ).

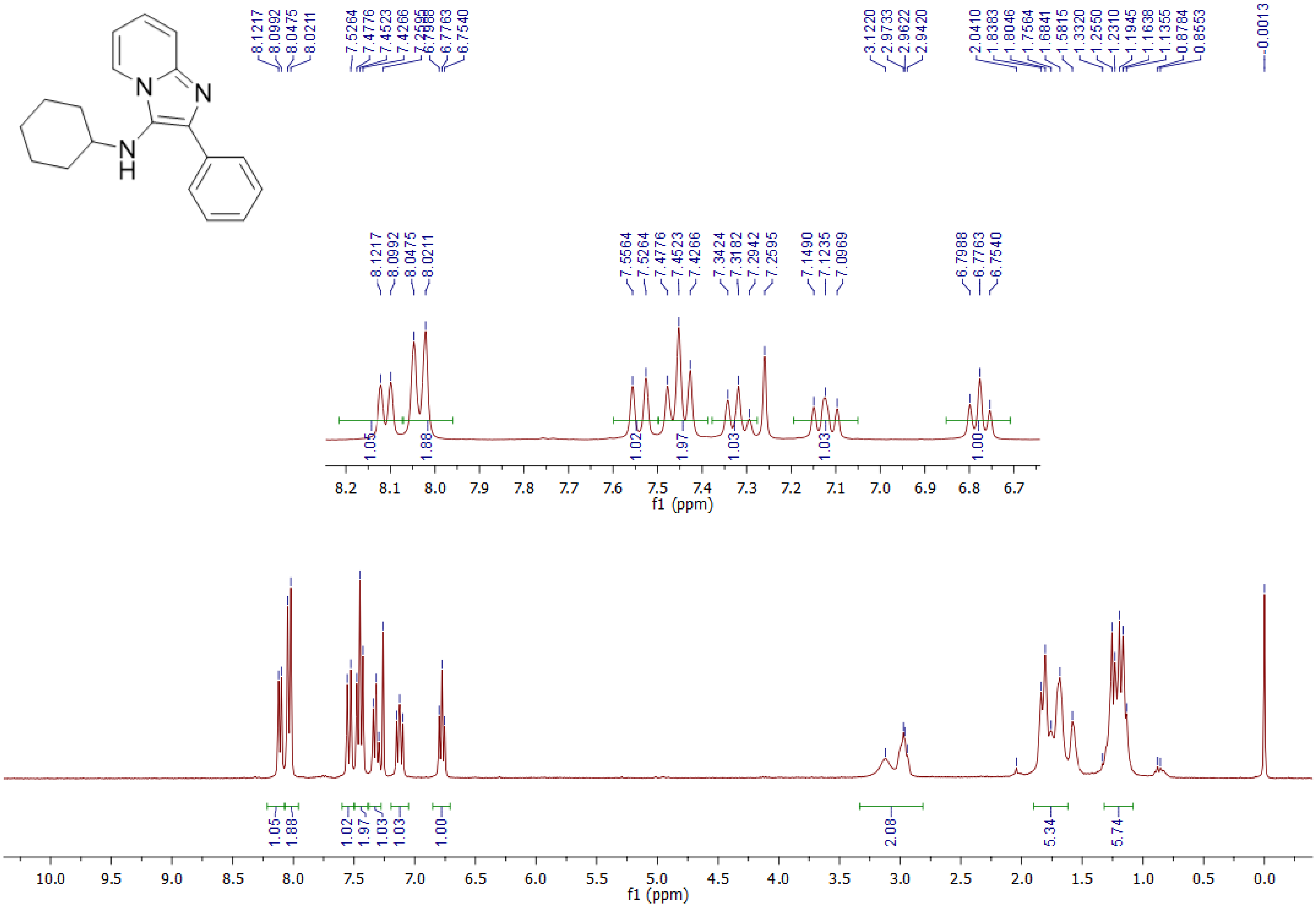

Figure S14. ^{13}C NMR spectra of **1g** (75 MHz, $CDCl_3$, δ).

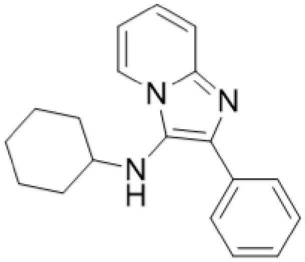

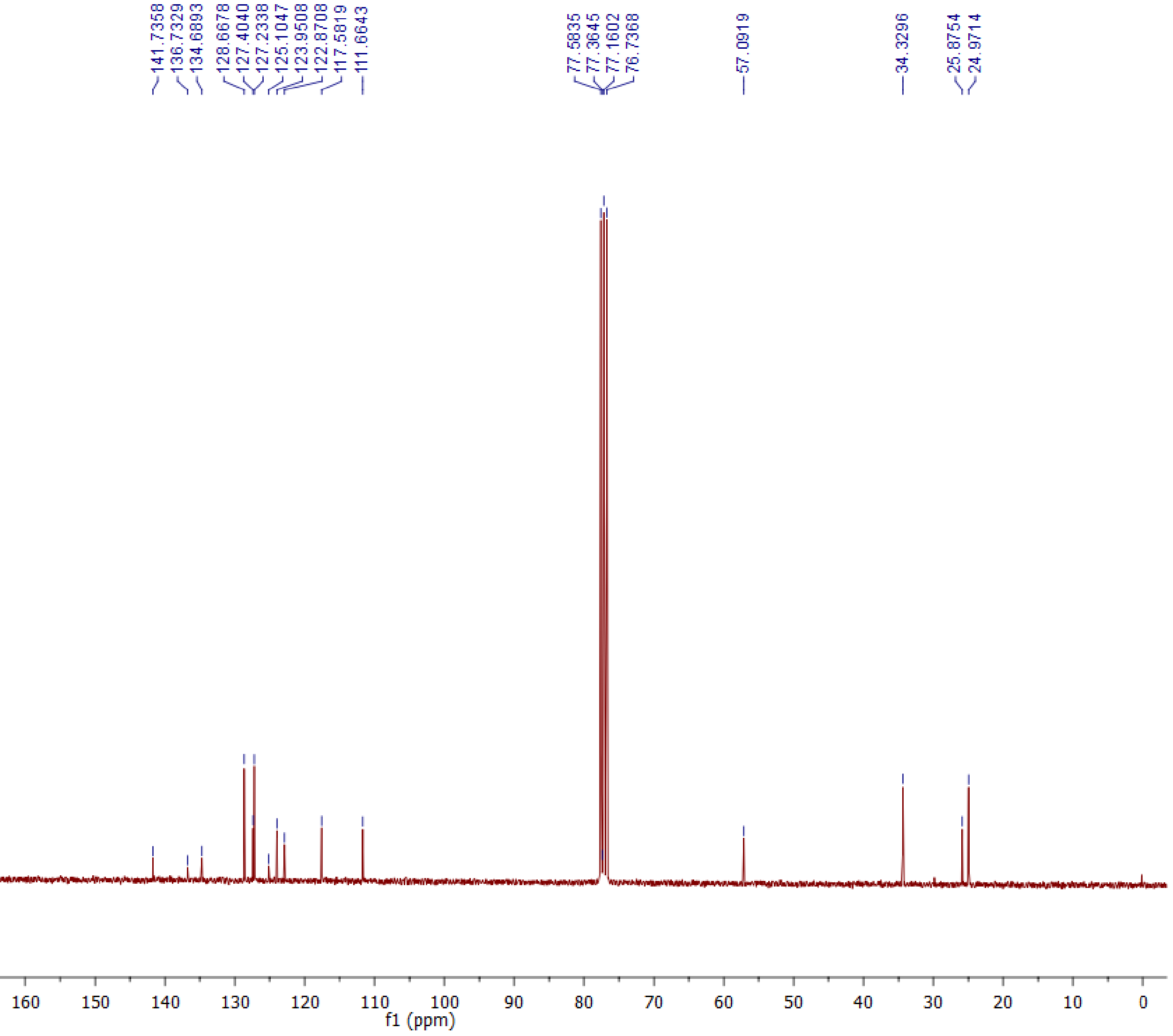

Figure S15. ^{1}H NMR spectra of **1h** (300 MHz, CDCl₃, δ).

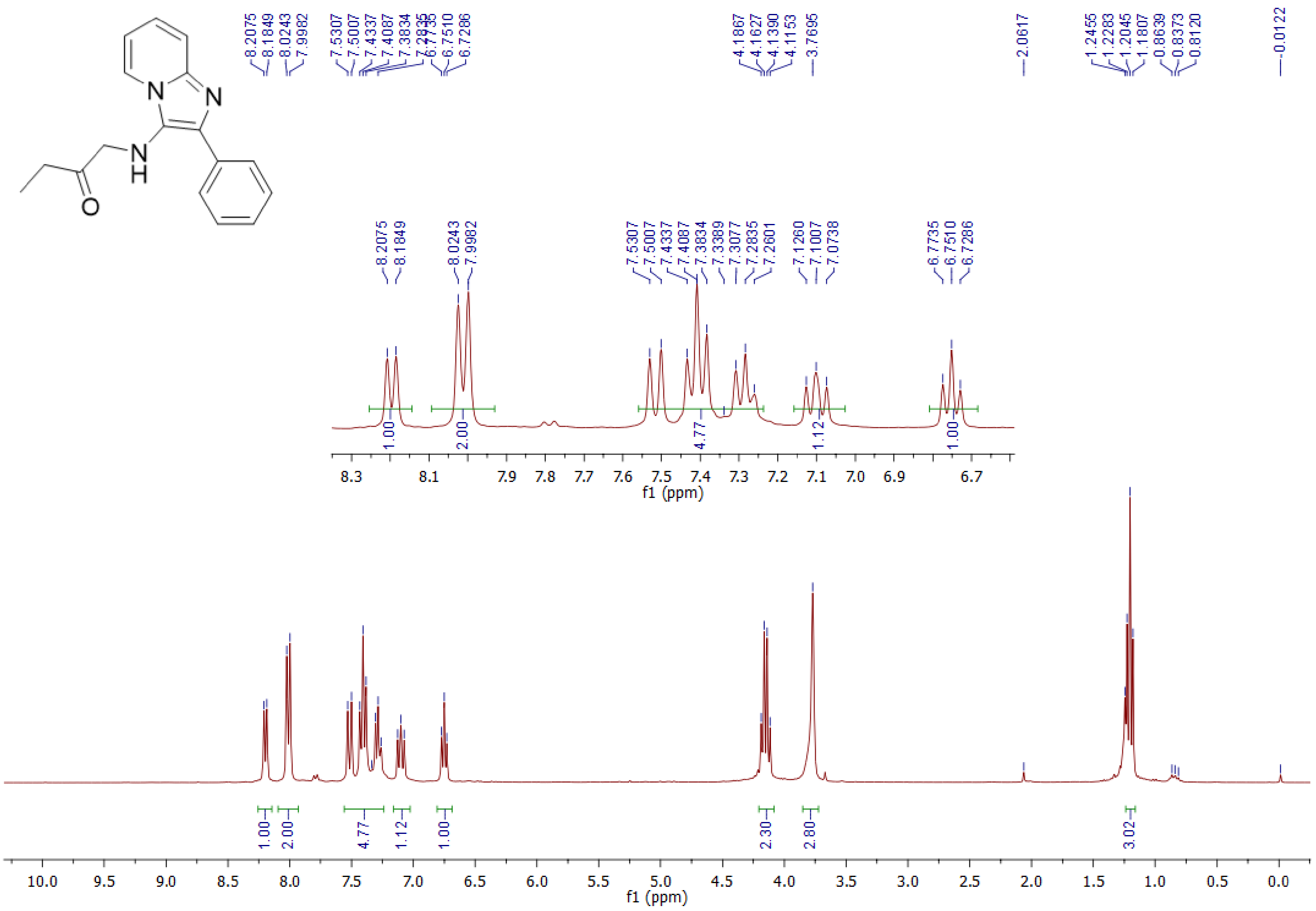

Figure S16. ^{13}C NMR spectra of **1h** (75 MHz, $CDCl_3$, δ).

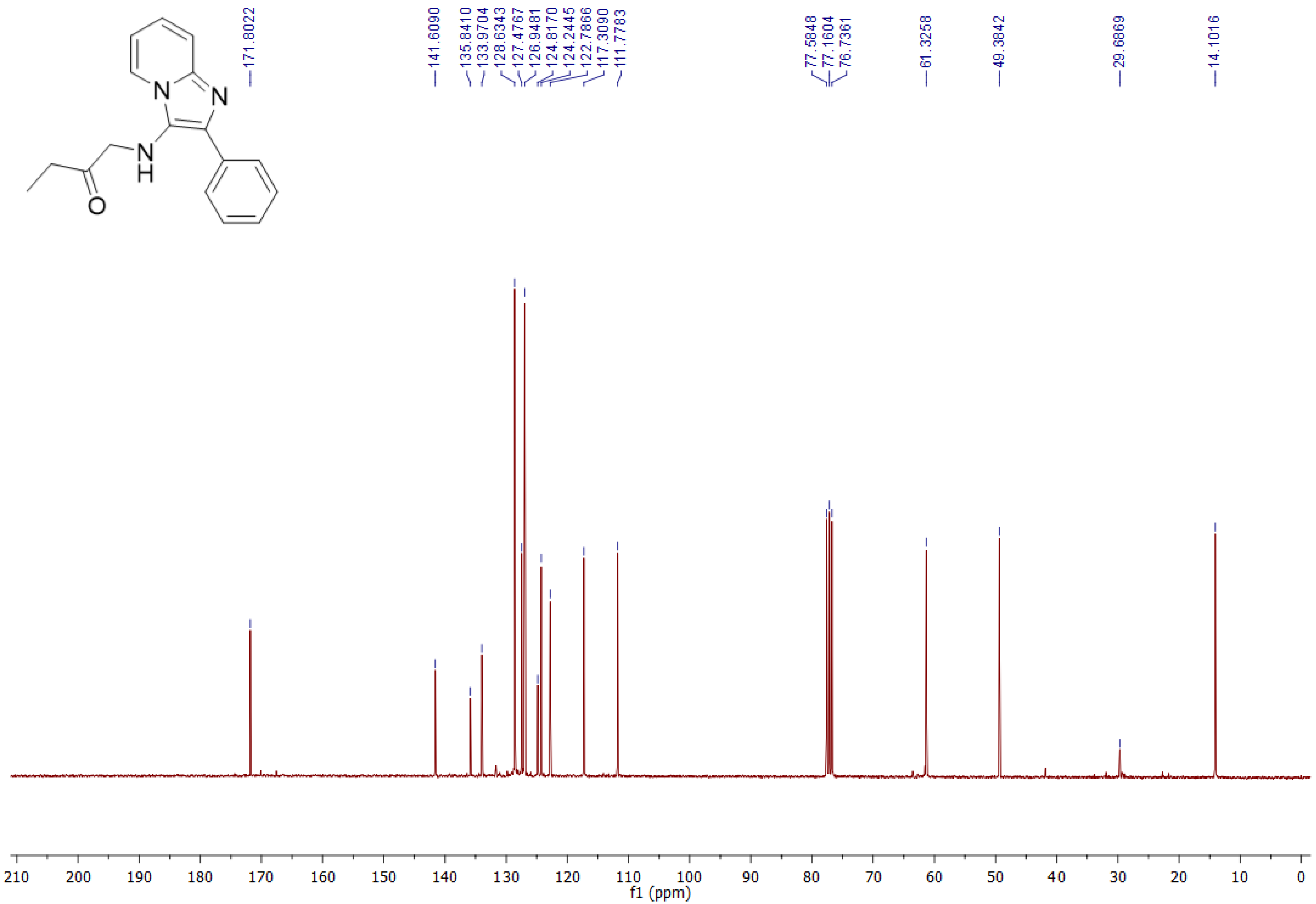

Figure S17. ^1H NMR spectra of **1i** (300 MHz, $CDCl_3$, δ).

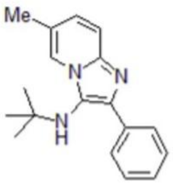

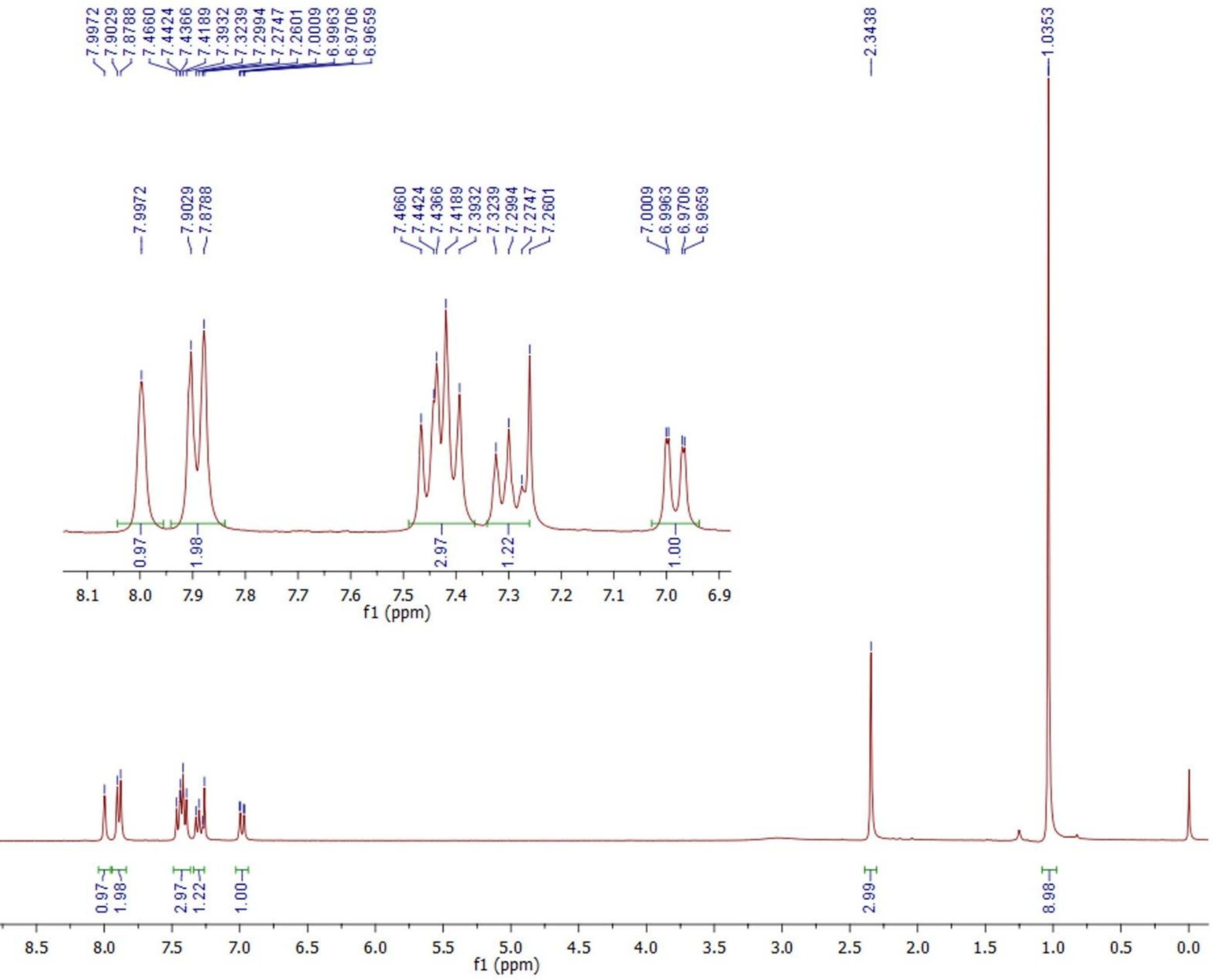

Figure S18. ^{13}C NMR spectra of **1i** (75 MHz, $CDCl_3$, δ).

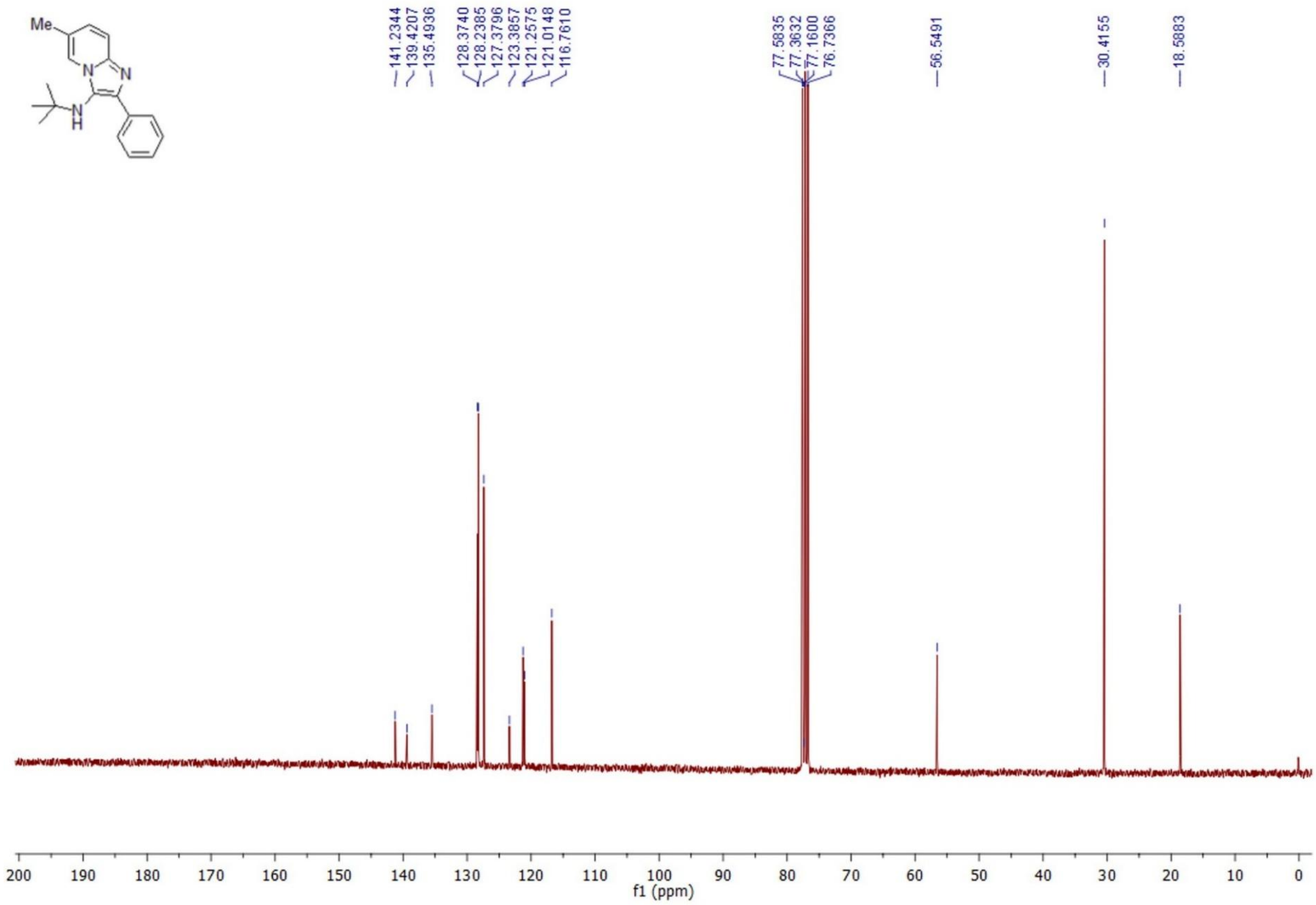

Figure S19. ^{1}H NMR spectra of **1j** (300 MHz, $CDCl_3$, δ).

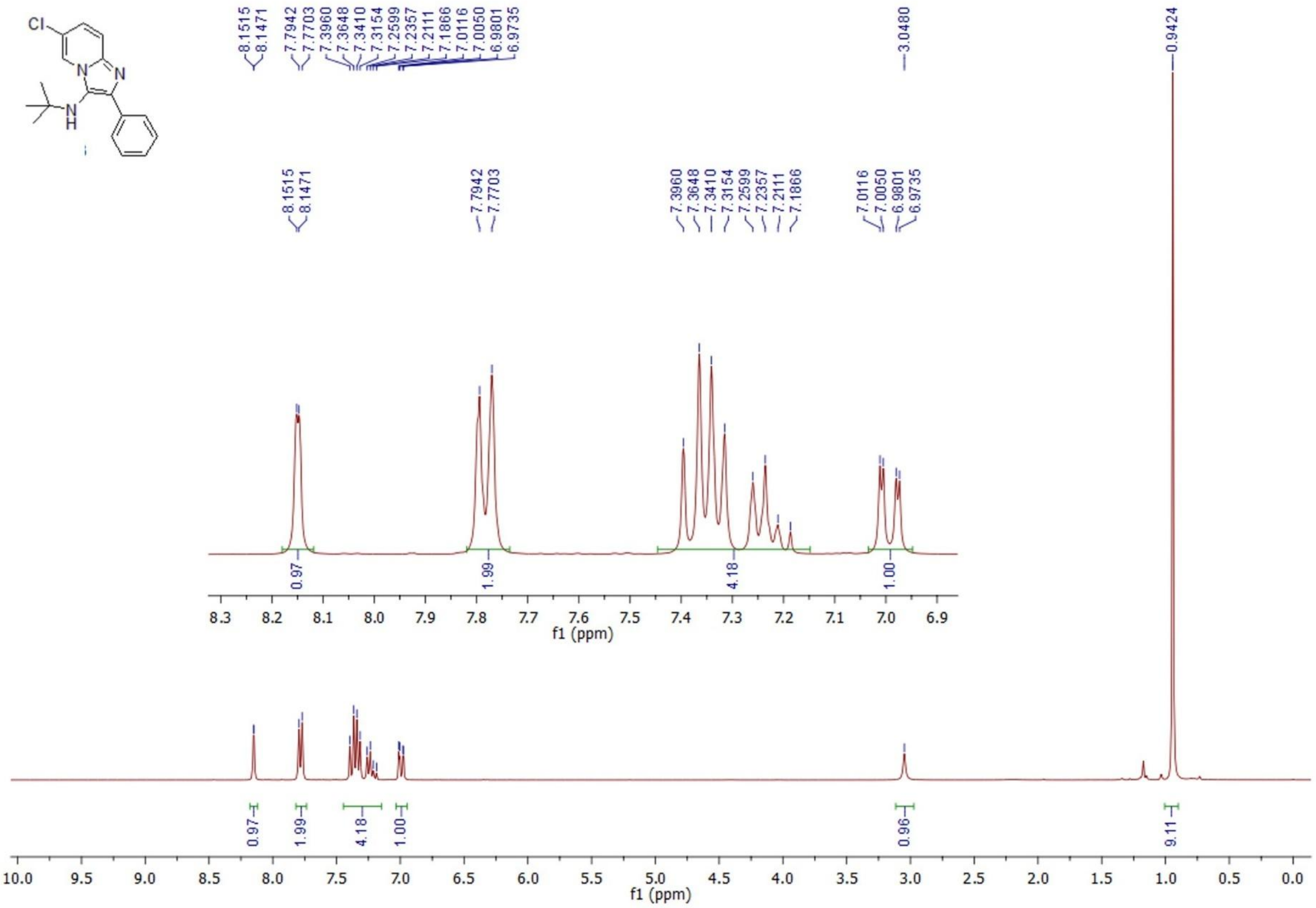

Figure S20. ^{13}C NMR spectra of **1j** (75 MHz, $CDCl_3$, δ).

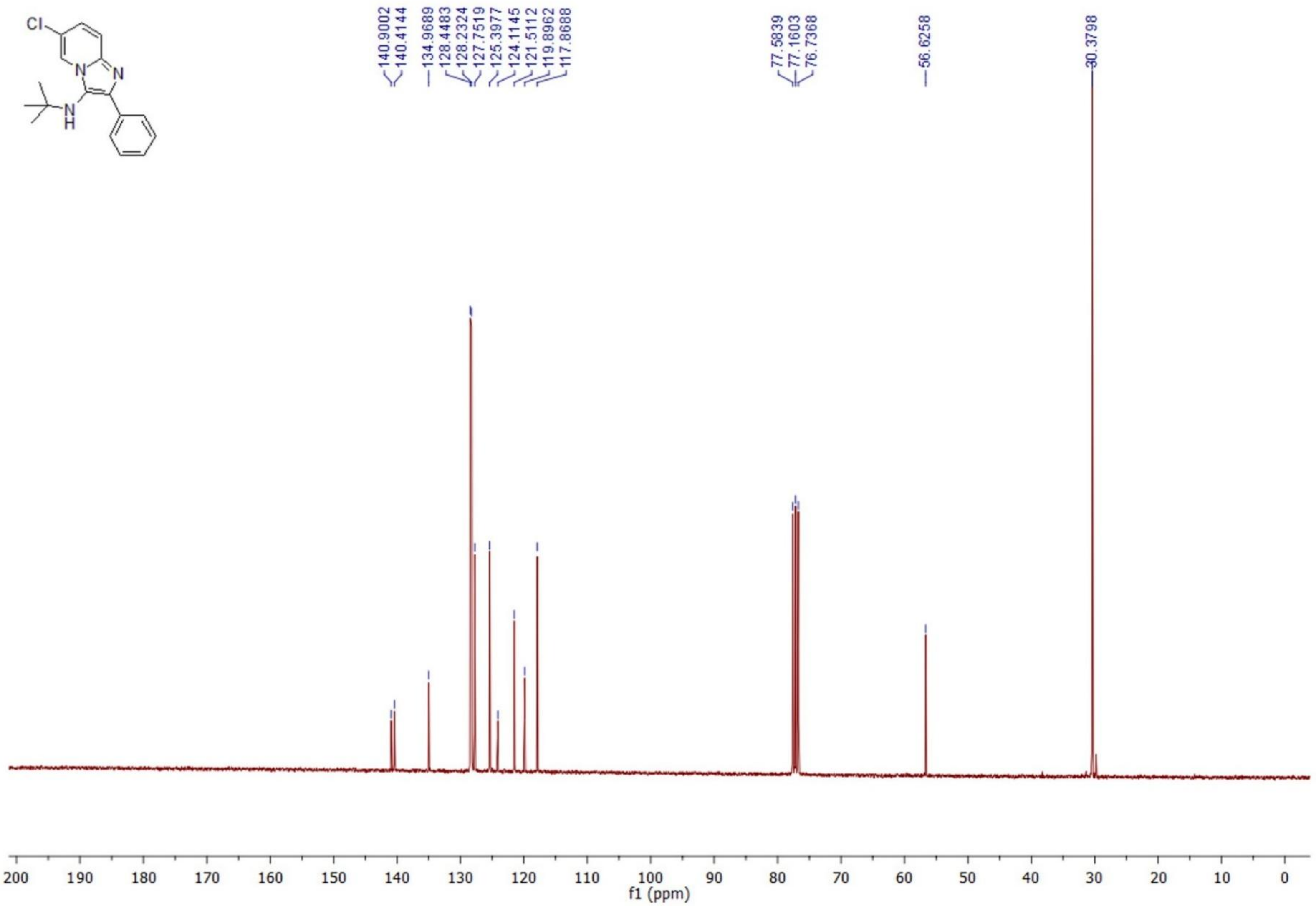

Figure S21. ^{1}H NMR spectra of **2a** (400 MHz, $CDCl_3$, δ).

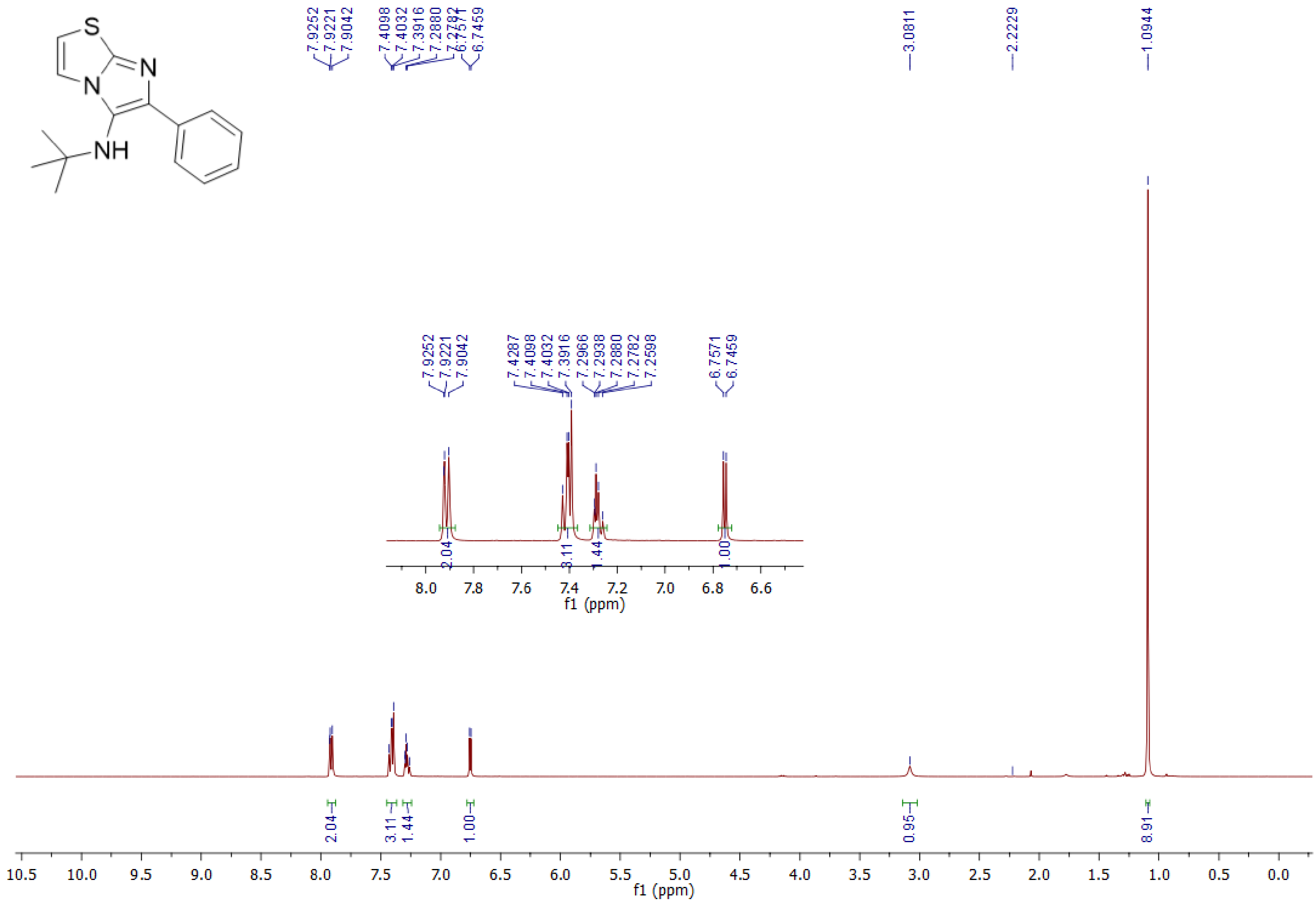

Figure S22. ^{13}C NMR spectra of **2a** (100 MHz, $CDCl_3$, δ).

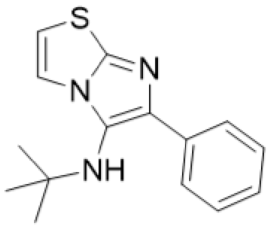

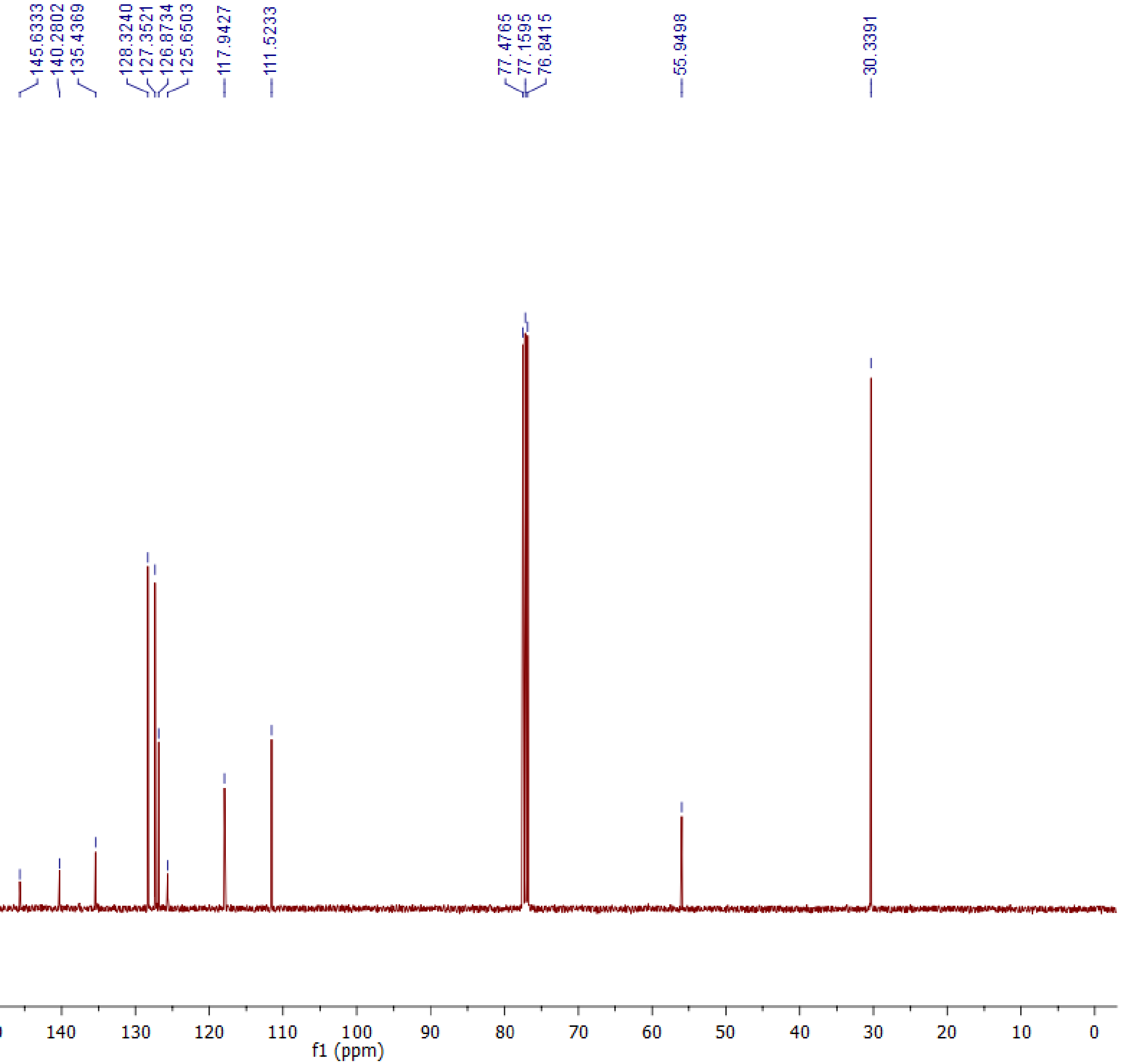

Figure S23. ^{1}H NMR spectra of **2b** (400 MHz, $CDCl_3$, δ).

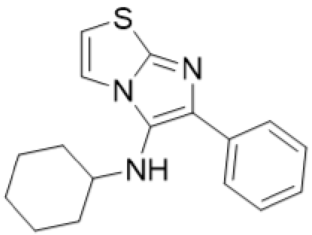

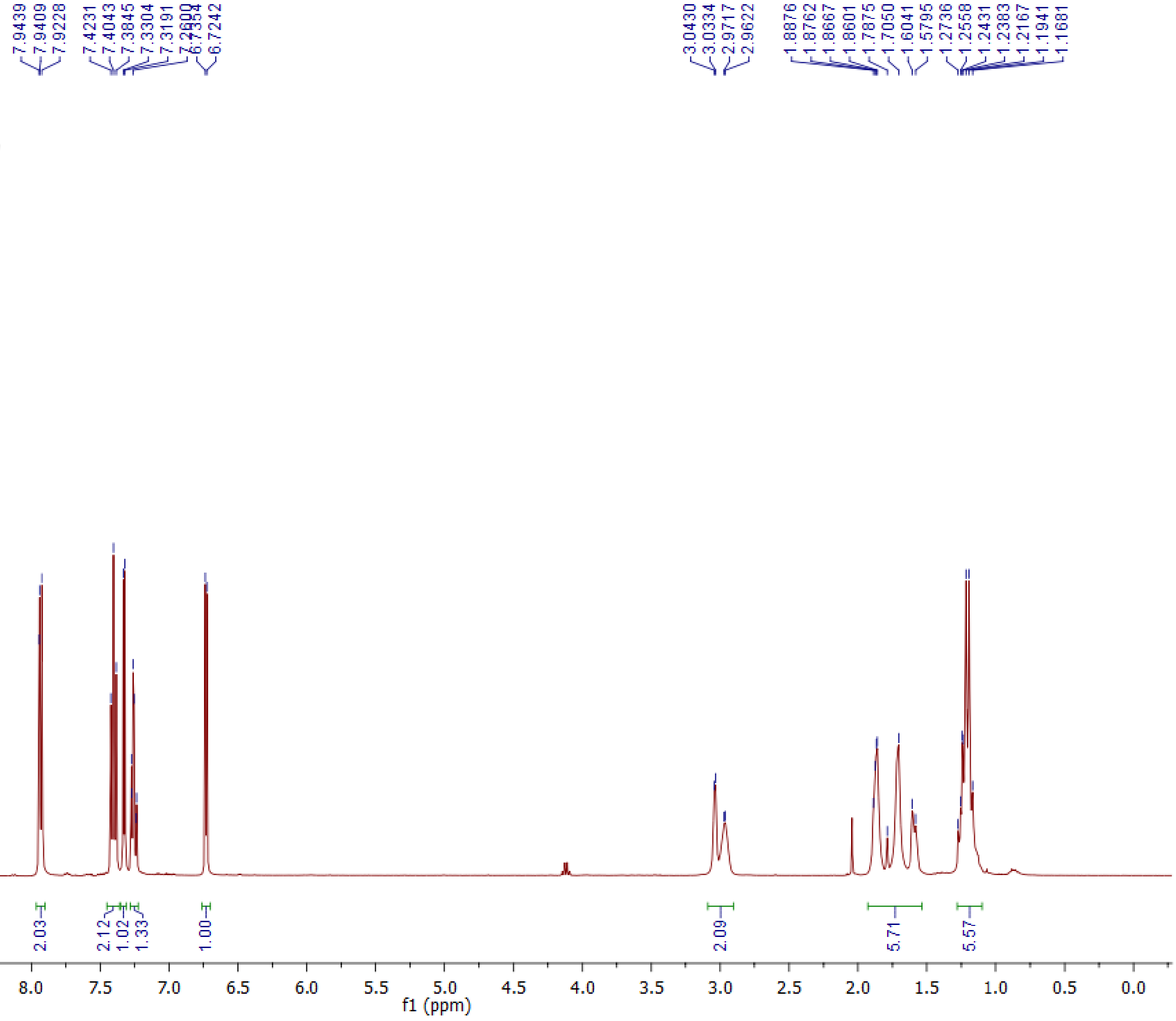

Figure S24. ^{13}C NMR spectra of **2b** (100 MHz, $CDCl_3$, δ).

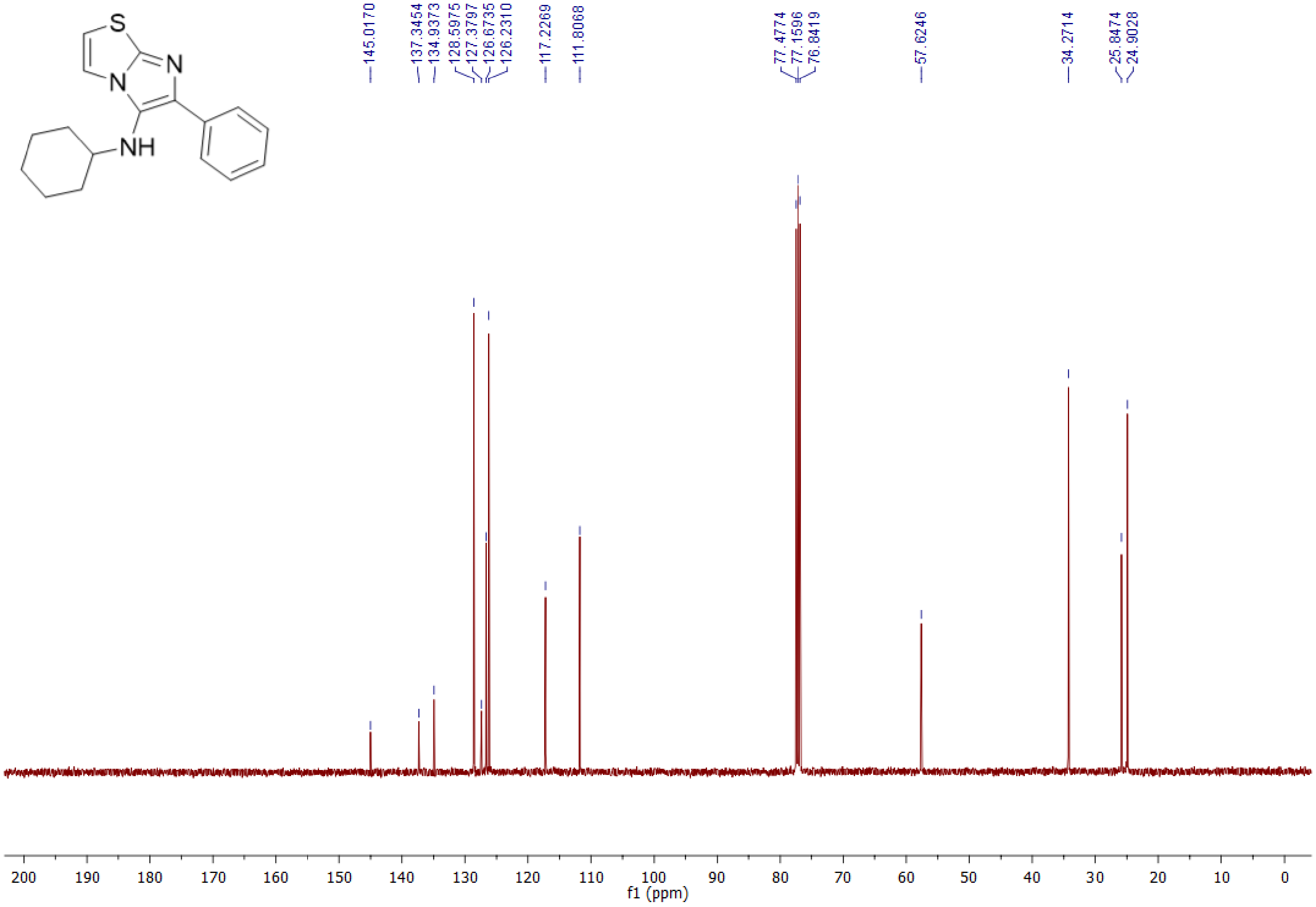

Figure S25. ^{1}H NMR spectra of **2c** (400 MHz, $CDCl_3$, δ).

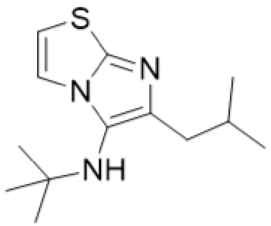

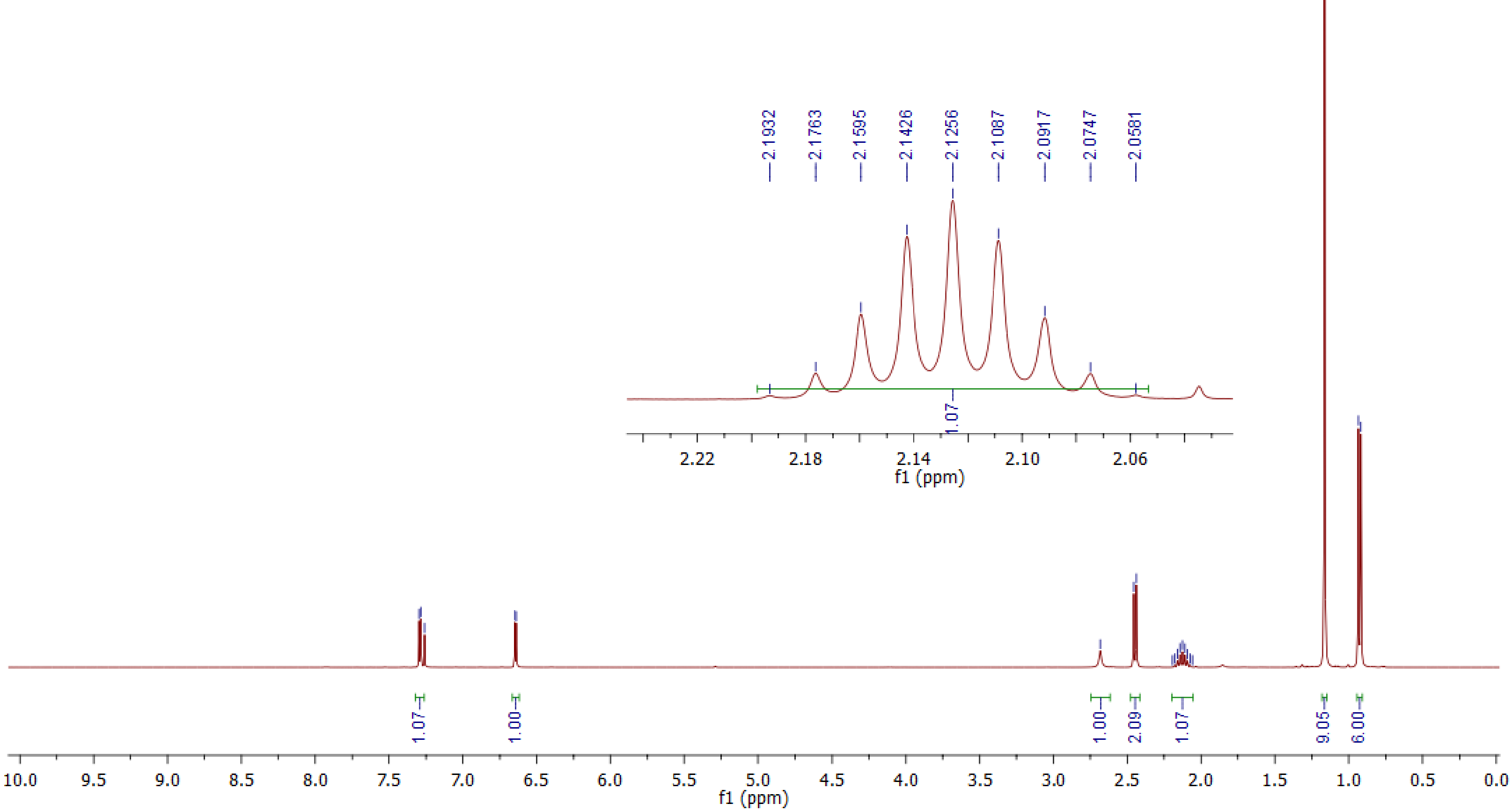

Figure S26. ^{13}C NMR spectra of **2c** (100 MHz, $CDCl_3$, δ).

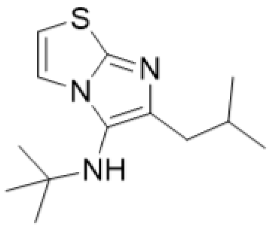

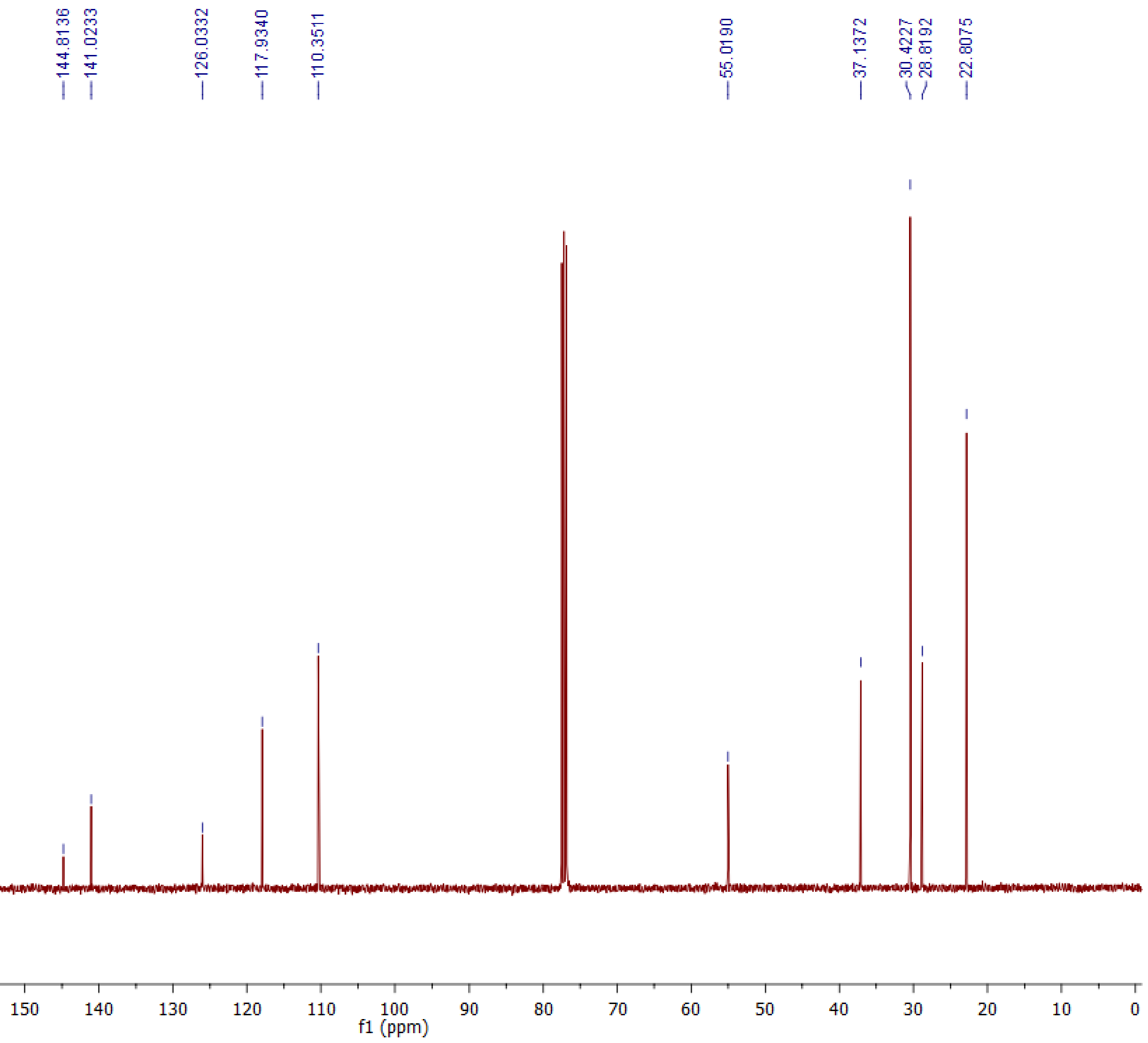

Figure S27. ^{1}H NMR spectra of **2d** (400 MHz, $CDCl_3$, δ).

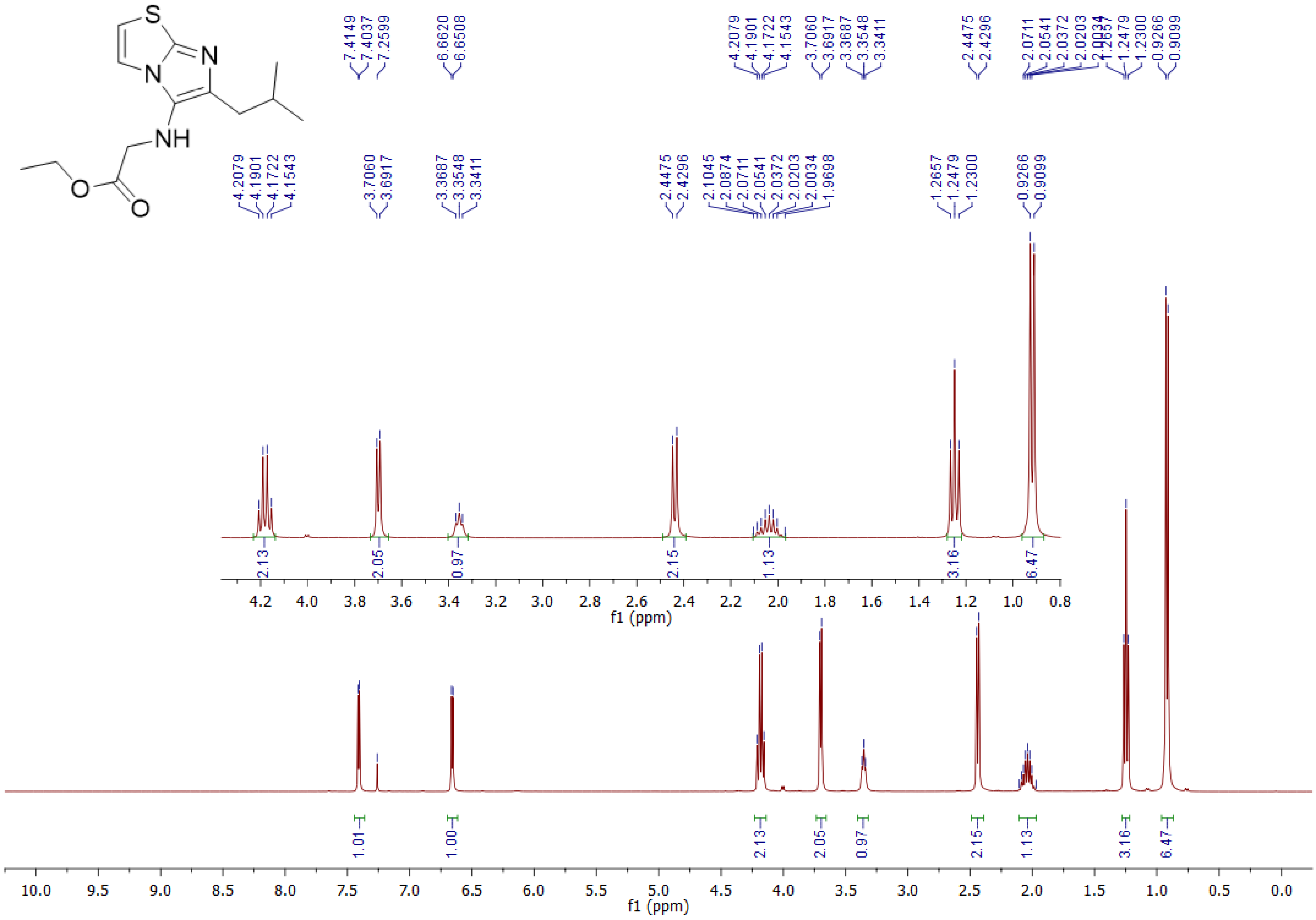

Figure S28. ^{13}C NMR spectra of **2d** (100 MHz, $CDCl_3$, δ).

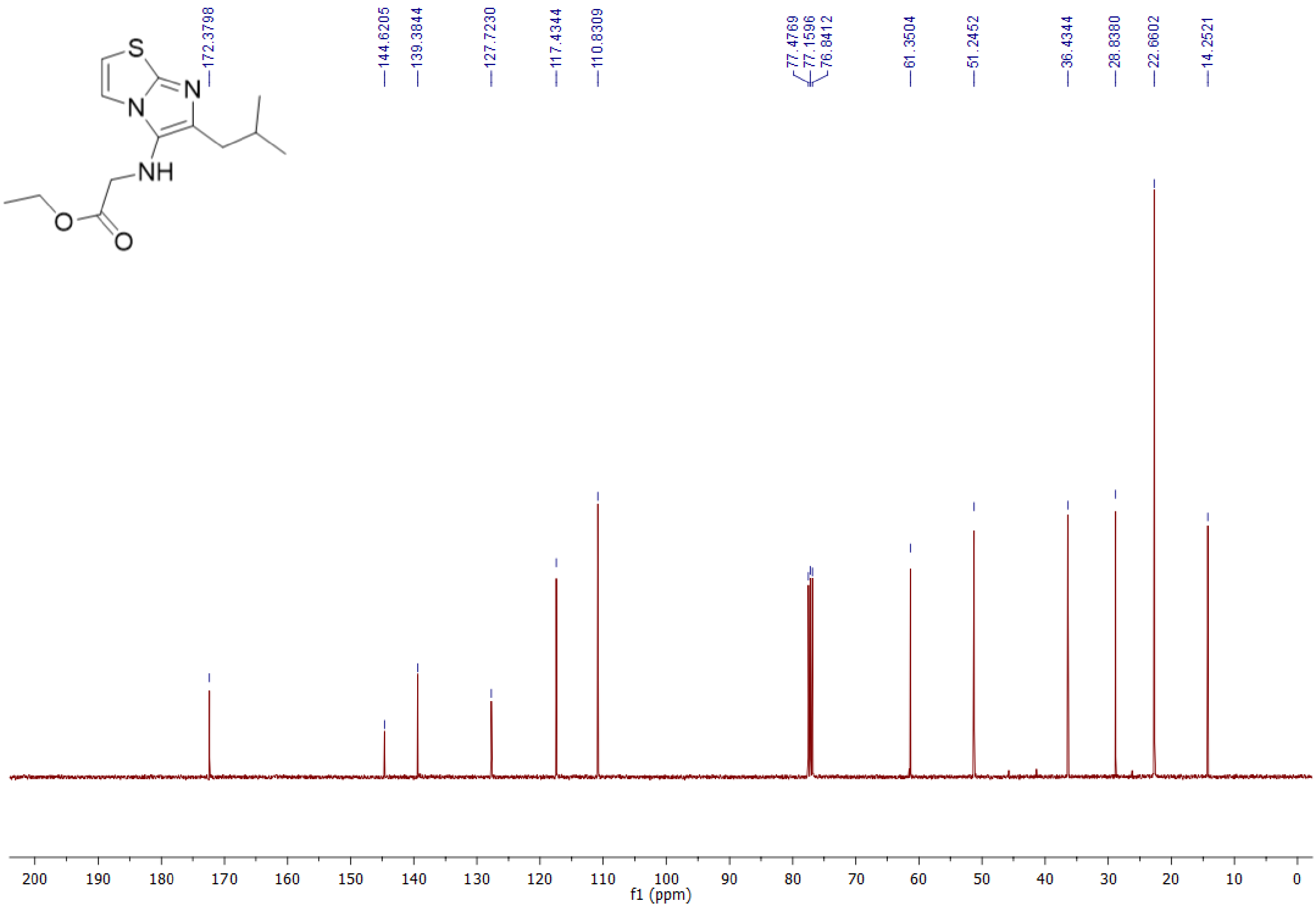

Figure S29. ^{1}H NMR spectra of **2e** (400 MHz, $CDCl_3$, δ).

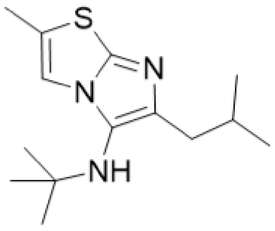

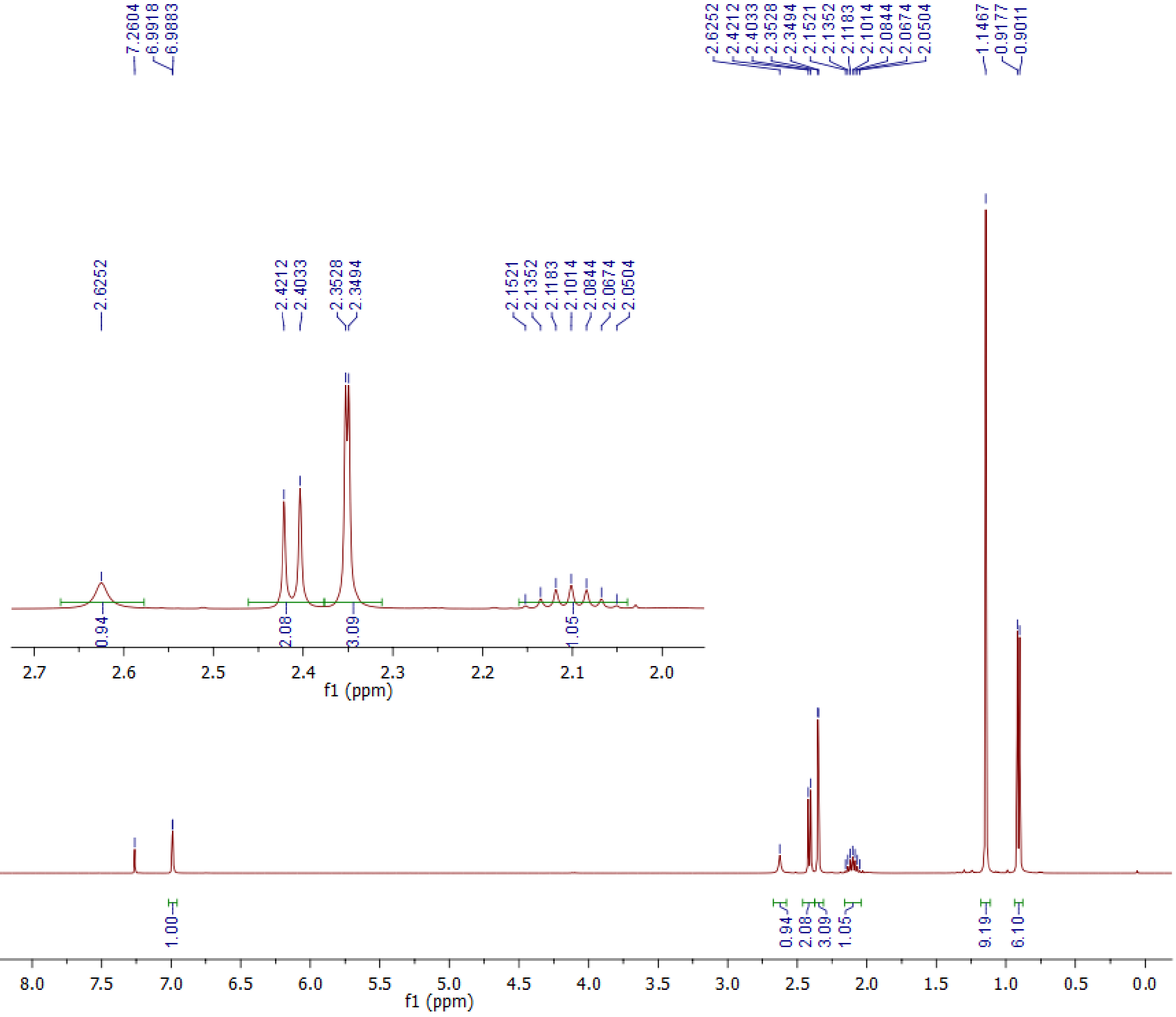

Figure S30. ^{13}C NMR spectra of **2e** (100 MHz, $CDCl_3$, δ).

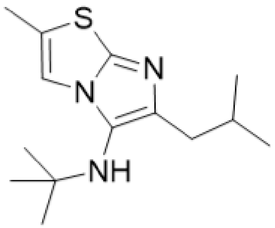

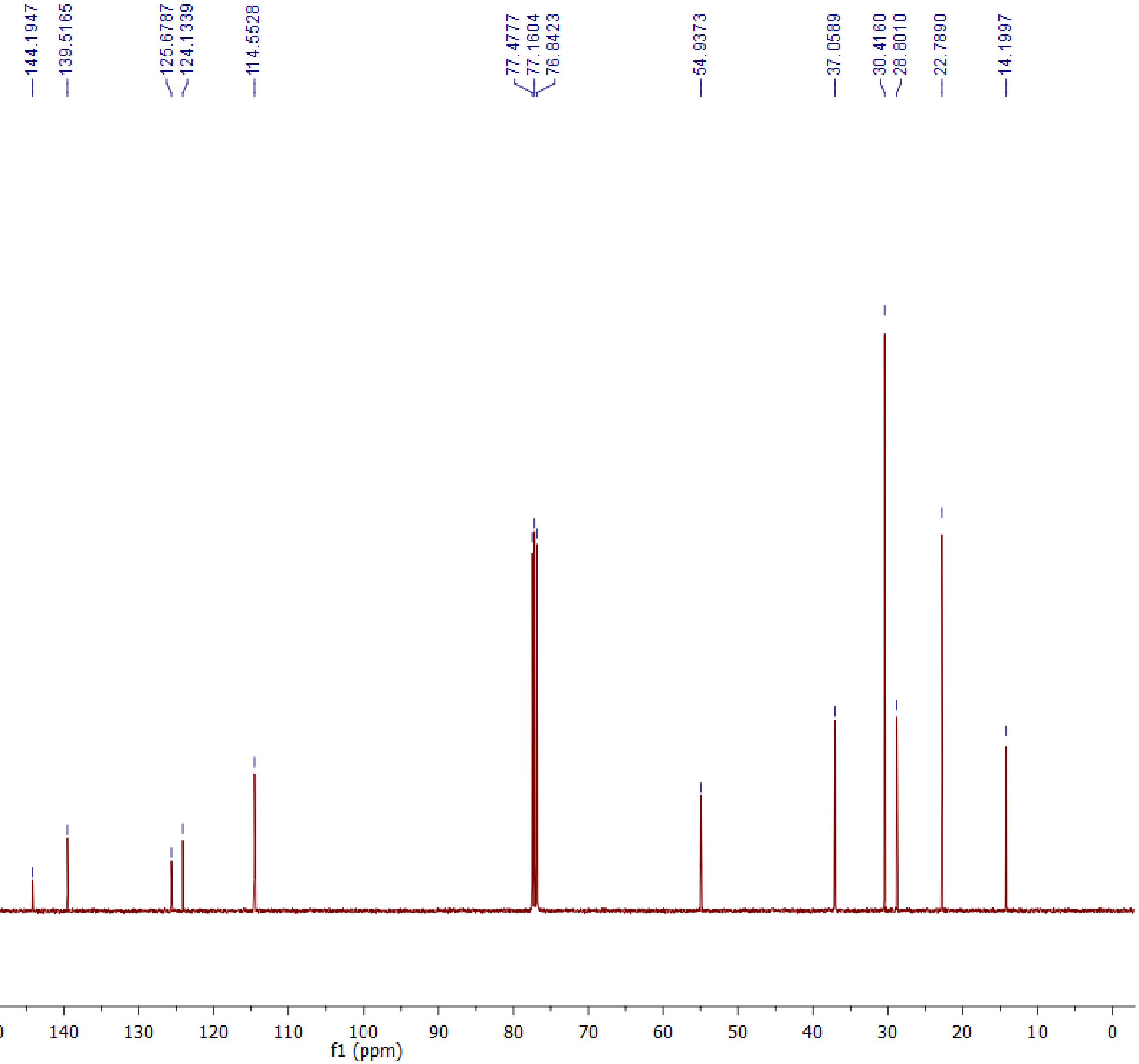

Figure S31. ^{1}H NMR spectra of **2f** (400 MHz, CDCl3, δ).

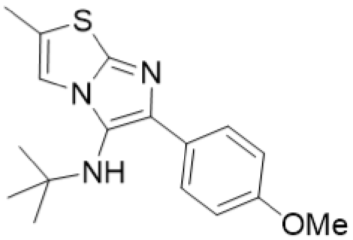

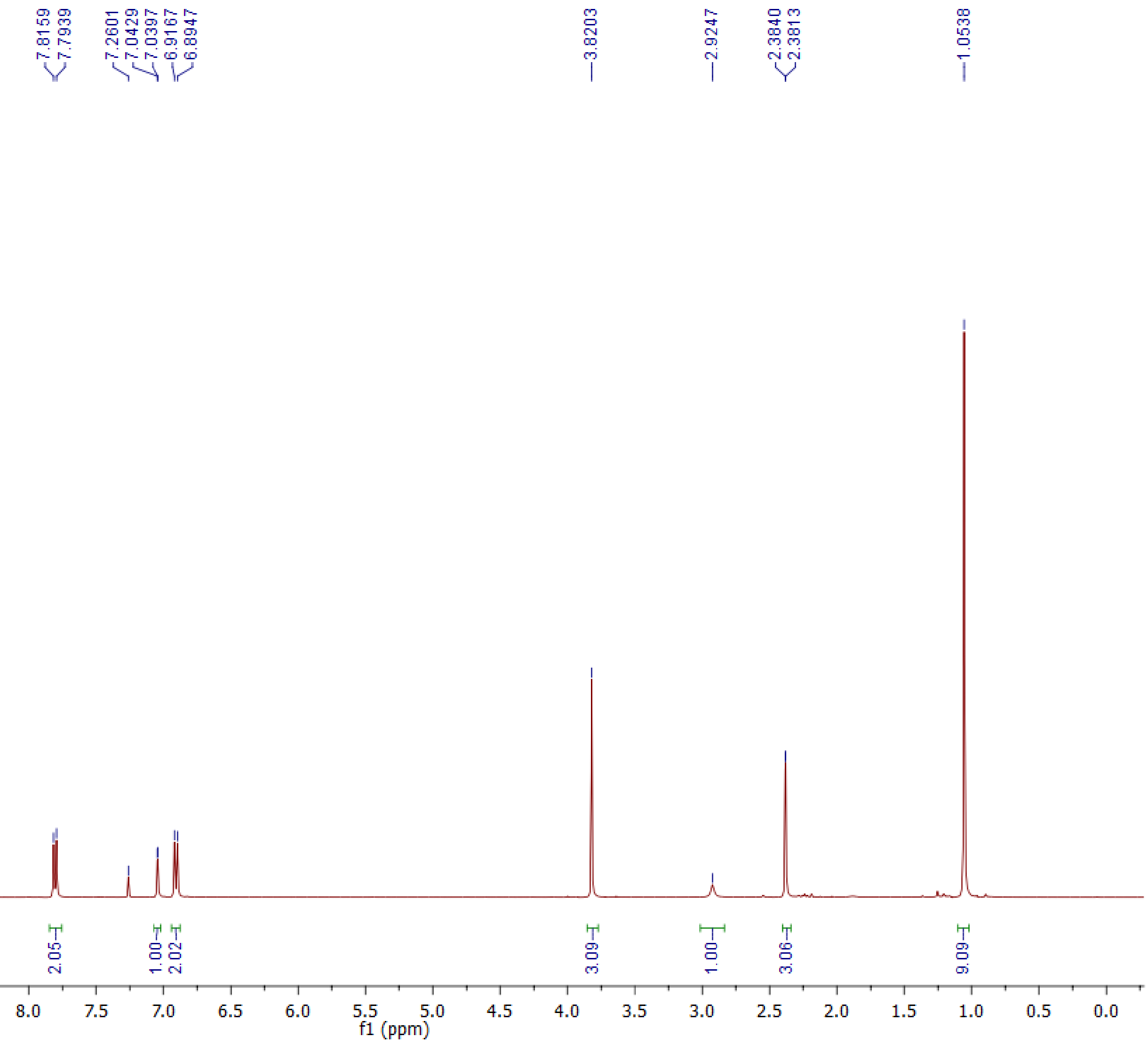

Figure S32. ^{13}C NMR spectra of **2f** (100 MHz, $CDCl_3$, δ).

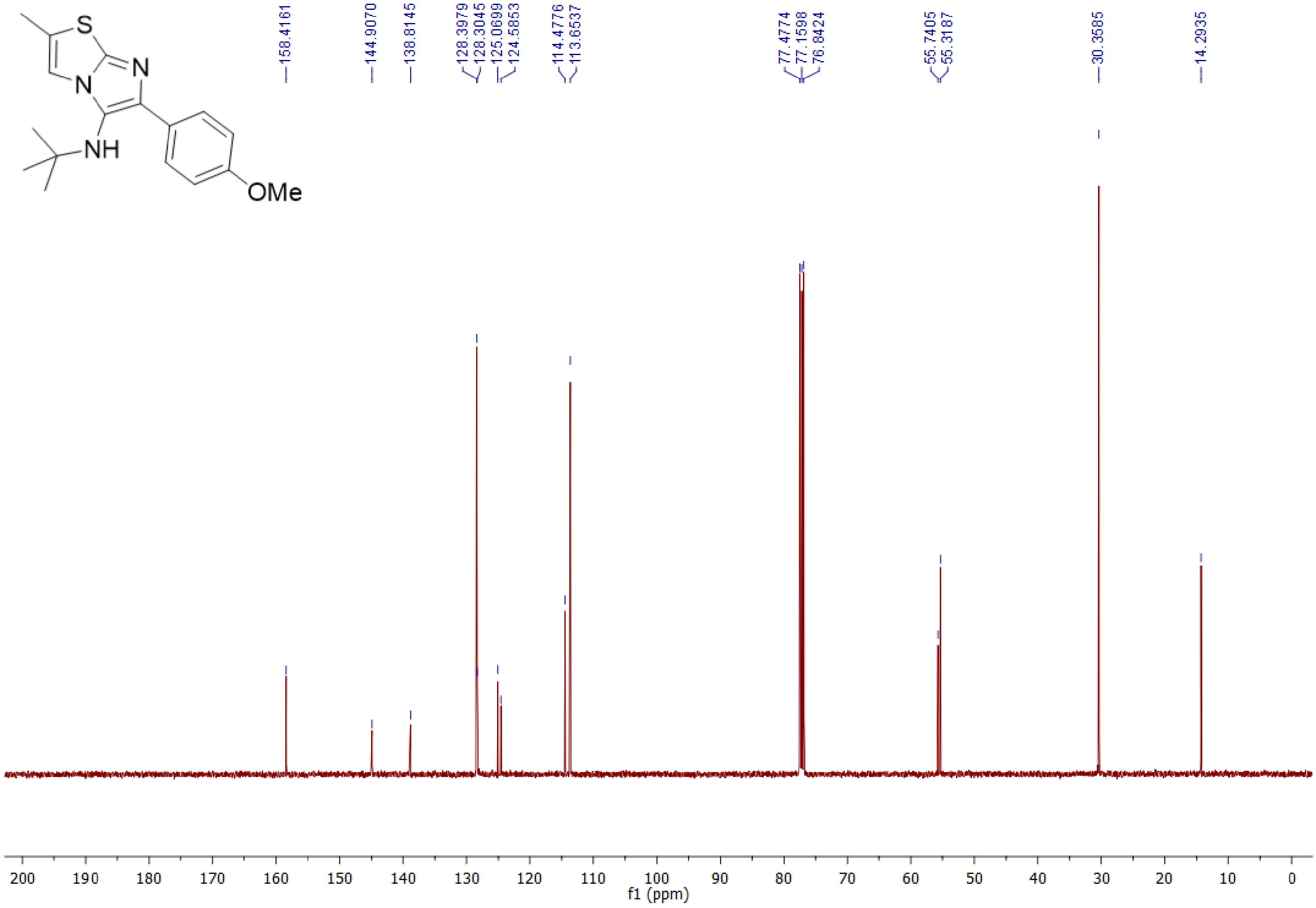

Figure S33. ^{1}H NMR spectra of **2g** (400 MHz, $CDCl_3$, δ).

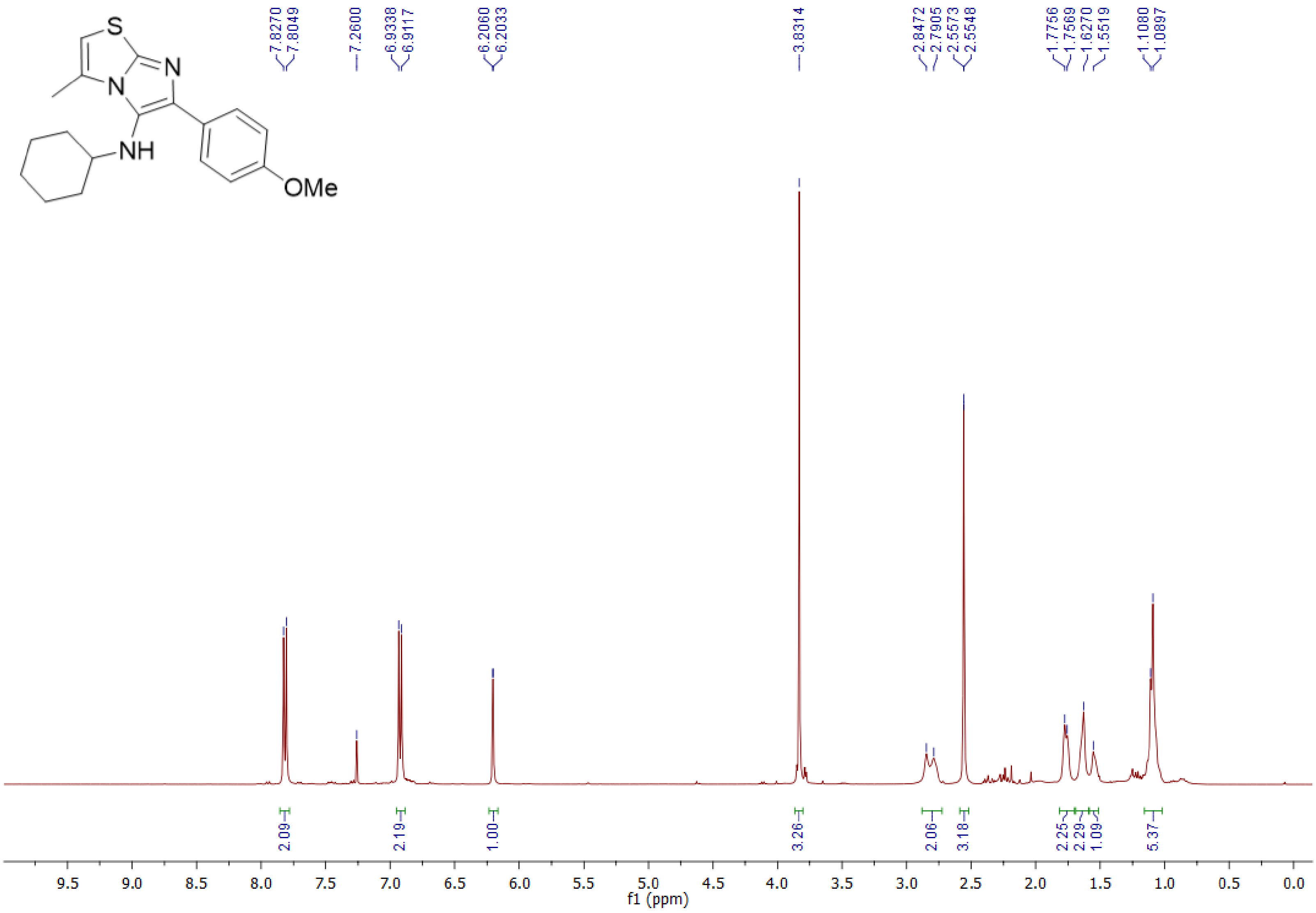

Figure S34. ^{13}C NMR spectra of **2g** (100 MHz, $CDCl_3$, δ).

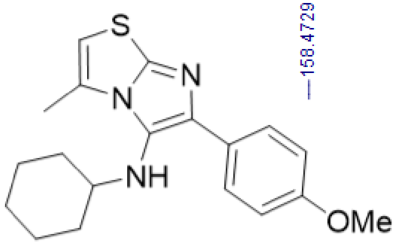

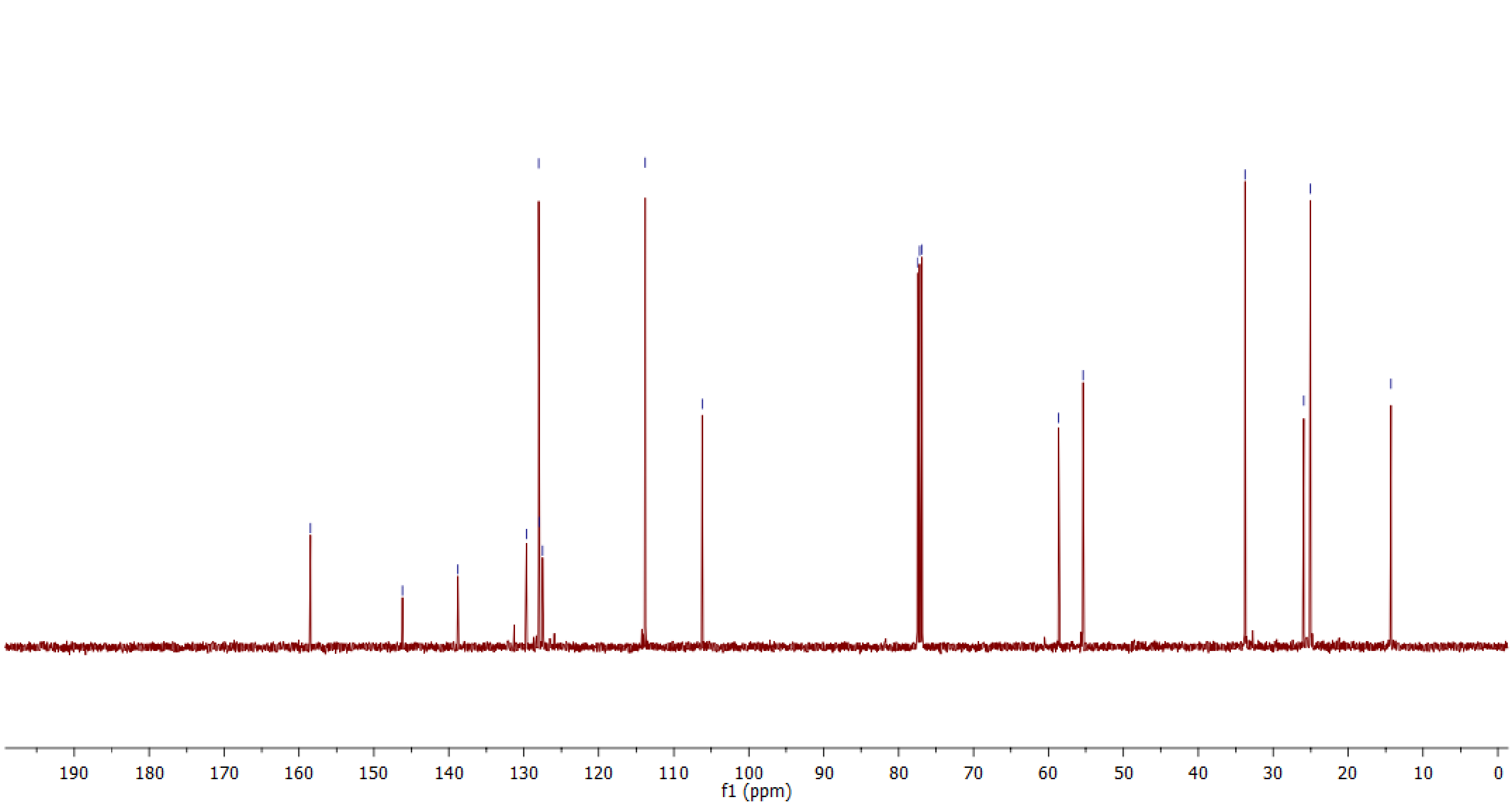

Figure S35. ^{1}H NMR spectra of **2h** (400 MHz, $CDCl_3$, δ).

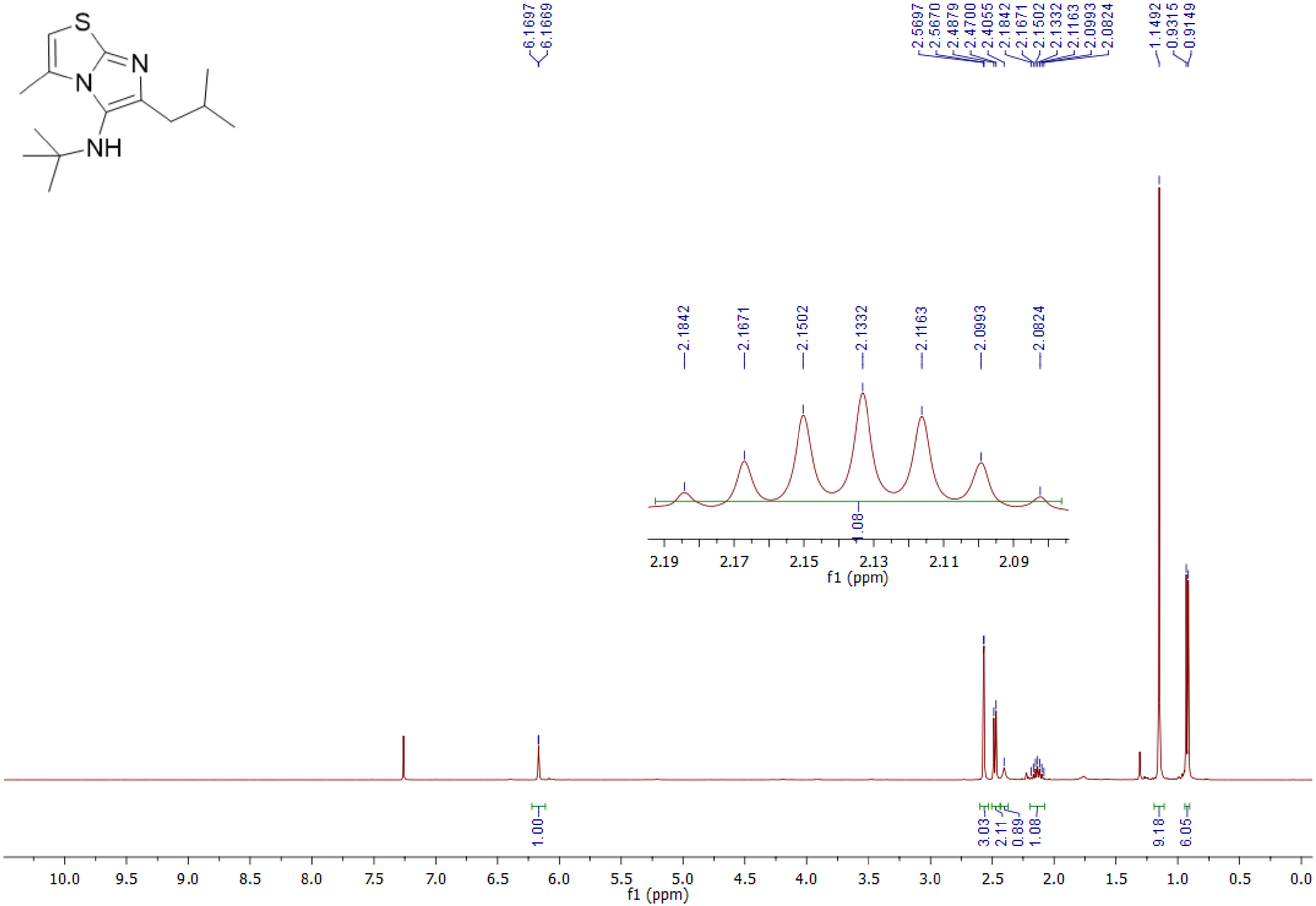

Figure S36. ^{13}C NMR spectra of **2h** (100 MHz, $CDCl_3$, δ).

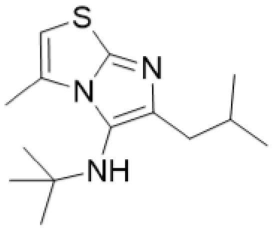

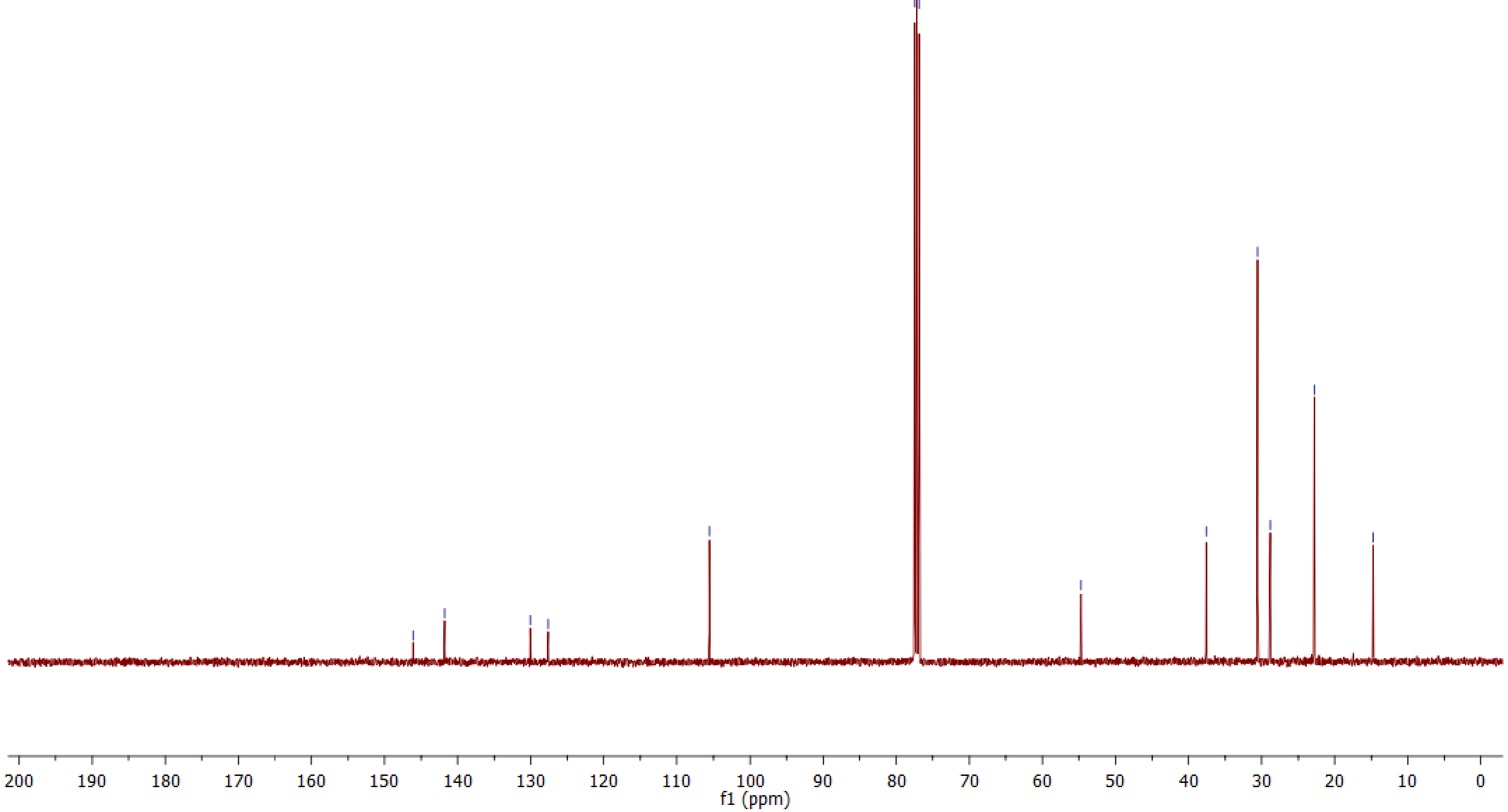

Figure S37. ^{1}H NMR spectra of **3a** (400 MHz, $CDCl_3$, δ).

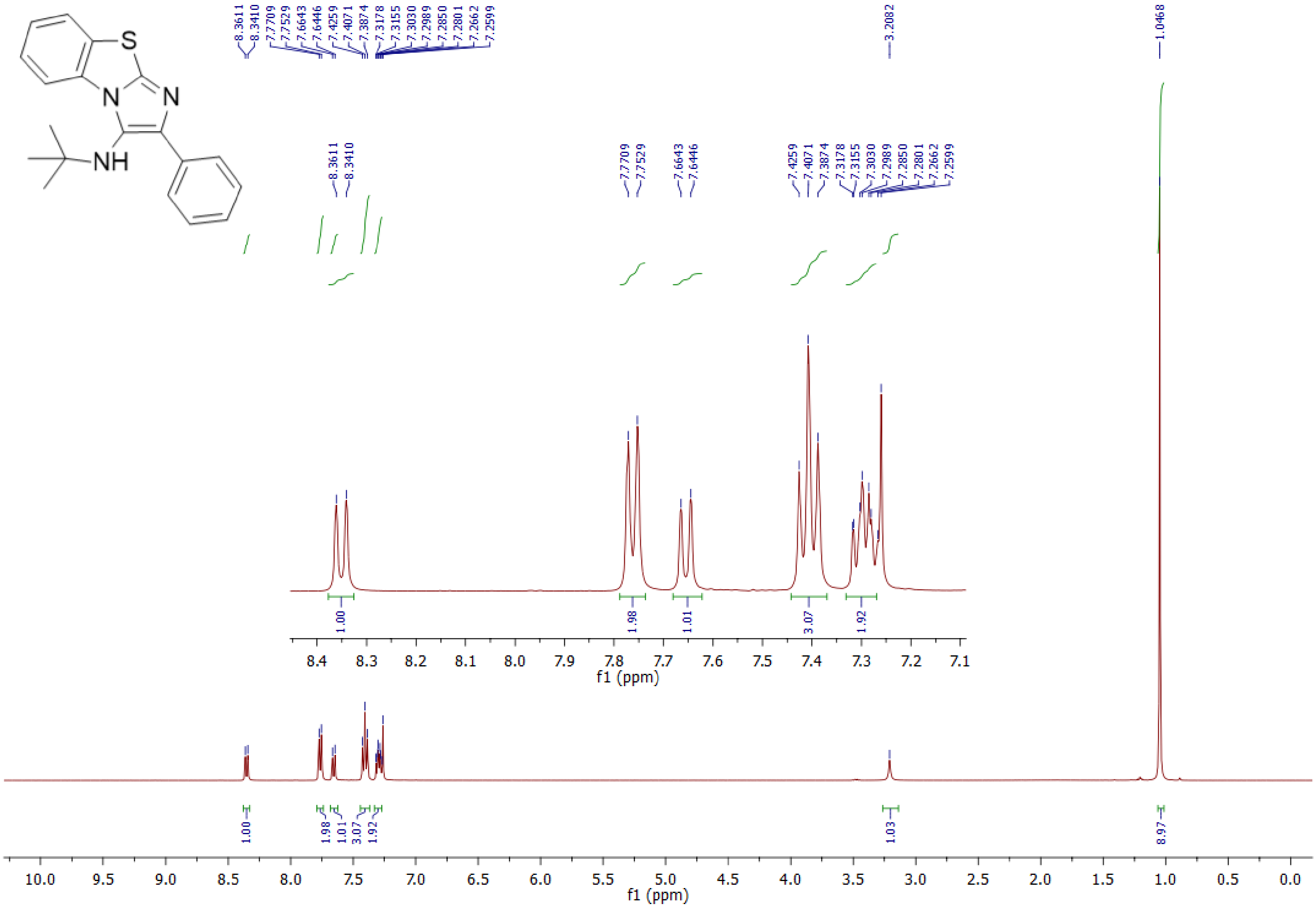

Figure S38. ^{13}C NMR spectra of **3a** (100 MHz, $CDCl_3$, δ).

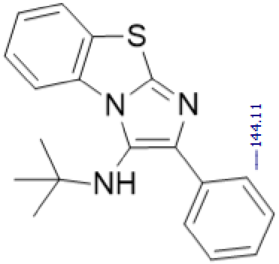

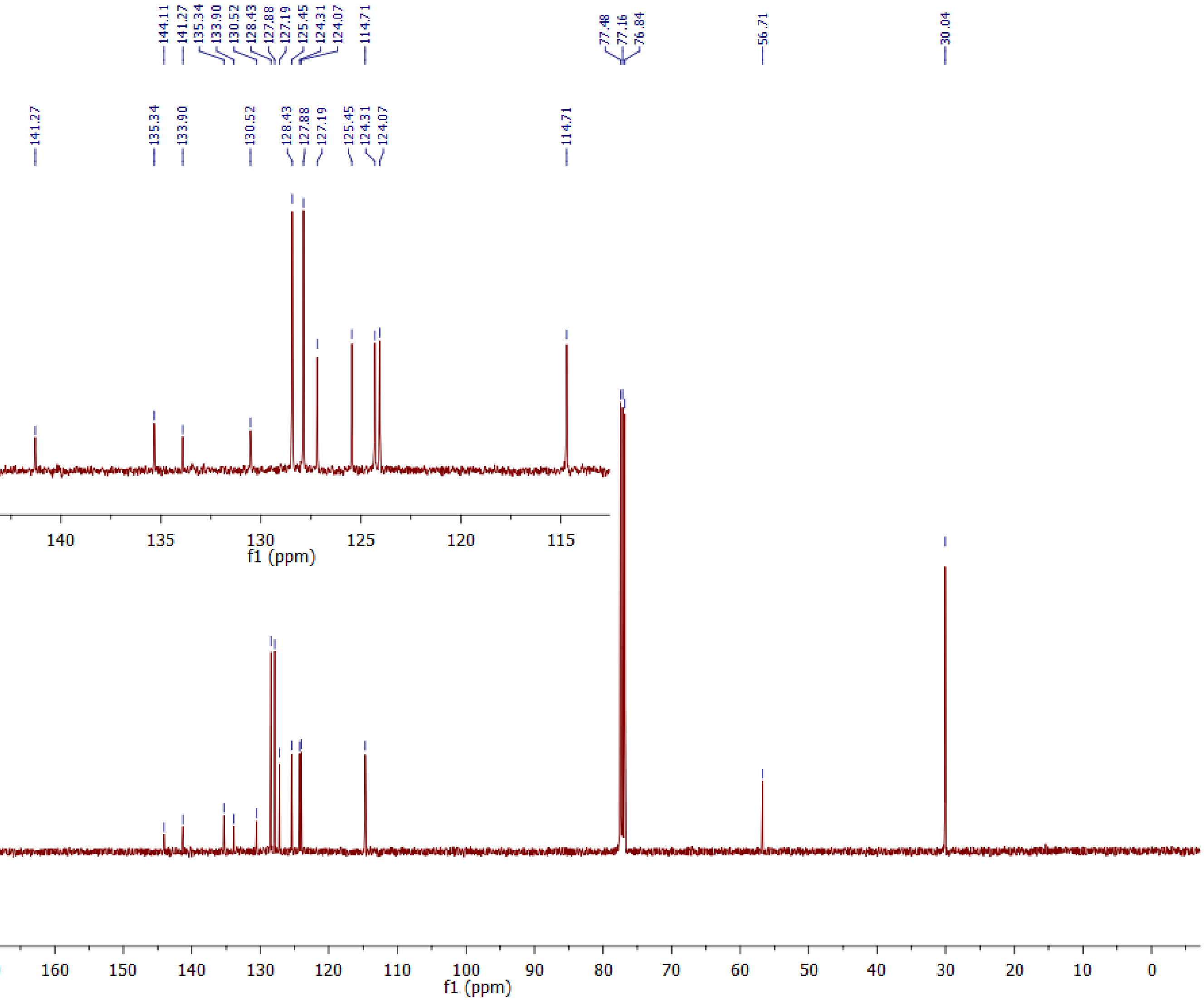

Figure S39. ^{1}H NMR spectra of **3b** (400 MHz, $CDCl_3$, δ).

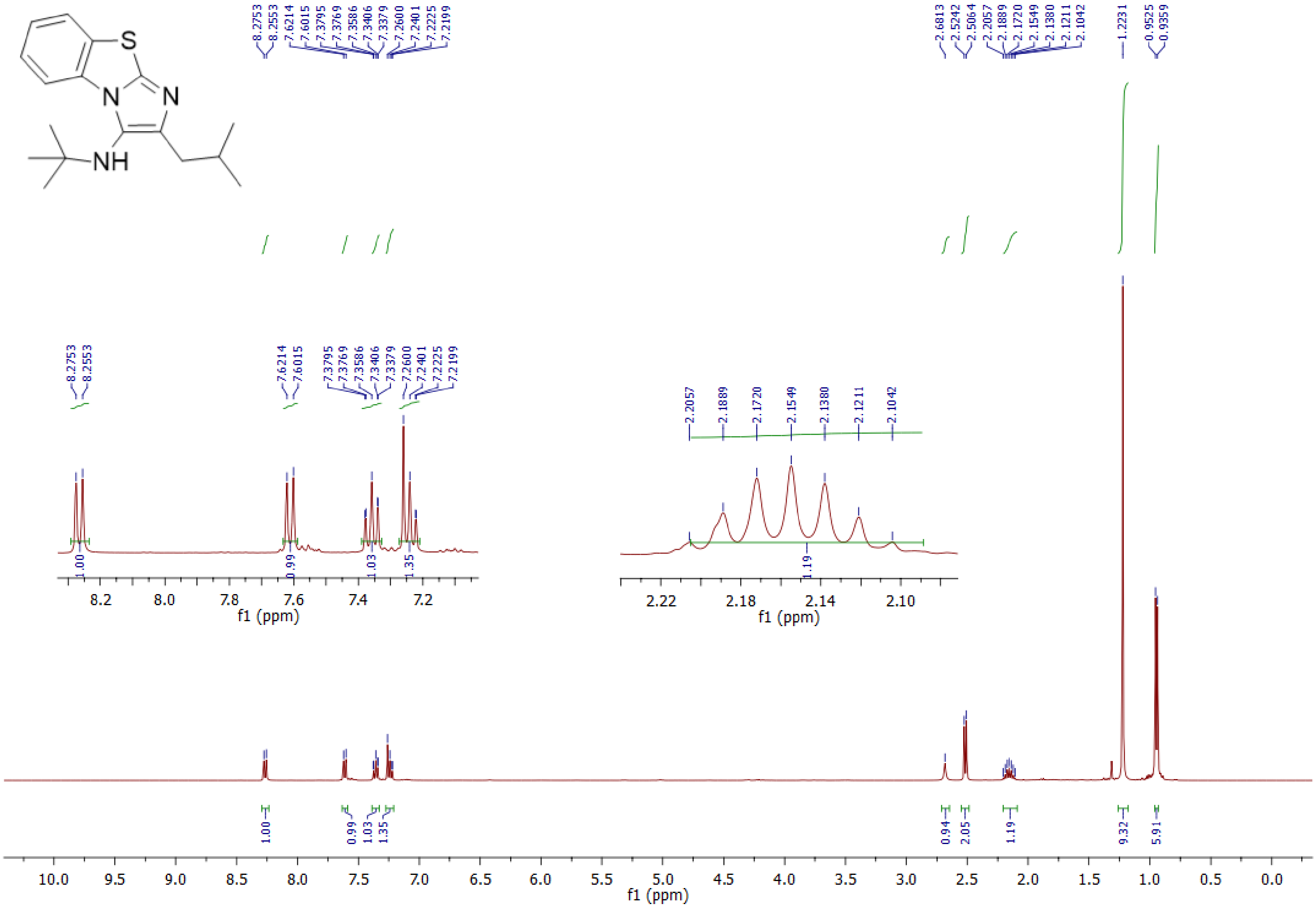

Figure S40. ^{13}C NMR spectra of **3b** (100 MHz, $CDCl_3$, δ).

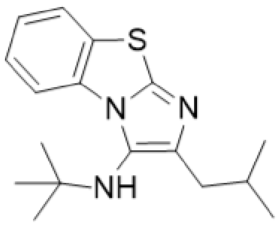

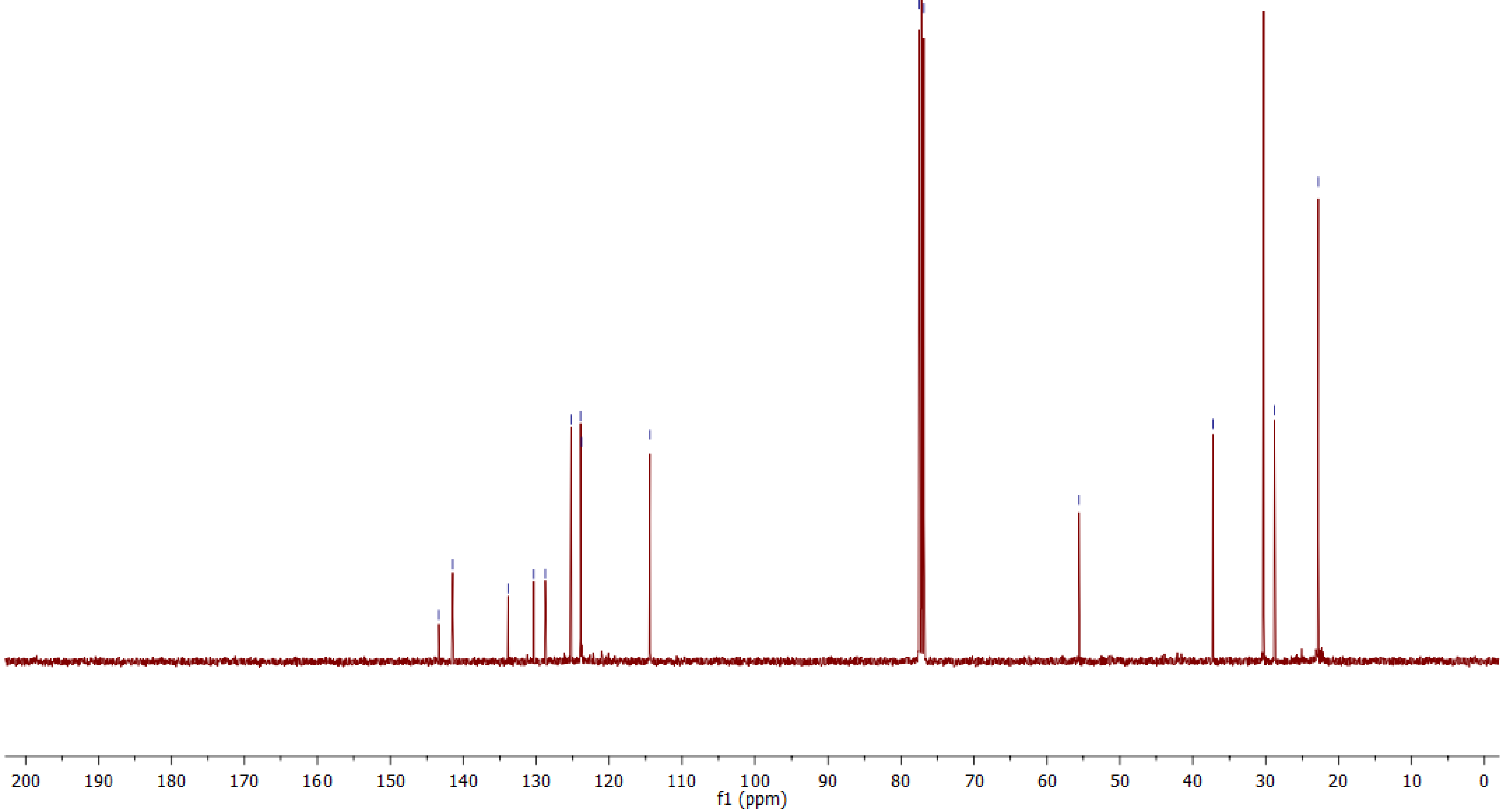

Figure S41. ^{1}H NMR spectra of **3c** (300 MHz, $CDCl_3$, δ).

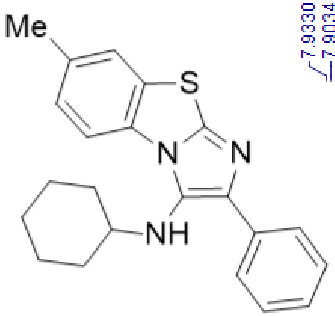

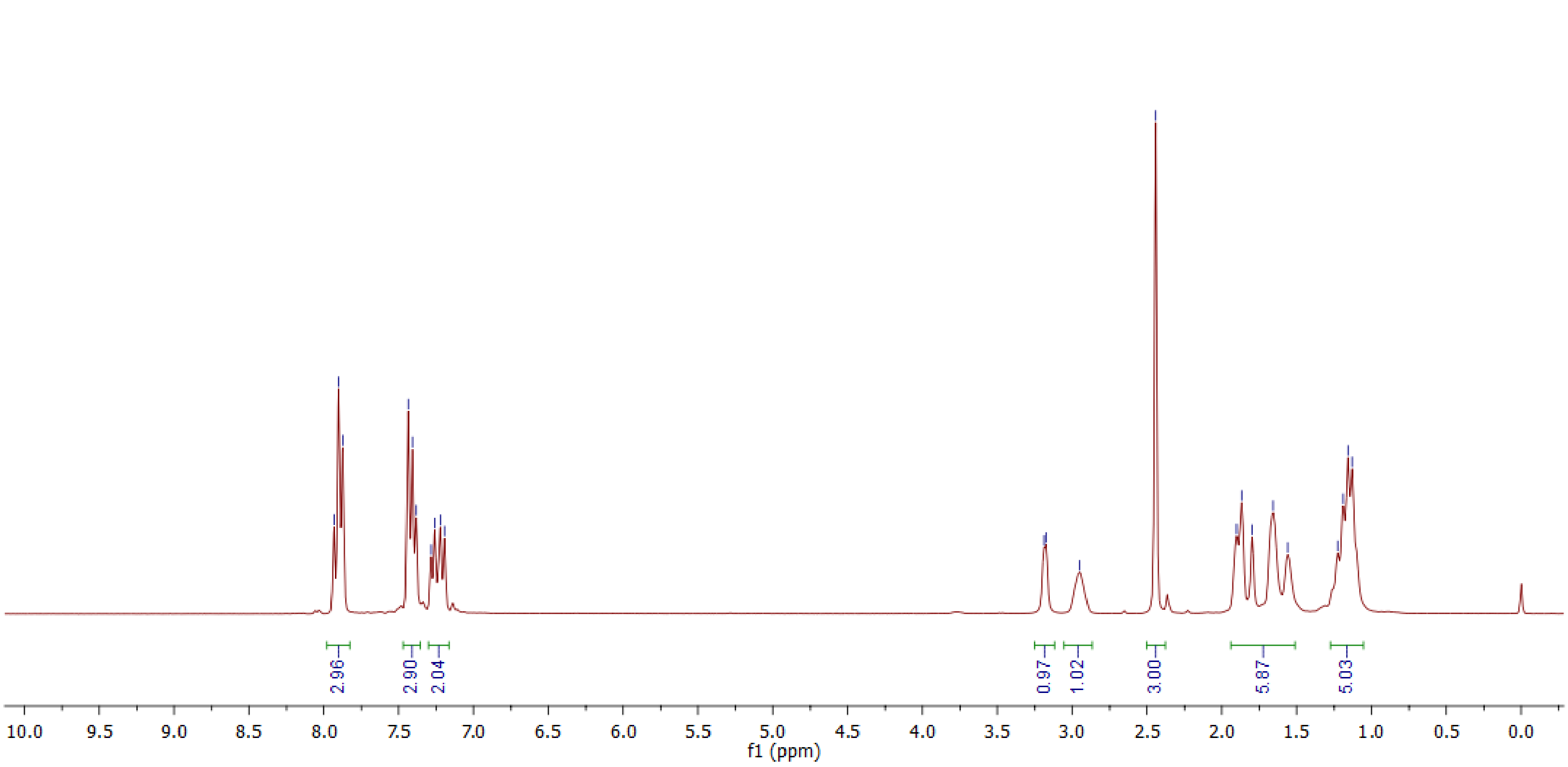

Figure S42. ^{13}C NMR spectra of **3c** (75 MHz, $CDCl_3$, δ).

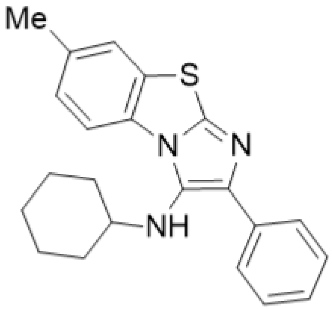

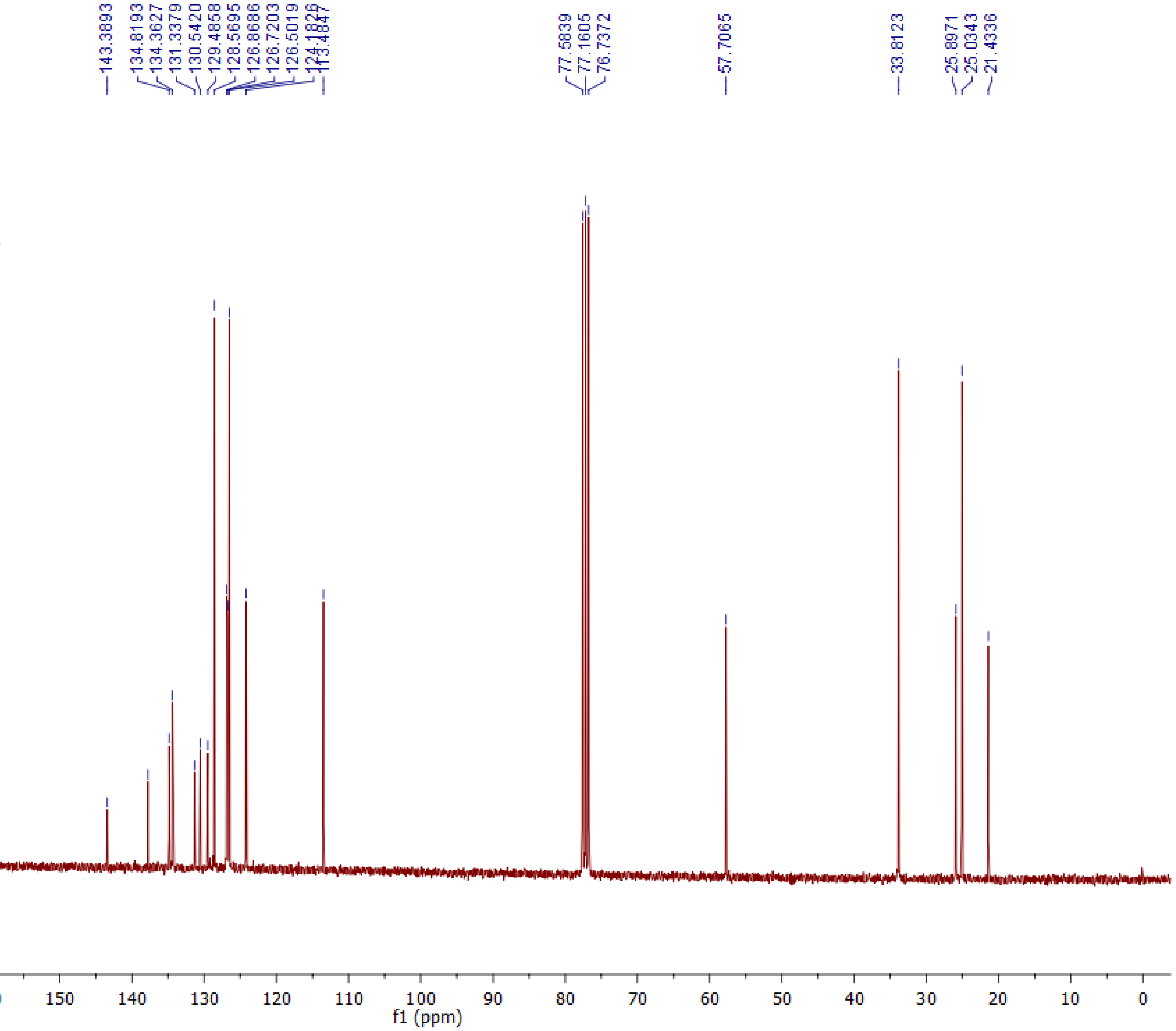

Figure S43. ^{1}H NMR spectra of **3d** (400 MHz, CDCl₃, δ).

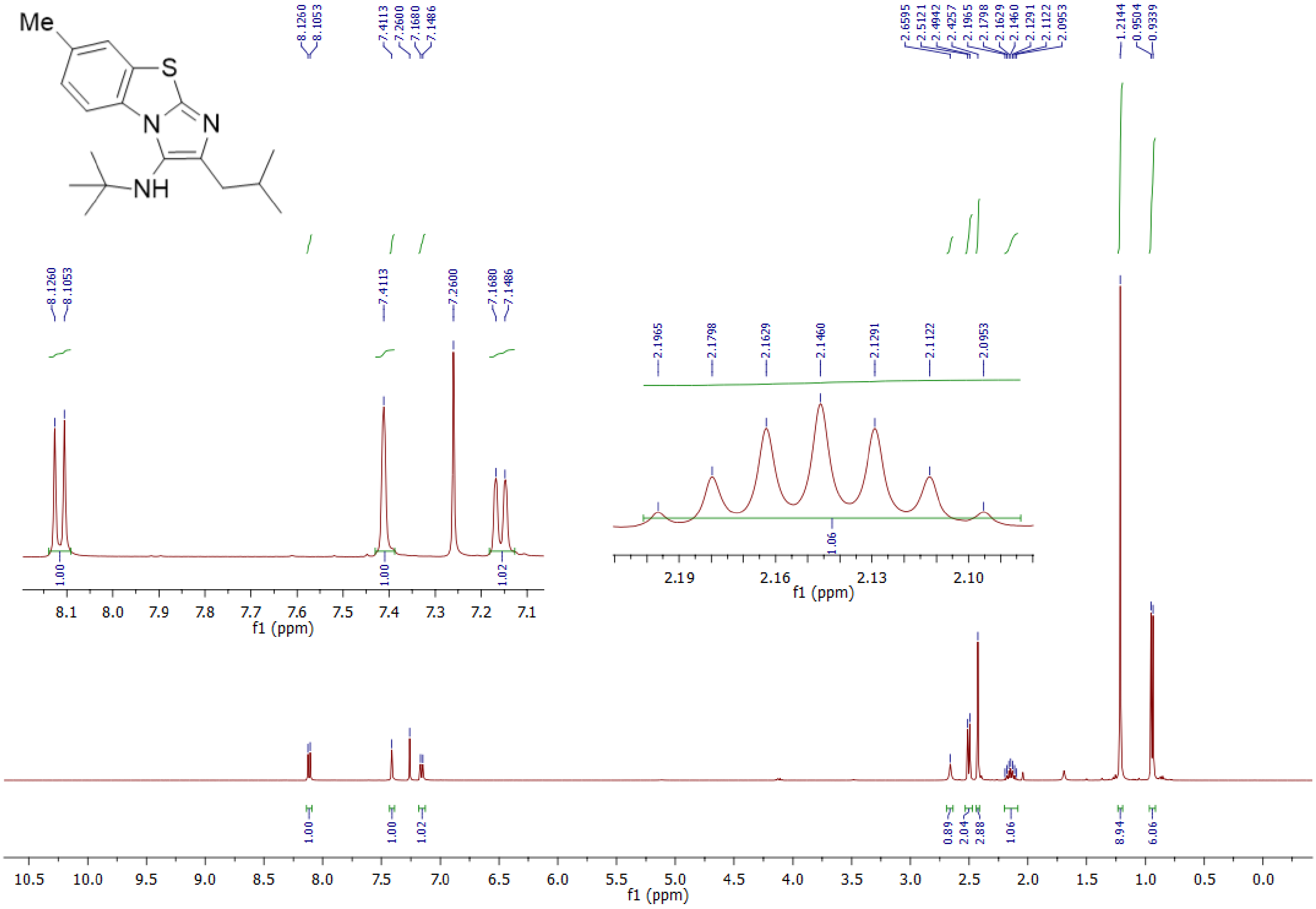

Figure S44. ^{13}C NMR spectra of **3d** (100 MHz, CDCl₃, δ).

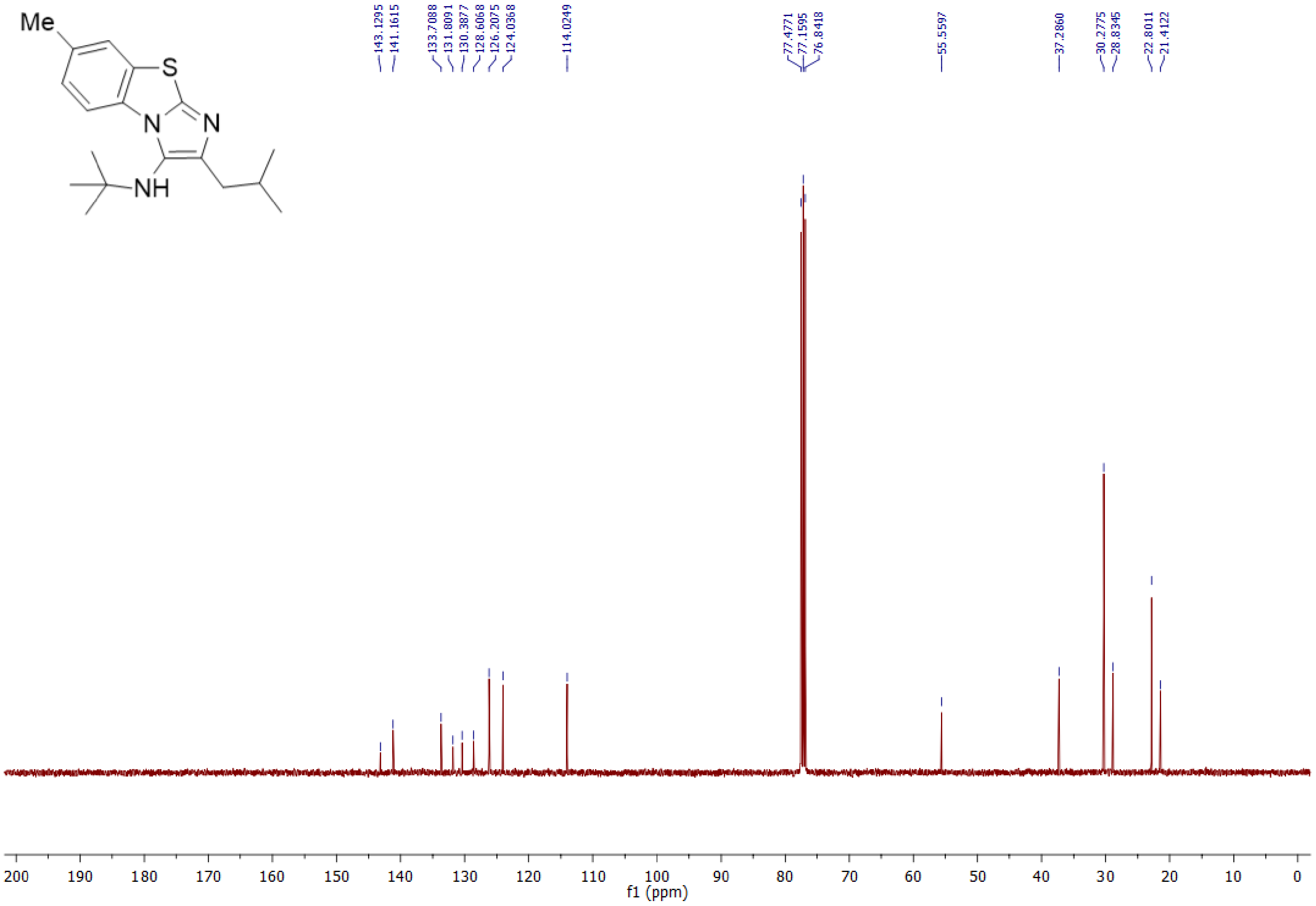

Figure S45. ^{1}H NMR spectra of **3e** (300 MHz, $CDCl_3$, δ).

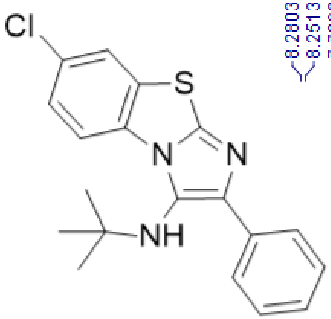

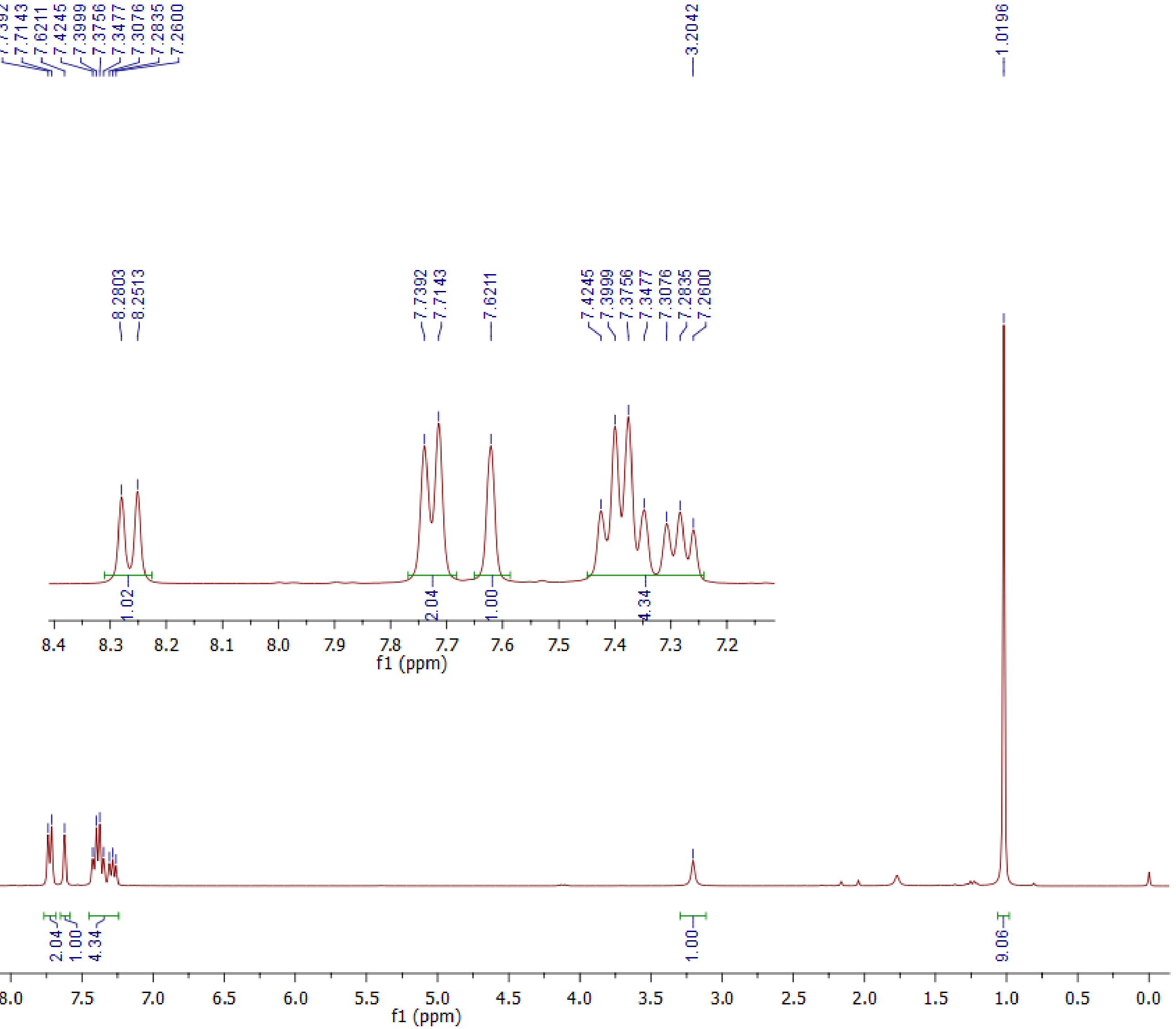

Figure S46. ^{13}C NMR spectra of **3e** (75 MHz, $CDCl_3$, δ).

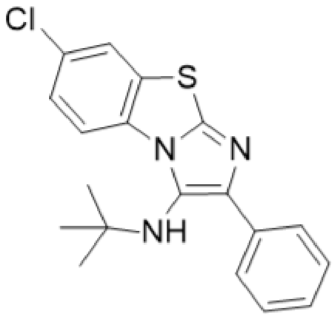

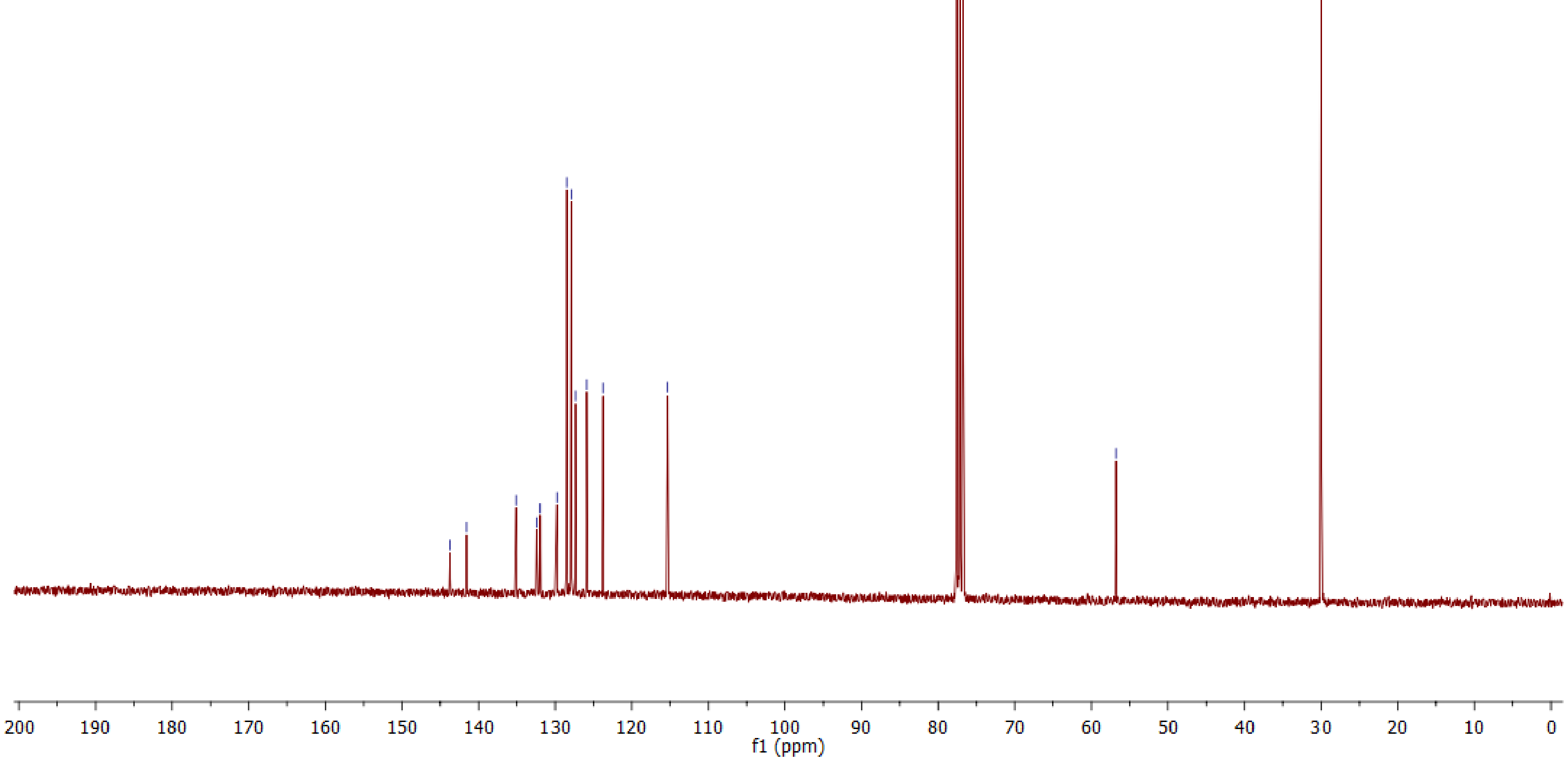

Figure S47. ^{1}H NMR spectra of **3f** (400 MHz, CDCl₃, δ).

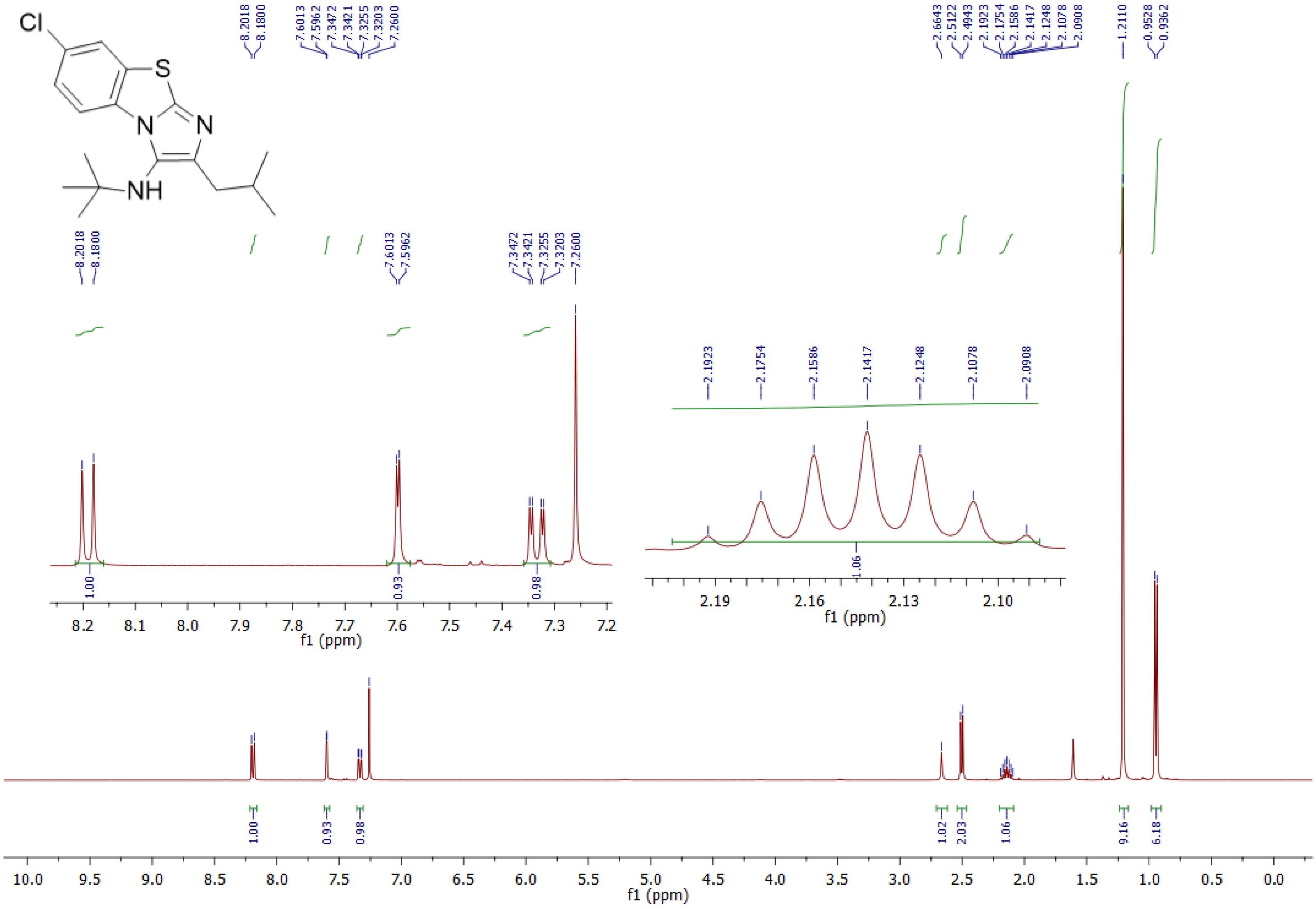

Figure S48. ^{13}C NMR spectra of **3f** (100 MHz, $CDCl_3$, δ).

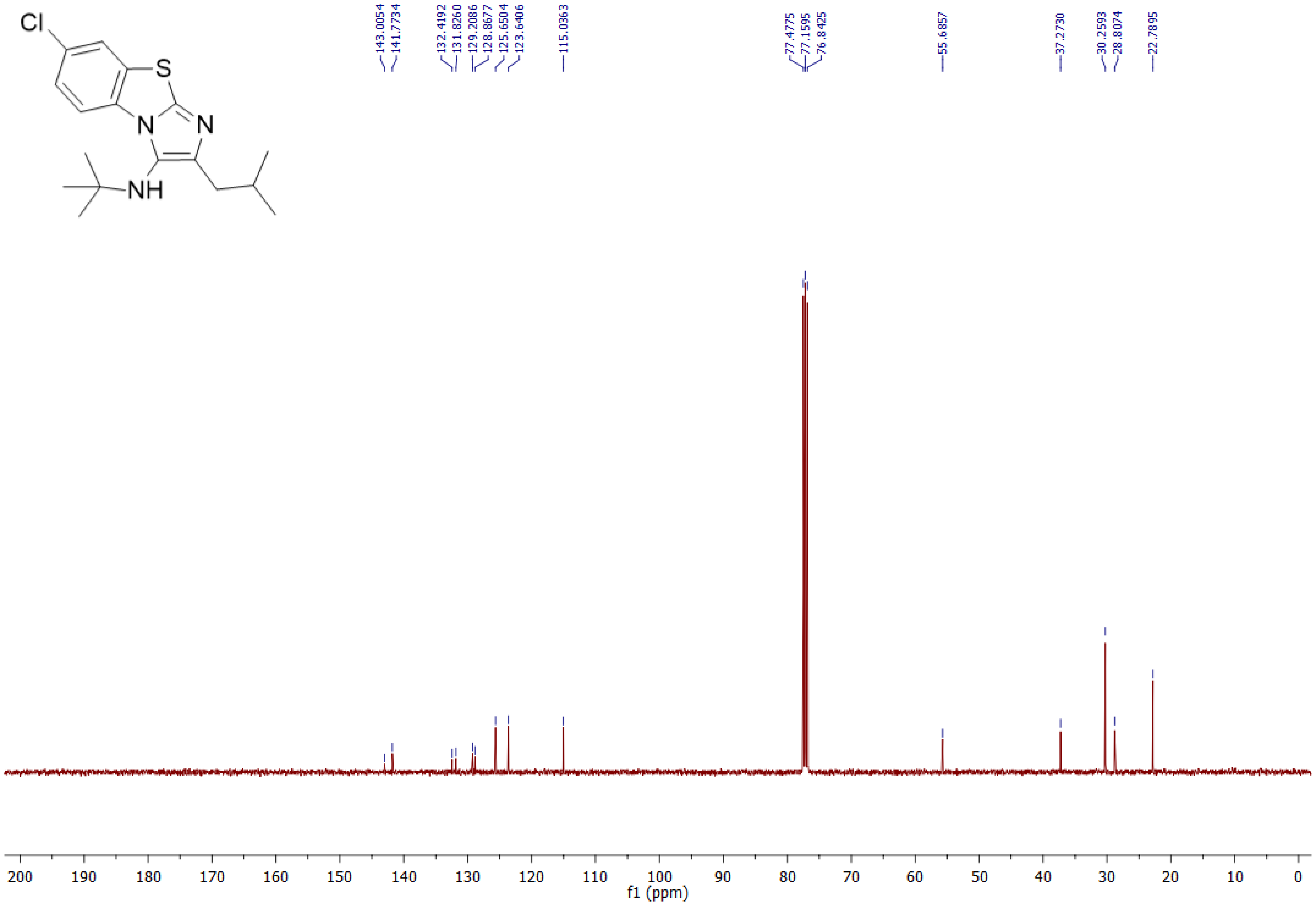

Supplement: Supplementary file 1 [file ao6c03507_si_001.pdf]
